# Transition Metal-Free Synthesis of Halobenzo[*b*]furans from *O*-Aryl Carbamates via *o*-Lithiation Reactions †

**DOI:** 10.3390/molecules27020525

**Published:** 2022-01-14

**Authors:** Claudia Feberero, Cintia Virumbrales, Carlos Sedano, Lorena Renedo, Samuel Suárez-Pantiga, Roberto Sanz

**Affiliations:** Área de Química Orgánica, Departamento de Química, Facultad de Ciencias, Universidad de Burgos, Pza. Misael Bañuelos s/n, 09001 Burgos, Spain; cfeberero@ubu.es (C.F.); cvirumbrales@ubu.es (C.V.); cslabrador@ubu.es (C.S.); lrp1003@alu.ubu.es (L.R.); svsuarez@ubu.es (S.S.-P.)

**Keywords:** *o*-lithiation, carbamates, alkynylation, benzo[*b*]furans, cyclization

## Abstract

A straightforward and transition metal-free one-pot protocol to synthesize halobenzo[*b*]furans has been developed employing simple and easily available starting materials such as *O*-aryl carbamates and alkynylsulfones. The fine-tuning of the different steps involved was key to achieving a successful one-pot procedure. Initially, a directed *ortho*-lithiation process, which uses the carbamate as the directed metalation group, was crucial in providing access to *O*-2-alkynylaryl *N,N*-diethyl carbamates by a direct alkynylation of the *o*-lithiated carbamate, with arylsulfonylalkynes as electrophilic reagents. Cyclization of the generated *o*-alkynylaryl carbamates was successfully accomplished through a strategy involving in situ carbamate alkaline hydrolysis under conventional heating or microwave irradiation, coupled with a subsequent heterocyclization step delivering the desired benzo[*b*]furans. A wide variety of new halobenzo[*b*]furans has been synthesized and their utility has been demonstrated by their further transformation.

## 1. Introduction

Benzo[*b*]furans are an important class of heterocyclic compounds that show a wide range of biological properties with medicinal significance [1,2]. They are also common structural motifs of natural products and bioactive compounds [3], as well as bioisosteres of indoles [4]. Thus, there is a growing interest in developing general and versatile methodologies for synthesizing molecules bearing this heterocyclic scaffold [5,6,7]. Among them, tandem Sonogashira coupling/cyclization from alkynes and *o*-halophenols under Pd-catalysis is one of the most used and efficient procedures [8]. In this field, the regioselective preparation of halogen-functionalized benzo[*b*]furans is a relevant goal, as these substrates allow for further functionalization through a plethora of transition metal-catalyzed cross-couplings or halogen-lithium exchange and subsequent electrophilic trapping. Whereas 5- and 7-halobenzo[*b*]furans can be prepared easily by different cyclization of *p*- and *o*-halophenol derivatives [9], respectively, the selective synthesis of 4-halobenzofurans is challenging to achieve, as the use of *m*-halophenols typically affords mixtures of 4- and 6-halobenzofurans [10,11]. In addition, starting from preformed benzofurans, its *C*-4 position is the least nucleophilic one and not prone to undergo an aromatic electrophilic substitution reaction.

**Scheme 1:**
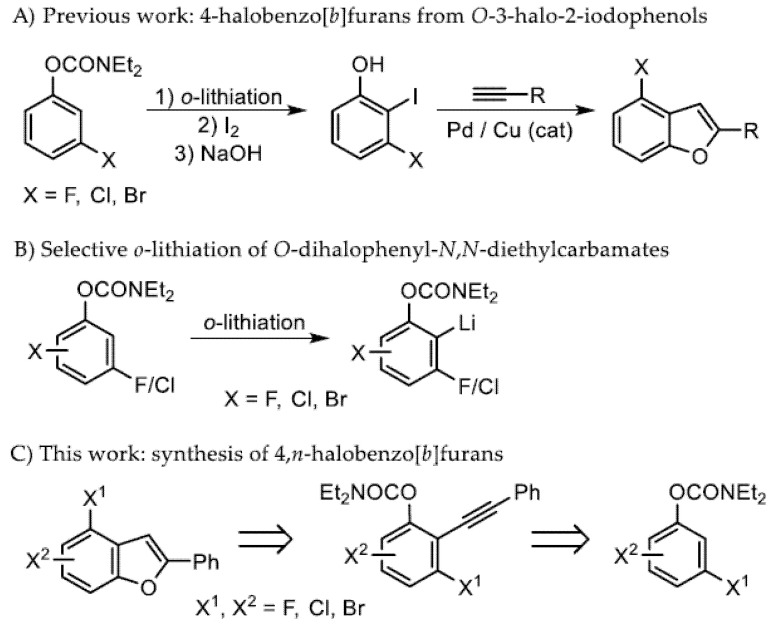
(**A**) Our previous work. (**B**) Selective *o*-lithiation of *O*-dihalophenyl-*N*,*N*-diethylcarbamates. (**C**) Proposed retrosynthesis for dihalobenzo[*b*]furans [17,18].

On the other hand, directed *ortho*-lithiation is a powerful methodology in organic synthesis for the regioselective functionalization of (hetero)aromatics [12,13], and the search for new directed metalation groups is a topic actively explored [14,15,16]. In 2005, our group described a tandem Sonogashira coupling/5-*endo*-*dig* cyclization reaction of 2-iodo-3-halophenols, which were prepared through the regioselective *ortho*-lithiation of *N,N*-diethyl *O*-3-halophenylcarbamates [17]. This approach supposed a straightforward entry to 4-halobenzo[*b*]furans, which could be subsequently transformed into 4-functionalized benzo[*b*]furan derivatives (Scheme 1A). More recently, we have expanded the scope of the regioselective lithiation of *O*-dihalophenyl-*N*,*N*-diethylcarbamates, which was found to be regioselective at the C-2 position due to the cooperative effect of the *m*-halide and the mighty carbamate-directed metalating group [18] (Scheme 1B).

Although our route to access 4-halobenzo[*b*]furans was practical and efficient, starting from readily available halophenyl carbamates, it suffers from the use of transition metal complexes as well as the need for an iodine atom at the *ortho*-position, which is subsequently consumed in the Sonogashira coupling. In this context, and following our interest in the development of synthetic strategies based on the applications of directed *ortho* metalation reactions [19,20,21,22], we reasoned that if we were able to prepare *O*-2-alkynylaryl *N*,*N*-diethycarbamates in a straightforward manner from the intermediate *O*-2-lithioaryl carbamates, we would circumvent the prior synthesis and isolation of an iodoaryl carbamate. In addition, we also envisaged that the subsequent alkaline hydrolysis of the carbamate group in the intermediate 2-alkynylaryl carbamates would deliver a phenoxide that could undergo in situ cyclization providing the corresponding halobenzo[*b*]furans (Scheme 1C). Herein, we report a straightforward and transition metal-free synthesis of a wide variety of regioselectively halo-functionalized benzo[*b*]furans from readily available haloaryl carbamates, taking advantage of the powerful *O*-carbamate-directed metalation methodology pioneered by Prof. Snieckus [23,24,25].

## 2. Results and Discussion

### 2.1. Synthesis of O-o-Alkynylaryl Carbamates ***2***

**Scheme 2:**
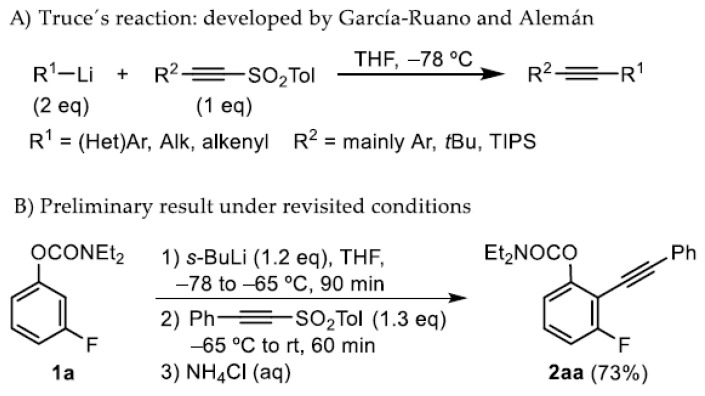
(**A**) Truce reaction developed by Alemán and García-Ruano. (**B**) Synthesis of *O*-2-alkynylaryl *N,N*-diethyl carbamate **2aa [27,28]**.

To tackle the proposed target, we first needed to establish a suitable protocol for synthesizing *O*-*o*-alkynylaryl *N*,*N*-diethyl carbamates. We planned to take advantage of the Truce reaction [26], more recently developed and expanded by Alemán and García-Ruano [27,28], involving the use of arylsulfonylacetylenes as alkynylating reagents for organolithium and organomagnesium compounds. Alemán and García Ruano established that readily available alkynyl sulfones [29] undergo an “anti-Michael” addition of selected organolithiums, leading to their direct alkynylation. This unexpected behavior was rationalized with the aid of computational studies to proceed through an initial association of the organolithium to the sulfone prior to its intramolecular attack (Scheme 2A).

In the procedure established by these authors, an excess of the corresponding organolithium derivative (2 equiv) was made to react with the alkynylsulfone [28]. In our case, we decided to revisit the reported conditions to use the starting carbamate as the limiting reagent. After some experimentation, we observed that only slight excess of the alkynylsulfone was required as the electrophilic partner for the organolithium intermediate **1a-Li** generated from the regioselective *ortho*-lithiation of *O*-3-fluorophenyl *N,N*-diethylcarbamate **1a** [17,18]. Under these conditions, the *O*-2-alkynyl-3-fluorophenyl carbamate **2aa** could be isolated in good yield, referred to as the starting carbamate **1a** (Scheme 2B).

Starting from selected carbamates **1**, we achieved lithiation at the *ortho*-position under our previously described reaction conditions [18]. Then, the generated organolithium intermediate was reacted with the corresponding alkynylsulfone, affording alkynyl-functionalized carbamates **2**, which could be isolated with good yields (Table 1).

We tested a range of carbamates with halogens (F and Cl) located at different positions of the benzene ring. The reaction provided access to 2-alkynyl 3-halocarbamates (**2aa** and **2da**) (entries 1 and 4). In addition, 2-alkynyl-3,4-dihalophenyl carbamates were also possible to obtain (entry 2), as well as 3,5- and 3,6-dihalo-substituted ones (entries 3 and 5, respectively). Next, we proceeded to test this alkynylation reaction using different arylalkynyl sulfones. Interestingly, other arylsulfonylacetylenes bearing electron-donating or withdrawing groups on the aryl fragment were successfully employed in the reaction, leading to 2-alkynyl carbamates **2ab, ac, dc** (entries 6–8). However, the reaction of **1a** with alkynylsulfones bearing 3-thienyl, cyclopropyl, or *n*-butyl substituents failed in our hands, likely due to competitive acid-base reactions.

### 2.2. Synthesis of 4-Halo and 4,n-Dihalobenzo[b]furans ***3***

#### 2.2.1. Preliminary Results

**Scheme 3:**
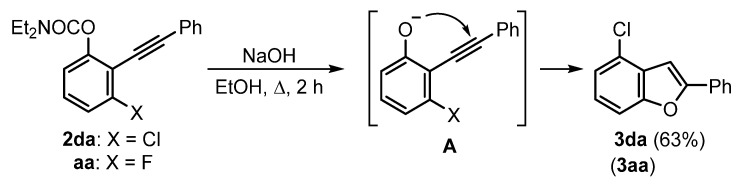
Preliminary results for the hydrolysis–cyclization of carbamates **2aa** and **2da**.

Having demonstrated the efficient preparation of *O*-halo-functionalized *o*-alkynylaryl carbamates **2**, we envisioned that these substrates could experience cyclization, affording valuable halo-functionalized benzo[*b*]furans **3** under transition metal-free reaction conditions. Considering the known alkaline hydrolysis of *O*-aryl *N,N*-diethyl carbamates [17,30] and the NaOH-mediated cyclization processes of *o*-alkynyl anilines resulting in indoles [31], we selected **2da** as the model substrate to study a cascade reaction involving its hydrolysis to the corresponding phenoxide intermediate **A**, and its subsequent cyclization, by using an excess of NaOH (Scheme 3). Gratifyingly, we found that 4-chloro-2-phenylbenzo[*b*]furan **3da** was isolated with good yield after 2 h in refluxing ethanol.

Surprisingly, when we subjected the fluoro-functionalized carbamate **2aa** to the same conditions, the reaction did not occur (Scheme 3). At this point, we decided to further study the desired hydrolysis–cyclization of **2aa** by evaluating several parameters, such as the base, solvent, time, temperature, and heating method.

#### 2.2.2. Optimization of the Reaction Conditions

With **2aa** as the model substrate, different bases were employed, such as cesium carbonate, potassium carbonate, lithium aluminum hydride [32], triethylamine, sodium hydroxide, and potassium hydroxide (Table 2, entries 1–7). None of them achieved the expected cyclization reaction under different reaction conditions. However, when we employed NaOH in EtOH, heated the reaction mixture at 60 °C, and extended the reaction time to 48 h, we observed complete conversion, with the expected benzo[*b*]furan **3aa** the only compound identified from the mixture (entry 8). To reduce the reaction time, we assayed different solvents that could allow us to increase the reaction temperature. By using DMF or DMSO, the reaction time could be shortened to 18 and 12 h, respectively (entries 9 and 10). At this point, we evaluated the influence that the heating method could have. For this purpose, we changed from conventional heating to microwave irradiation. Gratifyingly, when DMA was used as the solvent, the reaction took place in 40 min at 160 °C under microwave irradiation, with almost complete conversion (entry 11). In addition, KOH was proved to afford similar results to those employing NaOH. Thus, the best results were obtained following either of these two methods:

Method A: NaOH (2 eq) in DMSO (0.5 M) by conventional heating at 140 °C.

Method B: NaOH (2 eq) in DMA (0.5 M) by microwave irradiation at 160 °C.

#### 2.2.3. One-Pot Preparation of Halo-Functionalized Benzofurans **3** from *O*-Haloaryl *N*,*N*-Diethyl Carbamates **1**

Before studying the scope of this process to prepare benzofuran derivatives **3**, we decided to unify both reactions, the alkynylation of *o*-lithiated carbamates **1** and the hydrolysis–cyclization of *o*-alkynyl carbamates **2**, in a one-pot two-step sequence to avoid the isolation and purification of intermediates **2**. We could successfully carry out the proposed one-pot process and apply it to the synthesis of a wide range of 4-halo and 4, *n*-dihalo-2-arylbenzo[*b*]furans **3** (Table 3). The transformation was shown to be general and could be extended to a wide variety of *O*-(di)halophenyl *N,N*-diethyl carbamates, allowing for the transition metal-free synthesis of relevant halobenzo[*b*]furans. However, LDA was used as a metalating agent instead of *s*-BuLi, with bromo-functionalized starting carbamates **1j, k, m,** and **n** to avoid the competitive Br–Li exchange reaction [17] (entries 15, 16, 18, and 19). It is worth noting that the resulting benzofurans **3** were obtained in moderate to good yields in a straightforward manner from the readily available *O*-haloaryl *N,N*-diethyl carbamates **1**. We observed, as a general effect, that substrates bearing a fluorine substituent led to lower yields, which could be due to a higher stabilization of the intermediate phenoxide anion, thus making it less reactive in the cyclization step.

### 2.3. Synthesis of 5-Functionalized Benzo[b]furans ***4***

In this context, we planned to broaden the scope of the developed methodology to benzofurans with another pattern of functionalization and demonstrate that the 3-halo substituent is not required for the reaction to proceed. Although the preparation of 5-functionalized benzofurans is well-resolved starting from *p*-halophenol derivatives, as was established in Section 1, we tried to synthesize a selection of these heterocyclic derivatives by making use of the strategy described above. In this sense, a selection of 4-functionalized aryl carbamates **1o-q** were used as the starting materials (Table 4). The one-pot alkynylation of the *o*-lithiated carbamate/hydrolysis–cyclization was proven to be successful, and 5-functionalized benzo[*b*]furan derivatives **4** could be isolated in moderate yields. In these cases, the second step was only performed under microwave irradiation, given the shorter reaction times.

### 2.4. Synthesis of 3-Alkynylsalicylamides ***5*** from O-3-Halophenyl Carbamates ***1***

**Scheme 4:**
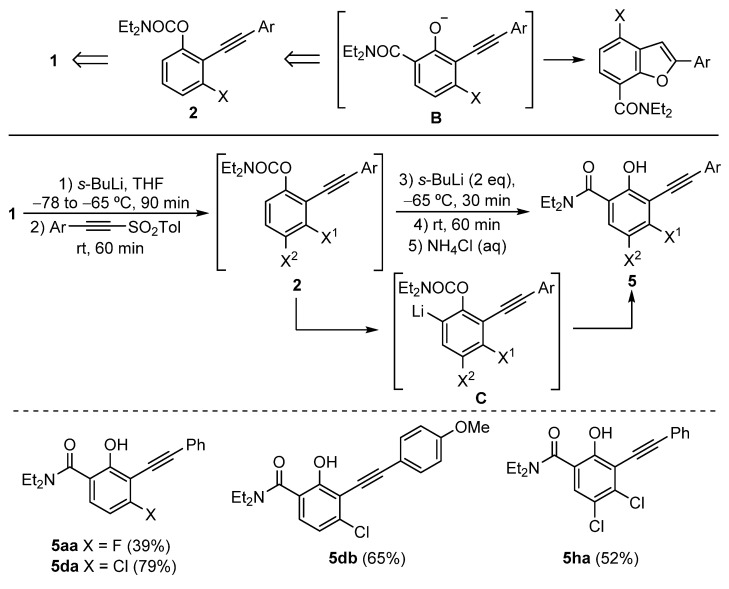
Synthesis of 3-alkynylsalicylamides **5**.

After developing an efficient strategy to obtain halobenzo[*b*]furans from readily *O*-halophenyl *N*,*N*-diethyl carbamates **1**, we envisaged that an alternative pathway to generate in situ an *o*-alkynyl phenoxide intermediate, like **A** (see Scheme 3), could be to trigger a Snieckus–Fries rearrangement from intermediates *O*-*o*-alkynylphenyl carbamates **2**, after further addition of the base, finally leading to phenoxides **B**. In this way, new 7-diethylcarbamoyl halobenzofurans could be accessed (Scheme 4). Taking advantage of our previously reported synthesis of dihalosalicylamides through the Snieckus–Fries rearrangement [18], we attempted a one-pot tandem alkynylation of an *o*-lithiated carbamate/anionic Fries rearrangement/cyclization sequence with a selection of the starting carbamates **1a, d,** and **h** (Scheme 4). In all the cases, the 3-alkynylsalicylamides **5** were obtained in moderate to good yields without the formation of the desired benzofuran derivatives. These results imply that the two first processes of the planned sequence, alkynylation/Snieckus–Fries rearrangement, had taken place, but not the final cyclization to the heterocyclic scaffold. After the initial alkynylation reaction that provided **2**, the subsequent addition of *s*-BuLi led to a new *o*-lithiated carbamate **C** that underwent the anionic rearrangement upon raising the reaction temperature (Scheme 4). The lower yield obtained for 3-alkynylsalicylamide **5aa** could be due to a competitive lithiation *ortho* to the fluorine atom in the corresponding intermediate **2aa**. Disappointingly, attempts to trigger the subsequent cyclization of alkynylsalicylamides **5** under a wide variety of reaction conditions were not successful, likely due to the delocalization of the negative charge caused by the amide located at the *ortho* position.

### 2.5. Synthesis of o,o′-Dialkynyl Carbamates ***7*** from O-3-Chloro-2-Iodophenyl Carbamate ***1r***

**Scheme 5:**
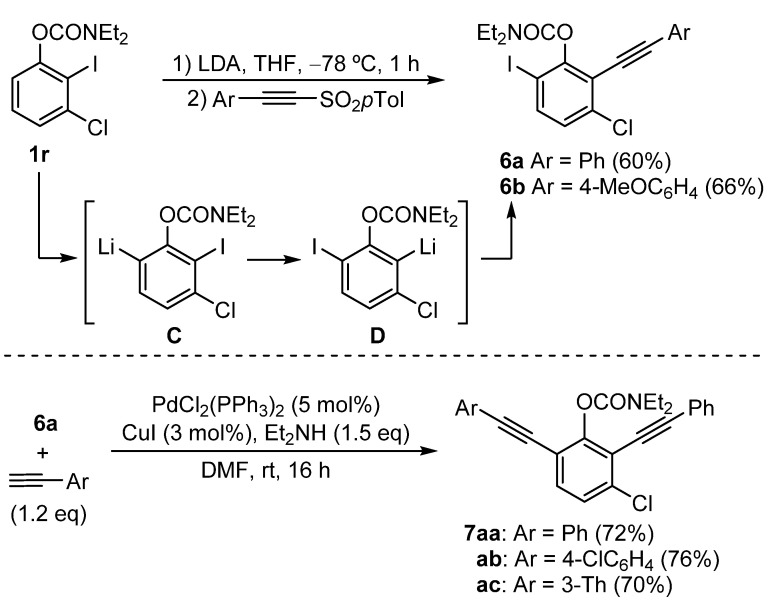
Synthesis of *o,o′*-dialkynyl carbamates **7**.

In a complementary way, we decided to prepare carbamates with two alkynyl groups at the *ortho* positions. To this end, 3-chloro-2-iodophenyl carbamate **1r** [17] was subjected to a combined *o*-lithiation-halogen dance sequence developed by Snieckus and co-workers [33], which takes place through organolithium intermediates **C** and **D** (Scheme 5). By applying this strategy, 6-alkynyl-5-chloro-2-iodophenyl carbamates **6** were obtained in good yields after trapping **D** with arylsulfonylacetylenes. With these substrates **6** in hand, we carried out standard Sonogashira couplings with a selection of terminal alkynes, which led to the formation of 2,6-dialkynylphenyl carbamates **7** (Scheme 5). Regretfully, attempts to carry out the subsequent cyclization of carbamates **7** under the previously established conditions did not lead to the desired benzofurans. Even the hydrolysis step was unsuccessful, only recovering starting materials or decomposition products when employing harsher conditions.

### 2.6. Further Transformations of 4,n-Dihalobenzo[b]furans ***3***

**Scheme 6:**
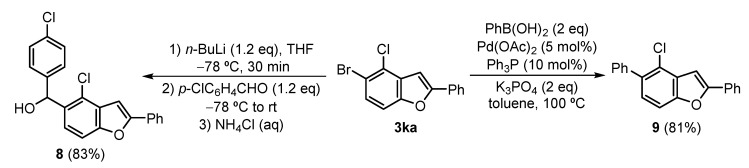
Further transformations of benzofuran **3ka**.

Finally, we decided to check the usefulness of 4,*n*-dihalobenzo[*b*]furans **3** as precursors of 4,*n*-difunctionalized benzo[*b*]furans. In this case, benzofuran **3ka** was selected for further transformations. Firstly, **3ka** underwent Br–Li exchange by treatment with *n*BuLi at a low temperature, and the generated organolithium was subsequently trapped with *p*-chlorobenzaldehyde, affording alcohol **8** in high yield (Scheme 6). In addition, bromobenzo[*b*]furan **3ka** proved to be a useful starting material for Suzuki Pd-catalyzed coupling. Thus, 4-chloro-2,5-diphenylbenzo[*b*]furan **9** was readily accessible from **3ka** by its reaction with phenylboronic acid (Scheme 6).

## 3. Materials and Methods

### 3.1. General Information

All reactions involving air-sensitive compounds were carried out under a nitrogen atmosphere (99.99%). All glassware was oven-dried, evacuated, and purged with nitrogen. All common reagents and solvents were obtained from commercial suppliers and used without any further purification. NMR spectra were recorded at 300 MHz for ^1^H NMR, and 75.4 MHz for ^13^C NMR with a Varian Mercury-Plus (Agilent Technologies, Inc.; Santa Clara, CA, USA) or a Bruker Avance spectrometer (Bruker Corporation, Billerica, MA, USA) at room temperature. The NMR data are reported as: (s = singlet, d = doublet, t = triplet, q = quartet, m = multiplet or unresolved, br s = broad signal; coupling constant(s) in Hz, integration). ^13^C NMR spectra were recorded with ^1^H-decoupling and referenced to the solvent signal. DEPT experiments were performed to assign C, CH, CH_2_, and CH_3_ signals. ^1^H and ^13^C spectra for compounds **2–9** are available from the Supplementary Materials. High-resolution mass spectra (HRMS) were obtained on an Agilent 6545 Q-TOF mass spectrometer (Agilent Technologies, Inc.; Santa Clara, California, USA) using electrospray ionization (ESI+) or using an atmospheric pressure chemical ionization source (APCI+). Low-resolution mass spectra (LRMS) measurements were recorded on an Agilent 6890N/5973 Network GC System equipped with an HP-5MS column using electronic impact (EI) or on a Thermo Scientific Trace 1300 Series Gas Chromatograph (Thermo Fisher Scientific Inc.; Waltham, Massachusetts, USA) coupled with an ISQ Single Quadrupole Mass Spectrometer. Thin-layer chromatography (TLC) (Merck KGaA, Darmstadt, Germany) was performed on aluminum-backed plates coated with silica gel 60 with an F254 indicator; chromatograms were visualized under ultraviolet light. *R*_f_ values are reported on silica gel. Flash column chromatography was carried out on silica gel 60, 230–240 mesh. Melting points were measured on a Gallenkamp apparatus (Gallenkamp & Co, London, United Kingdom) using open capillary tubes and are uncorrected. Starting carbamates **1** were previously reported by our group [17,18], whereas the alkynyl sulfones were prepared following the described procedure [29].

### 3.2. Preparation and Characterization Data of Compounds

#### 3.2.1. Synthesis of *O*-2-(Arylethynyl)phenyl *N*,*N*-Diethylcarbamates ***2***

General procedure: A solution of starting carbamate **1** (1 mmol) in THF (2 mL) at −78 °C under nitrogen atmosphere was treated with a solution of *s*-BuLi (0.85 mL of a 1.4 M solution in cyclohexane, 1.2 mmol). The reaction mixture was allowed to reach −65 °C for 5 min and was then stirred at this temperature for 90 min. Then, the corresponding alkynyl sulfone [29] (1.3 mmol) was added, and the resulting solution was stirred for 15 min at −65 °C. The solution was allowed to warm to room temperature and was then stirred for an additional 30 min. The reaction mixture was quenched with NH_4_Cl (aq), diluted with EtOAc, and the layers were separated. The aqueous phase was extracted with EtOAc (3 × 10 mL), and the combined organic layers were dried over anhydrous Na_2_SO_4_. The solvents were removed under reduced pressure, and the residue was purified by silica gel (VWR chemicals, Radnor, PA, USA) column chromatography (hexane/EtOAc), affording the *O*-2-(arylethynyl)phenyl *N*,*N*-diethylcarbamates **2**.

*O-3-Fluoro-2-(phenylethynyl)phenyl N,N-diethylcarbamate* (**2aa**): The reaction of *O*-3-fluorophenyl *N*,*N*-diethylcarbamate (**1a**) (221 mg, 1 mmol), following the general procedure, yielded the product as a yellow oil (73% yield); *R*_f_ = 0.26 (hexane/EtOAc, 5:1). ^1^H NMR (300 MHz, CDCl_3_) δ (ppm): 7.55–7.52 (m, 2H), 7.38–7.28 (m, 4H), 7.09 (dt, *J* = 8.3, 1.0 Hz, 1H), 7.00 (td, *J* = 8.3, 1.0 Hz, 1H), 3.54 (q, *J* = 7.0 Hz, 2H), 3.42 (q, *J* = 7.0 Hz, 2H), 1.32 (t, *J* = 7.0 Hz, 3H), 1.20 (t, *J* = 7.0 Hz, 3H). ^13^C NMR (75.4 MHz, CDCl_3_) δ (ppm): 163.1 (d, *J* = 252.2 Hz, C), 153.32 (d, *J* = 4.1 Hz, C), 153.29 (C), 131.7 (2 × CH), 129.3 (d*, J* = 9.7 Hz, CH), 128.8 (CH), 128.4 (2 × CH), 123.0 (C), 118.5 (d, *J* = 3.3 Hz, CH), 112.3 (d*, J* = 21.0 Hz, CH), 107.4 (d*, J* = 18.0 Hz, C), 98.6 (d, *J* = 3.1 Hz, C), 78.5 (C), 42.5 (CH_2_), 42.2 (CH_2_), 14.3 (CH_3_), 13.4 (CH_3_). EI-LRMS *m*/*z* (%): 311 (M^+^, 4), 183 (7), 100 (100), 72 (56). ESI-HRMS was calculated for C_19_H_19_FNO_2_ [M + H]^+^ 312.139, and found 312.1402.

*O-4-Chloro-3-fluoro-2-(phenylethynyl)phenyl N,N-diethylcarbamate* (**2ba**): The reaction of *O*-4-chloro-3-fluorophenyl *N*,*N*-diethylcarbamate (**1b**) (245 mg, 1 mmol), following the general procedure, yielded the product as a yellow oil (75% yield); *R*_f_ = 0.29 (hexane/EtOAc, 5:1). ^1^H NMR (300 MHz, CDCl_3_) δ (ppm): 7.54–7.51 (m, 2H), 7.39–7.28 (m, 4H), 7.05 (dd, *J* = 8.9, 1.7 Hz, 1H), 3.53 (q, *J* = 7.0 Hz, 2H), 3.42 (q, *J* = 7.1 Hz, 2H), 1.31 (t, *J* = 7.1 Hz, 3H), 1.20 (t, *J* = 7.0 Hz, 3H). ^13^C NMR (75.4 MHz, CDCl_3_) δ (ppm): 158.4 (d, *J* = 254.1 Hz, C), 153.1 (C), 151.7 (d, *J* = 2.3 Hz, C), 131.7 (2 × CH), 129.6 (CH), 129.1 (CH), 128.5 (2 × CH), 122.6 (C), 119.0 (d, *J* = 4.0 Hz, CH), 117.9 (d, *J* = 17.7 Hz, C), 109.0 (d, *J* = 17.8 Hz, C), 99.7 (d, *J* = 3.7 Hz, C), 77.7 (C), 42.6 (CH_2_), 42.3 (CH_2_), 14.3 (CH_3_), 13.4 (CH_3_). EI-LRMS *m*/*z* (%): 347 (M^+^ + 2, 58), 345 (M^+^, 66), 181 (58), 100 (100), 72 (70). ESI-HRMS was calculated for C_19_H_18_ClFNO_2_ [M + H]^+^ 346.1005, and found 346.1013.

*O-5-Chloro-3-fluoro-2-(phenylethynyl)phenyl N,N-diethyl carbamate* (**2ca**): The reaction of *O*-3-chloro-5-fluorophenyl *N,N*-diethylcarbamate (**1c**) (245 mg, 1 mmol), following the general procedure, yielded the product as a yellow oil (80% yield); *R*_f_ = 0.31 (hexane/EtOAc, 5:1). ^1^H NMR (300 MHz, CDCl_3_) δ (ppm): 7.51–7.48 (m, 2H), 7.37–7.32 (m, 3H), 7.14–7.13 (m, 1H), 7.03–7.00 (m, 1H), 3.49 (q, *J* = 7.1 Hz, 2H), 3.39 (q, *J* = 7.1 Hz, 2H), 1.28 (t, *J* = 7.1 Hz, 3H), 1.18 (t, *J* = 7.1 Hz, 3H). ^13^C NMR (75.4 MHz, CDCl_3_) δ (ppm): 162.6 (d, *J* = 254.6 Hz, C), 153.4 (d, *J* = 5.6 Hz, C), 152.7 (C), 134.5 (d, *J* = 12.6 Hz, C), 131.6 (2 × CH), 129.0 (CH), 128.4 (2 × CH), 122.7 (C), 119.5 (d, *J* = 3.7 Hz, CH), 113.4 (d, *J* = 24.7 Hz, CH), 106.4 (d, *J* = 18.1 Hz, C), 99.3 (d, *J* = 3.5 Hz, C), 42.63 (CH_2_), 42.3 (CH_2_), 14.2 (CH_3_), 13.4 (CH_3_). EI-LRMS *m/z* (%): 347 (M^+^ + 2, 5), 345 (M^+^, 15), 181 (30), 100 (100), 72 (45). EI-HRMS was calculated for C_19_H_17_ClFNO_2_ [M]^+^ 345.0932, and found 345.0936.

*O-3-Chloro-2-(phenylethynyl)phenyl N,N-diethylcarbamate* (**2da**): The reaction of *O*-3-chlorophenyl *N,N*-diethylcarbamate (**1d**) (227 mg, 1 mmol), following the general procedure, yielded the product as a yellow oil (88% yield); *R*_f_ = 0.27 (hexane/EtOAc, 5:1). ^1^H NMR (300 MHz, CDCl_3_) δ (ppm): 7.60–7.53 (m, 2H), 7.46–7.28 (m, 5H), 7.23–7.16 (m, 1H), 3.56 (q, *J* = 7.0 Hz, 2H), 3.43 (q, *J* = 7.0 Hz, 2H), 1.33 (t, *J* = 7.0 Hz, 3H), 1.21 (t, *J* = 7.0 Hz, 3H). ^13^C NMR (75.4 MHz, CDCl_3_) δ (ppm): 153.1 (C), 152.9 (C), 136.4 (C), 131.3 (2 × CH), 128.8 (CH), 128.6 (CH), 128.2 (2 × CH), 125.8 (CH), 122.6 (C), 120.9 (CH), 117.9 (C), 98.6 (C), 81.8 (C), 42.2 (CH_2_), 41.9 (CH_2_), 14.0 (CH_3_), 13.1 (CH_3_). EI-LRMS *m/z* (%): 329 (M^+^ + 2, 4), 327 (M^+^, 12), 100 (100), 72 (60). ESI-HRMS was calculated for C_19_H_19_ClNO_2_ [M + H]^+^ 328.1099, and found 328.1105.

*O-3,6-Dichloro-2-(phenylethynyl)phenyl N,N-diethylcarbamate* (**2ea**): The reaction of *O*-3,6-dichlorophenyl *N,N*-diethylcarbamate (**1e**) (262 mg, 1 mmol), following the general procedure, yielded the product as a colorless solid (70% yield); mp = 119–121 °C; *R*_f_ = 0.32 (hexane/EtOAc, 10:1). ^1^H NMR (300 MHz, CDCl_3_) δ (ppm): 7.55–7.52 (m, 2H), 7.39–7.29 (m, 5H), 3.56 (q, *J* = 7.1 Hz, 2H), 3.44 (q, *J* = 7.1 Hz, 2H), 1.36 (t, *J* = 7.1 Hz, 3H), 1.21 (t, *J* = 7.1 Hz, 3H). ^13^C NMR (75.4 MHz, CDCl_3_) δ (ppm): 152.1 (C), 149.6 (C), 135.1 (C), 131.9 (2 × CH), 129.6 (CH), 129.1 (CH), 128.5 (2 × CH), 127.0 (C), 126.9 (CH), 122.7 (C), 120.8 (C), 99.8 (C), 81.5 (C), 42.8 (CH_2_), 42.4 (CH_2_), 14.3 (CH_3_), 13.5 (CH_3_). EI-LRMS *m/z* (%): 363 (M^+^ + 2, 1), 361 (M^+^), 100 (100), 72 (42). ESI-HRMS was calculated for C_19_H_18_Cl_2_NO_2_ [M + H]^+^ 362.0709, and found 362.0716.

*O-3-Fluoro-2-((4-methoxyphenyl)ethynyl)phenyl N,N-diethylcarbamate* (**2ab**): The reaction of *O*-3-fluorophenyl *N,N*-diethylcarbamate (**1a**) (211 mg, 1 mmol), following the general procedure, yielded the product as a colorless oil (71% yield); *R*_f_ = 0.24 (hexane/EtOAc, 5:1). ^1^H NMR (300 MHz, CDCl_3_) δ (ppm): 7.48–7.44 (m, 2H), 7.32–7.25 (m, 1H), 7.07 (dt, *J* = 8.3, 1.0 Hz, 1H), 6.99 (td, *J* = 8.3, 1.0 Hz, 1H), 6.91–6.86 (m, 2H), 3.84 (s, 3H), 3.53 (q, *J* = 6.8 Hz, 2H), 3.42 (q, *J* = 7.0 Hz, 2H), 1.31 (t, *J* = 6.8 Hz, 3H), 1.20 (t, *J* = 7.0 Hz, 3H). ^13^C NMR (75.4 MHz, CDCl_3_) δ (ppm): 163.0 (d, *J* = 251.9 Hz, C), 160.1 (C), 153.4 (C), 153.2 (d, *J* = 4.2 Hz, C), 133.2 (2 × CH), 128.9 (d, *J* = 9.6 Hz, CH), 118.5 (d, *J* = 3.3 Hz, CH), 115.2 (C), 114.1 (2 × CH), 112.3 (d, *J* = 21.1 Hz, CH), 107.8 (d, *J* = 18.0 Hz, C), 98.7 (C), 77.2 (C), 55.4 (CH_3_), 42.5 (CH_2_), 42.2 (CH_2_), 14.3 (CH_3_), 13.5 (CH_3_). EI-LRMS *m/z* (%): 341 (M^+^, 8), 100 (100), 72 (84). ESI-HRMS was calculated for C_20_H_21_FNO_3_ [M + H]^+^ 342.1500, and found 342.1504.

*O-2-((4-Chlorophenyl)ethynyl)-3-fluorophenyl N,N-diethylcarbamate* (**2ac**): The reaction of *O*-3-fluorophenyl *N,N*-diethylcarbamate (**1a**) (211 mg, 1 mmol), following the general procedure, yielded the product as a colorless solid (68% yield); mp = 60–62 °C; *R*_f_ = 0.14 (hexane/EtOAc, 10:1). ^1^H NMR (300 MHz, CDCl_3_) δ (ppm): 7.46–7.43 (m, 2H), 7.36–7.30 (m, 3H), 7.08 (d, *J* = 8.3 Hz, 1H), 7.00 (td, *J* = 8.3, 0.9 Hz, 1H), 3.52 (q, *J* = 7.0 Hz, 2H), 3.42 (q, *J* = 7.0 Hz, 2H), 1.30 (t, *J* = 7.0 Hz, 3H), 1.20 (t, *J* = 7.0 Hz, 3H). ^13^C NMR (75.4 MHz, CDCl_3_) δ (ppm): 163.1 (d, *J* = 252.7 Hz, C), 153.36 (d, *J* = 4.1 Hz, C), 153.2 (C), 134.9 (C), 132.9 (2 × CH), 129.6 (d, *J* = 9.7 Hz, CH), 128.8 (2 × CH), 121.5 (C), 118.5 (d, *J* = 3.3 Hz, CH), 112.4 (d, *J* = 21.0 Hz, CH), 107.1 (d, *J* = 18.0 Hz, C), 97.3 (C), 79.6 (C), 42.6 (CH_2_), 42.2 (CH_2_), 14.3 (CH_3_), 13.4 (CH_3_). EI-LRMS *m/z* (%): 345 (M^+^, 6), 100 (100), 72 (62). ESI-HRMS was calculated for C_19_H_18_Cl_F_NO_2_ [M + H]^+^ 346.1005, and found 346.1007.

*O-2-((4-Chlorophenyl)ethynyl)-3-chlorophenyl N,N-diethylcarbamate* (**2dc**): The reaction of *O*-3-chlorophenyl *N,N*-diethylcarbamate (**1d**) (227 mg, 1 mmol), following the general procedure, yielded the product as a colorless solid (79% yield); mp = 80–82 ^°^C; *R*_f_ = 0.19 (hexane/EtOAc, 10:1). ^1^H NMR (300 MHz, CDCl_3_) δ (ppm): 7.47–7.44 (m, 2H), 7.36–7.25 (m, 4H), 7.17 (dd, *J* = 7.3, 2.1 Hz, 1H), 3.52 (q, *J* = 7.1 Hz, 2H), 3.42 (q, *J* = 7.1 Hz, 2H), 1.30 (t, *J* = 7.1 Hz, 3H), 1.20 (t, *J* = 7.1 Hz, 3H). ^13^C NMR (75.4 MHz, CDCl_3_) δ (ppm): 153.3 (C), 136.8 (C), 134.9 (C), 132.9 (2 × CH), 129.3 (CH), 129.0 (C), 128.8 (2 × CH), 126.3 (CH), 121.5 (C), 121.2 (CH), 117.9 (C), 97.6 (C), 83.0 (C), 42.6 (CH_2_), 42.2 (CH_2_), 14.3 (CH_3_), 13.5 (CH_3_). EI-LRMS *m/z* (%): 363 (M^+^ + 2, 4), 361 (M^+^, 2), 100 (100), 72 (50). ESI-HRMS was calculated for C_19_H_18_Cl_2_NO_2_ [M + H]^+^ 362.0709, and found 362.0715.

#### 3.2.2. Synthesis of 2-Arylbenzo[b]furans **3** and **4**

General procedure: A solution of starting carbamate **1** (1 mmol) in THF (2 mL) at –78 °C under nitrogen atmosphere was treated with a solution of *s*-BuLi (0.85 mL of a 1.4 M solution in cyclohexane, 1.2 mmol). The reaction mixture was allowed to reach –65 °C for 5 min (–70 °C for 4-substituted carbamates **1o-q**) and was stirred at this temperature for 90 min. Then, the corresponding alkynylsulfone (1.3 mmol) was added, and the resulting solution was allowed to warm to room temperature and was then stirred for 30 min. The solvents were removed under reduced pressure and the crude solution was treated with NaOH (2 mmol, 80 mg) in DMA (2 mL) or DMSO (2 mL). The resulting solution was warmed by conventional heating (Method A: DMSO, 140 °C) or under microwave irradiation (Method B: DMA, 160 °C), and stirred at this temperature until the completion of the reaction (see Table 3). The mixture was diluted with EtOAc and NH_4_Cl (aq), and the layers were separated. The aqueous phase was extracted with EtOAc (3 × 10 mL), and the combined organic layers were dried over anhydrous Na_2_SO_4_. The solvents were removed under reduced pressure, and the residue was purified by silica gel column chromatography (hexane/EtOAc), affording the 2-arylbenzo[*b*]furans **3** and **4**.

*4-Fluoro-2-phenylbenzo[b]furan* (**3aa**) [17]: The reaction of *O*-3-fluorophenyl *N,N*-diethylcarbamate (**1a**) (211 mg, 1 mmol), following the general procedure with Method A, yielded the product as a white solid (51% yield); *R*_f_ = 0.16 (hexane/EtOAc, 10:1). ^1^H NMR (300 MHz, CDCl_3_) δ (ppm): 7.96–7.87 (m, 2H), 7.56–7.35 (m, 4H), 7.33–7.25 (m, 1H), 7.15 (d, *J* = 0.8 Hz, 1H), 7.03–6.96 (m, 1H). ^13^C NMR (75.4 MHz, CDCl_3_) δ (ppm): 156.8 (d, *J* = 9.8 Hz, C), 156.1 (C), 156.0 (d, *J* = 249.5 Hz, C), 130.0 (C), 129.0 (CH), 128.9 (2 × CH), 125.1 (2 × CH), 124.7 (d, *J* = 7.6 Hz, CH), 118.5 (d, *J* = 22.0 Hz, C), 108.6 (d, *J* = 18.9 Hz, CH), 107.5 (d, *J* = 4.1 Hz, CH), 97.4 (d, *J* = 1.7 Hz, CH). EI-LRMS *m/z* (%): 212 (M^+^, 60), 193 (100), 116 (42).

*4-Fluoro-2-(4-methoxyphenyl)benzo[b]furan* (**3ab**): The reaction of *O*-3-fluorophenyl *N,N*-diethylcarbamate (**1a**) (211 mg, 1 mmol), following the general procedure with Method B, yielded the product as a colorless solid (62% yield); mp = 99–101 °C; *R*_f_ = 0.33 (hexane/EtOAc, 5:1). ^1^H NMR (300 MHz, CDCl_3_) δ (ppm): 7.84–7.81 (m, 2H), 7.33 (dd, *J* = 8.2, 0.7 Hz, 1H), 7.24–7.19 (m, 1H), 7.03–6.9 (m, 4H), 3.89 (s, 1H). ^13^C NMR (75.4 MHz, CDCl_3_) δ (ppm): 160.4 (C), 156.3 (C), 155.9 (d, *J* = 248.9 Hz, C), 126.7 (2 × CH), 124.2 (d, *J* = 7.4 Hz, CH), 122.9 (C), 118.8 (d, *J* = 21.8 Hz, C), 114.5 (2 × CH), 108.5 (d, *J* = 19.0 Hz, CH), 107.4 (d, *J* = 3.6 Hz, CH), 95.8 (CH), 55.5 (CH_3_), one aromatic C does not appear due to overlapping. EI-LRMS *m/z* (%): 242 (M^+^, 100), 227 (84), 199 (80), 170 (28). ESI-HRMS was calculated for C_15_H_12_FO_2_ [M + H]^+^ 243.0816, and found 243.0819.

*2-(4-Chlorophenyl)-4-fluorobenzo[b]furan* (**3ac**): The reaction of *O*-3-fluorophenyl *N,N*-diethylcarbamate (**1a**) (211 mg, 1 mmol), following the general procedure with Method B, yielded the product as a colorless solid (72% yield); mp = 105–107 °C; *R*_f_ = 0.32 (hexane/EtOAc, 5:1). ^1^H NMR (300 MHz, CDCl_3_) δ (ppm): 7.83–7.78 (m, 2H), 7.47–7.43 (m, 2H), 7.33–7.24 (m, 2H), 7.10 (d, *J* = 0.9 Hz, 1H), 6.96 (ddd, *J* = 9.4, 8.0, 0.9 Hz, 1H). ^13^C NMR (75.4 MHz, CDCl_3_) δ (ppm): 156.8 (d, *J* = 9.7 Hz, C), 156.0 (d, *J* = 250.0 Hz, C), 155.0 (C), 134.9 (C), 129.3 (2 × CH), 128.6 (C), 126.4 (2 × CH), 125.1 (d, *J* = 7.6 Hz, CH), 118.5 (d, *J* = 21.9 Hz, C), 108.7 (d, *J* = 18.9 Hz, CH), 107.5 (d, *J* = 4.1 Hz, CH), 97.9 (d, *J* = 1.2 Hz, CH). EI-LRMS *m/z* (%): 246 (M^+^, 100), 127 (12), 183 (46). APCI-HRMS was calculated for C_14_H_9_ClFO [M + H]^+^ 247.0320, and found 247.0324.

*5-Chloro-4-fluoro-2-phenylbenzo[b]furan* (**3ba**): The reaction of *O*-4-chloro-3-fluorophenyl *N,N*-diethylcarbamate (**1b**) (245 mg, 1 mmol), following the general procedure with Method B, yielded the product as a colorless solid (52% yield); mp = 92–94 °C; *R*_f_ = 0.13 (hexane/EtOAc, 10:1). ^1^H NMR (300 MHz, CDCl_3_) δ (ppm): 7.87–7.84 (m, 2H), 7.49–7.42 (m, 3H), 7.28–7.27 (m, 2H), 7.08 (d, *J* = 0.4 Hz, 1H). ^13^C NMR (75.4 MHz, CDCl_3_) δ (ppm): 157.3 (C), 154.9 (d, *J* = 8.9 Hz, C), 151.0 (d, *J* = 252.1 Hz, C), 129.6 (C), 129. (CH), 129.0 (2 × CH), 125.4 (CH), 125.2 (2 × CH), 119.7 (d, *J* = 21.0 Hz, C), 114.3 (d, *J* = 15.9 Hz, C), 108.0 (d, *J* = 4.4 Hz, CH), 97.3 (d, *J* = 1.6 Hz, CH). EI-LRMS *m/z* (%): 248 (M^+^ + 2, 35), 246 (M^+^, 100), 183 (32), 81 (19). APCI-HRMS was calculated for C_14_H_9_ClFO [M + H]^+^ 247.0320, found 247.0326.

*6-Chloro-4-fluoro-2-phenylbenzo[b]furan* (**3ca**): The reaction of *O*-5-chloro-3-fluorophenyl *N,N*-diethylcarbamate (**1c**) (245 mg, 1 mmol), following the general procedure with Method B, yielded the product as a colorless solid (47% yield); mp = 78–80 °C; *R*_f_ = 0.14 (hexane/EtOAc, 10:1). ^1^H NMR (300 MHz, CDCl_3_) δ (ppm): 7.85–7.82 (m, 2H), 7.50–7.35 (m, 4H), 7.03 (d, *J* = 0.9 Hz, 1H), 6.99 (dd, *J* = 9.2, 1.6 Hz, 1H). ^13^C NMR (75.4 MHz, CDCl_3_) δ (ppm): 156.7 (d, *J* = 1.0 Hz, C), 156.3 (d, *J* = 11.3 Hz, C), 155.1 (d, *J* = 252.7 Hz, C), 130.0 (d, *J* = 10.1 Hz, C), 129.5 (C), 129.3 (CH), 129.0 (2 × CH), 125.1 (2 × CH), 117.4 (d, *J* = 21.8 Hz, C), 110.2 (d, *J* = 22.7 Hz, CH), 108.3 (d, *J* = 4.5 Hz, CH), 97.2 (d, *J* = 2.0 Hz, CH). EI-LRMS *m/z* (%): 248 (M^+^ + 2, 35), 246 (M^+^, 100), 183 (36), 81 (17). APCI-HRMS was calculated for C_14_H_9_ClFO [M + H]^+^ 247.0320, and found 247.0325.

*4-Chloro-2-phenylbenzo[b]furan* (**3da**) [17]: The reaction of *O*-3-chlorophenyl *N,N*-diethylcarbamate (**1d**) (227 mg, 1 mmol), following the general procedure with Method A, yielded the product as a white solid (67% yield); *R*_f_ = 0.18 (hexane/EtOAc, 10:1). ^1^H NMR (300 MHz, CDCl_3_) δ (ppm): 7.92–7.89 (m, 2H), 7.52–7.41 (m, 4H), 7.27–7.23 (m, 2H), 7.14 (d, *J* = 0.9 Hz, 1H). ^13^C NMR (75.4 MHz, CDCl_3_) δ (ppm): 156.7 (C), 155.2 (C), 130.0 (C), 129.1 (CH), 129.0 (2 × CH), 128.9 (C, 125.9 (C), 125.2 (2 × CH), 124.9 (CH), 123.0 (CH), 109.8 (CH), 99.9 (CH). EI-LRMS *m/z* (%): 230 (M^+^ + 2, 12), 228 (M^+^, 36), 193 (100), 116 (51).

*4,7-Dichloro-2-phenylbenzo[b]furan* (**3ea**): The reaction of *O*-3,5-dichlorophenyl *N,N*-diethylcarbamate (**1e**) (262 mg, 1 mmol), following the general procedure with Method A, yielded the product as a colorless solid (52% yield); mp = 109–111 °C; *R*_f_ = 0.33 (hexane). ^1^H NMR (300 MHz, CDCl_3_) δ (ppm): 7.95–7.91 (m, 2H), 7.53–7.43 (m, 3H), 7.23 (d, *J* = 8.4 Hz, 1H), 7.18 (d, *J* = 8.4 Hz, 1H), 7.14 (s, 1H). ^13^C NMR (75.4 MHz, CDCl_3_) δ (ppm): 157.5 (C), 150.5 (C), 130.0 (C), 129.5 (CH), 129.3 (C), 129.0 (2 × CH), 125.3 (2 × CH), 124.8 (CH), 124.3 (C), 123.7 (CH), 115.4 (C), 100.4 (CH). EI-LRMS *m/z* (%): 264 (M^+^ + 2, 64), 262 (M^+^, 100), 199 (22), 163 (24). APCI-HRMS was calculated for C_14_H_9_Cl_2_O [M + H]^+^ 263.0025, and found 263.0028.

*4-Chloro-5-fluoro-2-phenylbenzo[b]furan* (**3fa**): The reaction of *O*-3,4-difluorophenyl *N,N*-diethylcarbamate (**1f**) (245 mg, 1 mmol), following the general procedure with Method A, yielded the product as a light yellow solid (57% yield); mp = 103–105 °C; *R*_f_ = 0.13 (hexane/EtOAc, 10:1). ^1^H NMR (300 MHz, CDCl_3_) δ (ppm): 7.89 (d, *J* = 7.8 Hz, 2H), 7.53–7.35 (m, 4H), 7.17–7.05 (m, 4H). ^13^C NMR (75.4 MHz, CDCl_3_) δ (ppm): 158.3 (C), 154.9 (d, *J* = 241.1 Hz, C), 150.6 (C), 129.9 (d, *J* = 2.5 Hz, C), 129.6 (C), 129.4 (CH), 129.0 (2 × CH), 125.2 (2 × CH), 112.4 (d, *J* = 25.5 Hz, CH), 111.6 (d, *J* = 21.3 Hz, C), 110.0 (d, *J* = 8.4 Hz, CH), 100.1 (d, *J* = 4.4 Hz, CH). EI-LRMS *m/z* (%): 248 (M^+^ + 2, 9), 246 (M^+^, 27), 193 (100), 116 (51). APCI-HRMS was calculated for C_14_H_9_ClFO [M + H]^+^ 247.0320, and found 247.0327.

*4,5-Difluoro-2-phenylbenzo[b]furan* (**3ga**): The reaction of *O*-3,4-difluorophenyl *N,N*-diethylcarbamate (**1g**) (229 mg, 1 mmol), following the general procedure with Method B, yielded the product as a colorless solid (53% yield); mp = 86–88 °C; *R*_f_ = 0.15 (hexane/EtOAc, 10:1). ^1^H NMR (300 MHz, CDCl_3_) δ (ppm): 7.91–7.79 (m, 2H), 7.51–7.36 (m, 3H), 7.27– 7.19 (m, 1H), 7.15–7.02 (m, 2H). ^13^C NMR (75.4 MHz, CDCl_3_) δ (ppm): 157.6 (C), 151.7 (d, *J* = 7.9 Hz, C), 146.5 (dd, *J* = 238.8, 11.1 Hz, C), 142.8 (dd, *J* = 251.4, 15.3 Hz, C), 129.6 (C), 129.4 (CH), 129.0 (2 × CH), 125.2 (2 × CH), 120.0 (dd, *J* = 17.6, 2.4 Hz), 113.0 (d, *J* = 21.8 Hz, CH), 106.8 (dd, *J* = 7.4, 4.5 Hz, CH), 97.8 (dd, *J* = 4.6, 1.6 Hz, CH). EI-LRMS *m/z* (%): 230 (M^+^, 31), 193 (100), 116 (51). APCI-HRMS was calculated for C_14_H_9_F_2_O [M + H]^+^ 231.0616, and found 231.0621.

*4,5-Dichloro-2-phenylbenzo[b]furan* (**3ha**): The reaction of *O*-3,4-dichlorophenyl *N,N*-diethylcarbamate (**1h**) (262 mg, 1 mmol), following the general procedure with Method A, yielded the product as a colorless solid (70% yield); mp = 114–116 °C; *R*_f_ = 0.25 (hexane/EtOAc, 5:1). ^1^H NMR (300 MHz, CDCl_3_) δ (ppm): 7.90–7.79 (m, 2H), 7.52–7.32 (m, 5H), 7.09–7.05 (m, 1H). ^13^C NMR (75.4 MHz, CDCl_3_) δ (ppm): 157.7 (C), 153.0 (C), 130.2 (C), 129.5 (C), 129.4 (CH), 128.9 (2 × CH), 126.7 (C), 125.4 (CH), 125.2 (2 × CH), 123.7 (C), 110.5 (CH), 100.2 (CH). EI-LRMS *m/z* (%): 265 (M ^+^ + 4, 3), 263 (M^+^ + 2, 9), 261 ( 27), 211 (41), 193 (100). APCI-HRMS was calculated for C_14_H_9_Cl_2_O [M + H]^+^ 263.0025, and found 263.0032.

*4,5-Dichloro-2-(4-methoxyphenyl)benzo[b]furan* (**3hb**): The reaction of *O*-3,4-dichlorophenyl *N,N*-diethylcarbamate (**1h**) (262 mg, 1 mmol), following the general procedure with Method B, yielded the product as a colorless solid (65% yield); mp = 127–129 °C; *R*_f_ = 0.35 (hexane/EtOAc, 5:1). ^1^H NMR (300 MHz, CDCl_3_) δ (ppm): 7.81–7.78 (m, 2H), 7.36–7.28 (m, 2H), 7.01–6.98 (m, 2H), 6.94 (d, *J* = 0.6 Hz, 1H), 3.89 (s, 3H). ^13^C NMR (75.4 MHz, CDCl_3_) δ (ppm): 160.7 (C), 158.1 (C), 152.9 (C), 130.6 (C), 126.8 (2 × CH), 126.7 (C), 125.0 (CH), 123.4 (C), 122.4 (C), 114.5 (2 × CH), 110.4 (CH), 98.6 (CH), 55.5 (CH_3_). EI-LRMS *m/z* (%): 294 (M^+^ + 2, 80), 292 (M^+^, 100), 278 (75), 249 (38). ESI-HRMS was calculated for C_15_H_11_Cl_2_O_2_ [M + H]^+^ 293.0131, and found 293.0131.

*4,6-Dichloro-2-phenylbenzo[b]furan* (**3ia**): The reaction of *O*-3,5-dichlorophenyl *N,N*-diethylcarbamate (**1i**) (262 mg, 1 mmol), following the general procedure with Method A yielded the product as a colorless solid (46% yield); mp = 92–94 °C; *R*_f_ = 0.11 (hexane/EtOAc, 10:1). ^1^H NMR (300 MHz, CDCl_3_) δ (ppm): 7.88–7.85 (m, 2H), 7.51–7.42 (m, 4H), 7.27 (d, *J* = 1.6 Hz, 1H), 7.05 (d, *J* = 0.9 Hz, 1H). ^13^C NMR (75.4 MHz, CDCl_3_) δ (ppm): 157.4 (C), 154.8 (C), 130.1 (C), 129.6 (C), 129.4 (CH), 129.1 (2 × CH), 127.7 (C), 126.2 (C), 125.2 (2 × CH), 123.6 (CH), 110.6 (CH), 99.7 (CH) EI-LRMS *m/z* (%): 265 (M^+^+4, 6), 263 (M^+^ + 2, 18), 261 (M^+^, 54), 211 (42), 193 (100). APCI-HRMS was calculated for C_14_H_9_Cl_2_O [M + H]^+^ 263.0025, and found 263.0027.

*4,6-Dichloro-2-(4-chlorophenyl)benzo[b]furan* (**3ic**): The reaction of *O*-3,5-dichlorophenyl *N,N*-diethylcarbamate (**1i**) (262 mg, 1 mmol), following the general procedure with Method B yielded the product as a colorless solid (68% yield); mp = 143–145 °C; *R*_f_ = 0.45 (hexane/EtOAc, 5:1). ^1^H NMR (300 MHz, CDCl_3_) δ (ppm): 7.77–7.74 (m, 2H), 7.46–7.42 (m, 3H), 7.26 (d, *J* = 1.6 Hz, 1H), 7.03 (d, *J* = 0.8 Hz, 1H). ^13^C NMR (75.4 MHz, CDCl_3_) δ (ppm): 156.2 (C), 154.8 (C), 135.3 (C), 130.4 (C), 129.3 (2 × CH), 128.0 (C), 127.6 (C), 126.4 (2 × CH), 126.3 (C), 123.7 (CH), 110.6 (CH), 100.1 (CH) EI-LRMS *m/z* (%): 298 (M^+^ + 2, 100), 296 (M^+^, 98), 233 (51), 163 (62). ESI-HRMS could not be recorded.

*5-Bromo-4-fluoro-2-phenylbenzo[b]furan* (**3ja**): The reaction of *O*-4-bromo-3-fluorophenyl *N,N*-diethylcarbamate (**1j**) (290 mg, 1 mmol), following the general procedure with Method B yielded the product as a yellow solid (53% yield); mp = 119–121 °C; *R*_f_ = 0.33 (hexane/EtOAc, 5:1). ^1^H NMR (300 MHz, CDCl_3_) δ (ppm): 7.88–7.85 (m, 2H), 7.51–7.39 (m, 4H), 7.24 (d, *J* = 8.7 Hz, 1H), 7.09 (s, 1H). ^13^C NMR (75.4 MHz, CDCl_3_) δ (ppm): 157.2 (C), 155.6 (d, *J* = 9.0 Hz, C), 152.0 (d, *J* = 250.4 Hz, C), 129.6 (C), 129.4 (CH), 129.1 (2 × CH), 128.0 (CH), 125.3 (2 × CH), 119.8 (d, *J* = 22.2 Hz, C), 108.6 (d, *J* = 4.2 Hz, CH), 101.7 (d, *J* = 19.2 Hz, C), 97.3 (d, *J* = 1.9 Hz, CH). EI-LRMS *m/z* (%): 292 (M^+^ + 2, 100), 290 (M^+^, 93), 183 (17). APCI-HRMS was calculated for C_14_H_9_BrFO [M + H]^+^ 290.9815, and found 290.9824.

*5-Bromo-4-chloro-2-phenylbenzo[b]furan* (**3ka**): The reaction of *O*-4-bromo-3-chlorophenyl *N,N*-diethylcarbamate (**1k**) (306 mg, 1 mmol), following the general procedure with Method A yielded the product as a colorless solid (60% yield); mp = 118–120 °C; *R*_f_ = 0.38 (hexane/EtOAc, 5:1). ^1^H NMR (300 MHz, CDCl_3_) δ (ppm): 7.80 (dd, *J* = 8.1, 1.3 Hz, 2H), 7.49–7.38 (m, 4H), 7.24 (dd, *J* = 8.7, 0.8 Hz, 1H), 7.00 (s, 1H). ^13^C NMR (75.4 MHz, CDCl_3_) δ (ppm): 157.5 (C), 153.5 (C), 130.4 (C), 129.4 (C), 129.3 (CH), 128.9 (2 × CH), 128.4 (CH), 125.7 (C), 125.2 (2 × CH), 116.1 (C), 110.9 (CH), 100.3 (CH). EI-LRMS *m/z* (%): 309 (M^+^ + 4, 11), 307 (M^+^ + 2, 11), 305 (M^+^, 33), 193 (100). APCI-HRMS was calculated for C_14_H_9_BrClO [M + H]^+^ 306.9520, and found 306.9524.

*4,7-Difluoro-2-phenylbenzo[b]furan* (**3la**): The reaction of *O*-2,5-difluorophenyl *N,N*-diethylcarbamate (**1l**) (229 mg, 1 mmol), following the general procedure with Method A yielded the product as a colorless solid (44% yield); mp = 97–99 °C; *R*_f_ = 0.41 (hexane). ^1^H NMR (300 MHz, CDCl_3_) δ (ppm): 7.92–7.89 (m, 2H), 7.52–7.43 (M, 3H), 7.13 (d, *J* = 2.6 Hz, 1H), 6.97 (ddd, *J* = 9.3, 3.9 Hz, 1H), 6.86 (ddd, *J* = 8.8, 3.1 Hz, 1H). ^13^C NMR (75.4 MHz, CDCl_3_) δ (ppm): 157.1 (CH), 151.54 (d, *J* = 245.7 Hz, C), 151.50 (d, *J* = 245.7 Hz, C), 146.2 (d, *J* = 3.9 Hz, C), 143.0 (d, *J* = 3.9 Hz, C), 129.4 (CH), 129.3 (C), 129.0 (2 × CH), 125.3 (2 × CH), 121.2 (dd, *J* = 24.2, 3.1 Hz, C), 110.4 (dd, *J* = 19.1, 8.1 Hz, CH), 108.4 (dd, *J* = 21.9, 6.4 Hz, CH), 97.8 (t, *J* = 1.9 Hz, CH). EI-LRMS *m/z* (%): 230 (M^+^, 100), 201 (42), 181 (8). ESI-HRMS was calculated for C_14_H_9_F_2_O [M + H]^+^ 231.0616, and found 231.0615.

*7-Bromo-4-fluoro-2-phenylbenz[b]ofuran* (**3ma**): The reaction of *O*-2-bromo-5-fluorophenyl *N,N*-diethylcarbamate (**1m**) (290 mg, 1 mmol), following the general procedure with Method A yielded the product as a colorless solid (41% yield); mp = 84–86 °C; *R*_f_ = 0.31 (hexane). ^1^H NMR (300 MHz, CDCl_3_) δ (ppm): 7.94–7.91 (m, 2H), 7.53–7.36 (m, 4H), 7.17 (s, 1H), 6.91–6.85 (m, 1H). ^13^C NMR (75.4 MHz, CDCl_3_) δ (ppm): 157.0 (C), 155.3 (d, *J* = 243.9 Hz, C), 129.5 (CH), 129.5 (C), 129.1 (2 × CH), 127.3 (d, *J* = 7.4 Hz, CH), 125.4 (2 × CH), 125.2 (C), 119.7 (d, *J* = 23.0 Hz, C), 110.2 (d, *J* = 20.6 Hz, CH), 98.8 (d, *J* = 4.4 Hz, C), 98.2 (d, *J* = 1.6 Hz, CH). EI-LRMS *m/z* (%): 291 (M^+^ + 2, 98), 289 (M^+^, 100), 211 (40). ESI-HRMS could not be recorded.

*4-Bromo-2-phenylbenzo[b]furan* (**3na**) [17]: The reaction of *O*-3-bromophenyl *N,N*-diethylcarbamate (**1n**) (272 mg, 1 mmol), following the general procedure with Method A yielded the product as a colorless solid (56% yield); *R*_f_ = 0.18 (hexane/EtOAc, 10:1)). ^1^H NMR (300 MHz, CDCl_3_) δ (ppm): 7.92–7.88 (m, 2H), 7.59–7.46 (m, 3H), 7.45–7.38 m, 2H), 7.17 (t, *J* = 8.1 Hz, 1H), 7.08 (s, 1H). ^13^C NMR (75.4 MHz, CDCl_3_) δ (ppm): 156.6 (C), 154.6 (C), 130.9 (CH), 129.9 (C), 129.1 (2 × CH), 128.9 (CH), 126.1 (2 × CH), 125.2 (C), 113.9 (C), 110.4 (CH), 102.4 (CH), 101.5 (CH). EI-LRMS *m/z* (%): 274 (M^+^ + 2, 96), 272 (M^+^, 100).

*5-Fluoro-2-phenylbenzo[b]furan* (**4oa**) [34]: The reaction of *O*-4-fluorophenyl *N,N*-diethylcarbamate (**1o**) (211 mg, 1 mmol), following the general procedure with Method B yielded the product as a colorless solid (59% yield); *R*_f_ = 0.42 (hexane/EtOAc, 5:1). ^1^H NMR (300 MHz, CDCl_3_) δ (ppm): 7.91–7.87 (m, 2H), 7.53–7.39 (m, 4H), 7.27 (dd, *J* = 8.6, 2.6 Hz, 1H), 7.05 (dt, *J* = 8.6, 2.6 Hz, 1H), 7.00 (d, *J* = 0.8 Hz, 1H). ^13^C NMR (75.4 MHz, CDCl_3_) δ (ppm): 159.5 (d, *J* = 237.9 Hz, C), 157.8 (C), 151.2 (C), 130.2 (C), 130.1 (d, *J* = 11.5 Hz, C), 129.0 (CH), 128.9 (2 × CH), 125.1 (2 × CH), 112.0 (d, *J* = 17.7 Hz, CH), 111.8 (CH), 106.4 (d, *J* = 25.1 Hz, CH), 101.5 (d, *J* = 4.0 Hz, CH). EI-LRMS *m/z* (%): 212 (M^+^, 100), 183 (66), 106 (16).

*5-Chloro-2-phenylbenzo[b]furan* (**4pa**) [35]: The reaction of *O*-4-chlorophenyl *N,N*-diethylcarbamate (**1p**) (227 mg, 1 mmol), following the general procedure with Method B yielded the product as a colorless solid (55% yield); *R*_f_ = 0.15 (hexane/EtOAc, 10:1). ^1^H NMR (300 MHz, CDCl_3_) δ (ppm): 7.87–7.83 (m, 2H), 7.54 (d, *J* = 2.1 Hz, 1H), 7.49–7.38 (m, 4H), 7.24 (dd, *J* = 8.7, 2.1 Hz, 1H), 6.96 (s, 1H). ^13^C NMR (75.4 MHz, CDCl_3_) δ (ppm): 157.5 (C), 153.3 (C), 130.7 (C), 130.1 (C), 129.1 (CH), 129.0 (2 × CH), 128.6 (C), 125.2 (2 × CH), 124.5 (CH), 120.5 (CH), 112.2 (CH), 100.9 (CH). EI-LRMS *m/z* (%): 230 (M^+^ + 2, 12), 228 (M^+^, 36), 193 (100), 116 (51).

*5-Methoxy-2-phenylbenzo[b]furan* (**4qa**) [36]: The reaction of *O*-4-methoxyphenyl *N,N*-diethylcarbamate (**1q**) (223 mg, 1 mmol), following the general procedure with Method B yielded the product as a colorless oil (56% yield); *R*_f_ = 0.15 (hexane/EtOAc, 10:1). ^1^H NMR (300 MHz, CDCl_3_) δ (ppm): 7.90–7.87 (m, 2H), 7.51–7.36 (m, 4H), 7.08 (d, *J* = 2.6 Hz, 1H), 6.99 (d, *J* = 0.9 Hz, 1H), 6.93 (dd, *J* = 8.9, 2.6 Hz, 1H), 3.89 (s, 3H). ^13^C NMR (75.4 MHz, CDCl_3_) δ (ppm): 156.8 (C), 156.2 (C), 150.1 (C), 130.7 (C), 129.9 (C), 128.9 (2 × CH), 128.6 (CH), 125.0 (2 × CH), 113.1 (CH), 111.7 (CH), 103.4 (CH), 101.6 (CH), 56.0 (CH_3_). EI-LRMS *m/z* (%): 224 (M^+^, 100), 153 (44), 76 (41).

#### 3.2.3. Synthesis of *o*-Alkynyl Salicylamides **5**

General Procedure: A solution of starting carbamate **1** (1 mmol) in THF (2 mL) at −78 °C under nitrogen was treated with a solution of *s*-BuLi (0.85 mL of a 1.4 M solution in cyclohexane, 1.2 mmol). The reaction mixture was allowed to reach −65 °C for 5 min and then stirred at this temperature for 90 min. Then, the corresponding alkynyl sulfone (1.3 mmol) was added at −65 °C, and the resulting solution was allowed to reach room temperature and then stirred for 60 min. Then, it was treated with *s*-BuLi (1.42 mL of a 1.4 M solution in cyclohexane, 2 mmol) at −65 °C and stirred at that temperature for 30 min. The mixture was allowed to warm slowly to room temperature and then stirred for an additional 60 min. The reaction mixture was quenched with NH_4_Cl (aq) and diluted with EtOAc. The layers were separated, the aqueous phase was extracted with EtOAc (3 × 10 mL), and the combined organic layers were dried over anhydrous Na_2_SO_4_. The solvents were removed under reduced pressure, and the residue was purified by column chromatography (hexane/EtOAc), affording the *N,N*-diethyl-2-hydroxy-3-(phenylethynyl) benzamides **5**.

*N,N-Diethyl-4-fluoro-2-hydroxy-3-(phenylethynyl) benzamide* (**5aa**): The reaction of *O*-3-fluorophenyl *N,N*-diethylcarbamate (**1a**) (211 mg, 1 mmol), following the general procedure, yielded the product as a yellow oil (39% yield); *R*_f_ = 0.10 (hexane/EtOAc, 5:1). ^1^H NMR (300 MHz, CDCl_3_) δ (ppm): 10.28 (s, 1H), 7.63–7.60 (m, 2H), 7.39–7.37 (m, 3H), 7.26 (dd, *J* = 8.9, 6.3 Hz, 1H), 6.67 (t, *J* = 8.9 Hz, 1H), 3.52 (q, *J* = 7.1 Hz, 4H), 1.29 (t, *J* = 7.1 Hz, 6H). ^13^C NMR (75.4 MHz, CDCl_3_) δ (ppm): 170.3 ©, 164.4 (d, *J* = 255.6 Hz, C), 160.8 (d, *J* = 5.9 Hz, C), 131.94 (2 × CH), 128.8 (CH), 128.5 (2 × CH), 128.2 (CH), 125.2 (C), 122.9 (C), 115.0 (d, *J* = 3.1 Hz, C), 106.1 (d, *J* = 22.0 Hz, CH), 99.7 (C), 77.9 (C), 42.3 (2 × CH_2_), 13.5 (2 × CH_3_). EI-LRMS *m/z* (%): 311 (M^+^ + 2, 25), 310 (M^+^, 22), 239 (100), 183 (51). ESI-HRMS was calculated for C_19_H_19_FNO_2_ [M + H]^+^ 312.1394, and found 312.1400.

*4-Chloro-N,N-diethyl-2-hydroxy-3-(phenylethynyl) benzamide* (**5da**): The reaction of *O*-3-chlorophenyl *N,N*-diethylcarbamate (**1d**) (227 mg, 1 mmol), following the general procedure, yielded the product as a dark yellow oil (79% yield); *R*_f_ = 0.12 (hexane/EtOAc, 5:1). ^1^H NMR (300 MHz, CDCl_3_) δ (ppm): 9.77 (s, 1H), 7.64–7.61 (m, 2H), 7.40–7.36 (m, 3H), 7.15 (d, *J* = 8.4 Hz, 1H), 6.99 (d, *J* = 8.4 Hz, 1H), 3.49 (q, *J* = 7.1 Hz, 4H), 3.25 (t, *J* = 7.1 Hz, 6H). ^13^C NMR (75.4 MHz, CDCl_3_) δ (ppm): 169.7 (C), 158.9 (C), 138.7 (C), 131.9 (2 × CH), 128.9 (CH), 128.4 (2 × CH), 127.5 (CH), 122.8 (C), 119.8 (CH), 118.2 (C), 113.1 (C), 100.1 (C), 81.5 (C), 42.1 (2 × CH_2_), 13.5 (2 × CH_3_). EI-LRMS *m/z* (%): 329 (M^+^ + 2, 1), 327 (M^+^, 3), 292 (32), 58 (100). ESI-HRMS was calculated for C_19_H_19_ClNO_2_ [M + H]^+^ 328.1099, and found 328.1105.

*4-Chloro-N,N-diethyl-2-hydroxy-3-((4-methoxyphenyl)ethynyl)benzamide* (**5db**): The reaction of *O*-3-chlorophenyl *N,N*-diethylcarbamate (**1d**) (227 mg, 1 mmol), following the general procedure, yielded the product as a yellow solid (65% yield); mp = 121–123 °C; *R*_f_ = 0.25 (hexane/EtOAc, 2:1). ^1^H NMR (300 MHz, CDCl_3_) δ (ppm): 9.44 (s, 1H), 7.54 (d, *J* = 9.0 Hz, 2H), 7.13 (d, *J* = 8.4 Hz, 1H), 6.97 (d, *J* = 8.4 Hz, 1H), 6.89 (d, *J* = 9.0 Hz, 2H), 3.83 (s, 3H), 3.47 (q, *J* = 7.1 Hz, 4H), 1.24 (t, *J* = 7.1 Hz, 6H). ^13^C NMR (75.4 MHz, CDCl_3_) δ (ppm): 169.6 (C), 160.2 (C), 158.2 (C), 138.3 (C), 133.4 (2 × CH), 127.2 (CH), 119.9 (CH), 118.4 (C), 114.8 (C), 114.1 (2 × CH), 113.3 (C), 100.4 (C), 80.1 (c), 55.4 (CH_3_), 42.0 (2 × CH_2_), 13.4 (2 × CH_3_). EI-LRMS *m/z* (%): 359 (M^+^ + 2, 40), 357 (M^+^, 53), 285 (100), 151 (50), 42 (49). ESI-HRMS was calculated for C_20_H_21_ClNO_3_ [M + H]^+^ 358.1204, and found 358.1213.

*4,5-Dichloro-N,N-diethyl-2-hydroxy-3-(phenylethynyl)benzamide* (**5ha**): The reaction of *O*-3,4-dichlorophenyl *N,N*-diethylcarbamate (**1h**) (262 mg, 1 mmol), following the general procedure, yielded the product as a yellow solid (52% yield); mp = 155–157 °C; *R*_f_ = 0.18 (hexane/EtOAc, 2:1). ^1^H NMR (300 MHz, CDCl_3_) δ (ppm): 9.34 (s, 1H), 7.63 (dd, *J* = 6.6, 3.1 Hz, 2H), 7.41–7.39 (m, 3H), 7.31 (s, 1H), 3.49 (q, *J* = 7.1 Hz, 4H), 1.27 (t, *J* = 7.1 Hz, 6H). ^13^C NMR (75.4 MHz, CDCl_3_) δ (ppm): 168.3 (C), 156.1 (C), 136.2 (C), 131.9 (2 × CH), 129.1 (CH), 128.5 (2 × CH), 127.9 (CH), 123.4 (C), 122.5 (C), 120.5 (C), 114.8 (C), 101.0 (C), 81.4 (C), 42.0 (2 × CH_2_), 13.4 (2 × CH_3_). EI-LRMS *m/z* (%): 363 (M^+^ + 2, 40), 361 (M^+^, 48), 289 (100), 163 (34). ESI-HRMS was calculated for C_19_H_18_Cl_2_NO_2_ [M + H]^+^ 362.0709, and found 362.0718.

#### 3.2.4. Synthesis of *O*-3-Chloro-6-iodo-2-(arylethynyl)phenyl *N*,*N*-Diethylcarbamates **6**

General Procedure: A solution of *O*-3-chloro-2-iodophenyl *N,N*-diethylcarbamate (**1r**) (317 mg, 0.9 mmol) in THF (2 mL) at −78 °C under nitrogen was treated with a solution of LDA (1.08 mmol). The reaction mixture was stirred at −78 °C for 1 h. Then, the corresponding alkynyl sulfone (1.17 mmol) was added, and the resulting solution was stirred for 2 h at −78 °C. Finally, the resulting solution was warmed to room temperature and stirred overnight. The reaction mixture was quenched with NH_4_Cl (aq), and the aqueous phase was extracted with Et_2_O (3 × 10 mL). The combined organic layers were dried over anhydrous Na_2_SO_4_, and the solvents were removed under reduced pressure. The residue was purified by column chromatography (hexane/EtOAc), affording *O*-3-chloro-6-iodo-2-(arylethynyl)phenyl *N,N*-diethylcarbamates **6**.

*O-3-Chloro-6-iodo-2-(phenylethynyl)phenyl N,N-diethylcarbamate* (**6a**): The use of 2-phenyl *p*-toluenesulfonylacetylene (300 mg), following the general procedure, yielded the product as a colorless solid (60% yield); mp = 87–89 °C; *R*_f_ = 0.27 (hexane/EtOAc, 10:1). ^1^H NMR (300 MHz, CDCl_3_) δ (ppm): 7.69 (dd, *J* = 8.6, 0.9 Hz, 1H), 7.54–7.51 (m, 2H), 7.39–7.36 (m, 3H), 7.08 (dd, *J* = 8.6, 0.9 Hz, 1H), 3.60–3.55 (m, 2H), 3.46–3.42 (m, 2H), 1.39 (t, *J* = 7.1 Hz, 3H), 1.20 (t, *J* = 7.1 Hz, 3H). ^13^C NMR (75.4 MHz, CDCl_3_) δ (ppm): 153.7 (C), 152.0 (C), 138.3 (2 × CH), 137.1 (C), 131.8 (2 × CH), 129.1 (CH), 128.5 (2 × CH), 127.7 (CH), 122.7 (C), 119.9 (C), 99.6 (C), 89.5 (C), 81.7 (C), 42.8 (CH_2_), 42.4 (CH_2_), 14.5 (CH_3_), 13.5 (CH_3_). EI-LRMS *m/z* (%): 455 (M^+^ + 2, 4), 453 (M^+^, 10), 163 (42), 100 (100). ESI-HRMS was calculated for C_19_H_18_ClINO_2_ [M + H]^+^ 454.0065, and found 454.0074.

*O-3-Chloro-6-iodo-2-((4-methoxyphenyl)ethynyl)phenyl N,N-diethylcarbamate* (**6b**): The use of 2-(4-methoxyphenyl) *p*-toluenesulfonylacetylene (334 mg), following the general procedure, yielded the product as a colorless solid (66% yield); mp = 88–90 °C; *R*_f_ = 0.46 (hexane/EtOAc, 3:1). ^1^H NMR (300 MHz, CDCl_3_) δ (ppm): 7.66 (d, *J* = 8.6 Hz, 1H), 7.45 (d, *J* = 8.9 Hz, 2H), 7.06 (d, *J* = 8.6 Hz, 1H), 6.89 (d, *J* = 8.9 Hz, 2H), 3.84 (s, 3H), 3.61–3.52 (m, 2H), 3.49–3.39 (m, 2H), 1.38 (t, *J* = 7.1 Hz, 3H), 1.20 (t, *J* = 7.1 Hz, 3H). ^13^C NMR (75.4 MHz, CDCl_3_) δ (ppm): 160.2 (C), 153.5 (C), 152.0 (C), 137.9 (CH), 136.7 (C), 133.3 (2 × CH), 127.6 (CH), 120.2 (C), 114.7 (C), 114.1 (2 × CH), 99.9 (C), 89.5 (C), 80.5 (C), 55.4 (CH_3_), 42.7 (CH_2_), 42.4 (CH_2_), 14.5 (CH_3_), 13.5 (CH_3_). EI-LRMS *m/z* (%): 485 (M^+^ + 2, 14) 483 (M^+^, 29), 100 (100), 72 (36). ESI-HRMS was calculated for C_20_H_20_ClINO_3_ [M + H]^+^ 484.0171, and found 484.0180.

#### 3.2.5. Synthesis of *O*-3-Chloro-2,6-bis(alkynyl)phenyl *N*,*N*-Diethylcarbamates **7**

General Procedure: To a solution of starting carbamate **6a** (176 mg, 0.5 mmol) in DMF (3 mL) under nitrogen, Et_2_NH (55 mg, 0.75 mmol), the corresponding terminal alkyne (0.6 mmol), PdCl_2_(PPh_3_)_2_ (10.5 mg, 0.025 mmol), and CuI (2.9 mg, 0.015 mmol) were added. The resulting mixture was stirred at room temperature overnight. After completion of the reaction, the solvents were removed under reduced pressure, and the residue was purified by silica gel column chromatography (hexane/EtOAc = 10:1), affording the *O*-3-chloro-2,6-bis(alkynyl)phenyl *N,N*-diethylcarbamates **7**.

*O-3-Chloro-2,6-bis(phenylethynyl)phenyl N,N-diethyl carbamate* (**7aa**): The use of phenylacetylene (61 mg), following the general procedure, yielded the product as a colorless solid (72% yield); mp = 94–96 °C; *R*_f_ = 0.17 (hexane/EtOAc, 10:1). ^1^H NMR (300 MHz, CDCl_3_) δ (ppm): 7.59–7.49 (m, 4H), 7.46 (d, *J* = 8.4 Hz, 1H), 7.40–7.28 (m, 7H), 3.60 (q, *J* = 7.1 Hz, 2H), 3.45 (q, *J* = 7.1 Hz, 2H), 1.38 (t, *J* = 7.1 Hz, 3H), 1.18 (t, *J* = 7.1 Hz, 3H). ^13^C NMR (75.4 MHz, CDCl_3_) δ (ppm): 154.0 (C), 152.7 (C), 136.6 (C), 132.1 (CH), 131.8 (2 × CH), 131.7 (2 × CH), 129.0 (CHs), 128.8 (CH), 128.5 (4 × CH), 126.4 (CH), 123.0 (C), 122.9 (C), 119.6 (C), 117.5 (C), 99.3 (C), 94.8 (C), 83.7 (C), 81.6 (C), 42.7 (CH_2_), 42.4 (CH_2_), 14.4 (CH_3_), 13.5 (CH_3_). EI-LRMS *m/z* (%): 429 (M^+^ + 2, 2), 427 (M^+^, 6), 100 (100), 72 (47). ESI-HRMS was calculated for C_27_H_23_ClNO_2_ [M + H]^+^ 428.1412, and found 428.1418.

*O-3-Chloro-6-((4-chlorophenyl)ethynyl)-2-(phenylethynyl)phenyl N,N-diethylcarbamate* (**7ab**): The use of 1-chloro-4-ethynylbenzene (82 mg), following the general procedure, yielded the product as a dark yellow oil (76% yield); *R*_f_ = 0.20 (hexane/EtOAc, 10:1). ^1^H NMR (300 MHz, CDCl_3_) δ (ppm): 7.57–7.54 (m, 2H), 7.46–7.30 (m, 9H), 3.59 (q, *J* = 7.0 Hz, 2H), 3.45 (q, *J* = 7.0 Hz, 2H), 1.38 (t, *J* = 7.1 Hz, 3H), 1.18 (t, *J* = 7.1 Hz, 3H). ^13^C NMR (75.4 MHz, CDCl_3_) δ (ppm): 154.0 (C), 152.6 (C), 136.8 (C), 134.8 (C), 132.8 (2 × CH), 132.0 (CH), 131.8 (2 × CH), 129.0 (CH), 128.8 (2 × CH), 128.4 (2 × CH), 126.4 (CH), 122.8 (C), 121.4 (C), 119.6 (C), 117.2 (C), 99.4 (C), 93.6 (C), 84.7 (C), 81.5 (C), 42.7 (CH_2_), 42.3 (CH_2_), 14.4 (CH_3_), 13.5 (CH_3_). EI-LRMS *m/z* (%): 463 (M^+^ + 2, 8), 461 (M^+^, 12), 100 (100), 72 (25). ESI-HRMS was calculated for C_27_H_22_Cl_2_NO_2_ [M + H]^+^ 462.1022, and found 462.1031.

*O-3-Chloro-2-(phenylethynyl)-6-(thiophen-3-ylethynyl)phenyl N,N-diethylcarbamate* (**7ac**): The use of 3-ethynylthiophene (65 mg), following the general procedure, yielded the product as a dark yellow oil (70% yield); *R*_f_ = 0.19 (hexane/EtOAc, 10:1). ^1^H NMR (300 MHz, CDCl_3_) δ (ppm): 7.57–7.51 (m, 3H), 7.45–7.30 (m, 6H), 7.17 (d, *J* = 5.0 Hz, 1H), 3.58 (q, *J* = 7.0 Hz, 2H), 3.44 (q, *J* = 7.0 Hz, 2H), 1.38 (t, *J* = 7.0 Hz, 3H), 1.18 (t, *J* = 7.0 Hz, 3H). ^13^C NMR (75.4 MHz, CDCl_3_) δ (ppm): 153.9 (C), 152.6 (C), 136.5 (C), 132.0 (CH), 131.8 (2 × CH), 129.8 (CH), 129.2 (CH), 129.0 (CH), 128.4 (2 × CH), 126.4 (CH), 125.6 (CH), 122.9 (C), 122.0 (C), 119.6 (C), 117.4 (C), 99.3 (C), 90.0 (C), 83.2 (C), 81.6 (C), 42.7 (CH_2_), 42.3 (CH_2_), 14.4 (CH_3_), 13.5 (CH_3_). EI-LRMS *m/z* (%): 435 (M^+^ + 2, 10), 433 (M^+^, 28), 100 (100), 72 (33). ESI-HRMS was calculated for C_25_H_21_ClNO_2_S [M + H]^+^ 434.0976, and found 434.0982.

#### 3.2.6. Synthesis of (4-Chloro-2-Phenylbenzofuran-5-yl)(4-Chlorophenyl) Methanol **8**

A solution of starting carbamate **3ka** (123 mg, 0.4 mmol) in THF (2 mL) at −78 °C under nitrogen was treated with a solution of *n*-BuLi (0.30 mL of a 1.6 M solution in hexane, 0.48 mmol). The reaction mixture was stirred at this temperature for 30 min. Then, 4-chlorobenzaldehyde (67 mg, 0.48 mmol) was added, and the resulting solution was warmed to room temperature. The mixture was quenched with aqueous NH_4_Cl, diluted with EtOAc, and the layers were separated. The aqueous phase was extracted with EtOAc (3 × 10 mL), and the combined organic layers were dried over anhydrous Na_2_SO_4_. The solvents were removed under reduced pressure, and the residue was purified by column chromatography (hexane/EtOAc = 5:1), affording functionalized benzo[*b*]furan derivative **8**.

*(4-Chloro-2-phenylbenzofuran-5-yl)(4-chlorophenyl) methanol* (**8**): Colorless oil (83% yield); *R*_f_ = 0.26 (hexane/EtOAc, 5:1). ^1^H NMR (300 MHz, CDCl_3_) δ (ppm): 7.89–7.63 (m, 2H), 7.48–7.34 (m, 9H), 7.10 (s, 1H), 6.34 (s, 1H), 2.58 (s, 1H). ^13^C NMR (75.4 MHz, CDCl_3_) δ (ppm): 157.2 (C), 154.3 (C), 141.4 (C), 135.3 (C), 133.5 (C), 129.9 (C), 129.3 (C), 129.2 (CH), 129.0 (2 × CH), 128.7 (2 × CH), 128.1 (2 × CH), 127.4 (C), 125.2 (2 × CH), 123.8 (CH), 110.3 (CH), 100.2 (CH), 71.8 (CH). EI-LRMS *m/z* (%): 372 (M^+^+4, 6), 370 (M^+^ + 2, 18), 368 (M^+^, 54), 351 (100), 281 (41). ESI-HRMS was calculated for C_21_H_14_Cl_2_NaO_2_ [M + Na]^+^ 391.0263, and found 391.0267.

#### 3.2.7. Synthesis of 4-Chloro-2,5-Diphenylbenzofuran **9**

A solution of starting carbamate **3ka** (123 mg, 0.4 mmol) in toluene (2 mL) was mixed with acid phenylboronic acid (97 mg, 0.8 mmol), Pd(OAc)_2_ (5 mg, 5 mol%), PPh_3_ (11 mg, 10 mol%), and K_3_PO_4_ (169 mg, 0.8 mmol). The resulting mixture was stirred at 100 °C for 12 h. After completion of the reaction, the solvents were removed under reduced pressure, and the residue was purified by silica gel column chromatography (hexane/EtOAc = 10:1), affording benzo[*b*]furan derivative **9**.

*4-Chloro-2,5-diphenylbenzofuran* (**9**): White solid (81% yield); mp = 116–118 °C; *R*_f_ = 0.18 (hexane/EtOAc, 10:1). ^1^H NMR (300 MHz, CDCl_3_) δ (ppm): 7.95–7.92 (m, 2H), 7.53–7.49 (m, 9H), 7.30 (d, *J* = 8.4 Hz, 1H), 7.20 (d, *J* = 0.9 Hz, 1H). ^13^C NMR (75.4 MHz, CDCl_3_) δ (ppm): 157.2 (C), 154.2 (C), 139.6 (C), 135.4 (C), 130.1 (C), 130.0 (2 × CH), 129.6 (C), 129.2 (CH), 129.0 (2 × CH), 128.2 (2 × CH), 127.5 (CH), 127.3 (CH), 125.3 (2 × CH), 123.9 (C), 109.8 (CH), 100.6 (CH). EI-LRMS *m/z* (%): 306 (M^+^ + 2, 34), 304 (M^+^, 100), 239 (35), 119 (20). ESI-HRMS was calculated for C_20_H_14_ClO [M + H]^+^ 305.0728, and found 305.0739.

## 4. Conclusions

In summary, we have reported a straightforward protocol to synthesize regioselectively functionalized benzo[*b*]furans, particularly challenging those 4-halo-substituted derivatives, in a one-pot transition metal-free process employing readily available starting materials, such as *O*-3-halophenyl *N*,*N*-diethylcarbamates. The process is triggered by an initial C–H bond functionalization step through directed *ortho*-metalation (D*o*M), taking advantage of the extraordinary ability of the carbamate group to act as a directed metalation group. The generated organolithium intermediate was successfully reacted with arylsufonylacetylenes, affording the corresponding *o*-alkynylaryl carbamates, which, upon addition of NaOH and subsequent heating under conventional thermal or microwave irradiation, delivered the desired benzo[*b*]furans. In addition, by applying a halogen dance strategy, closely related *O*-6-alkynyl-5-chloro-2-iodophenyl carbamates were easily obtained from *O*-3-chloro-2-iodophenyl carbamate, expanding the scope of this transformation. Further derivatization allowed the synthesis of *o,o*´-dialkynylaryl carbamates. The carbamate moiety has demonstrated a wide versatility in increasing structural complexity by efficiently developing D*o*M processes, as shown with the synthesis of related 3-alkynylsalicylamides.

## Figures and Tables

**Table 1:**
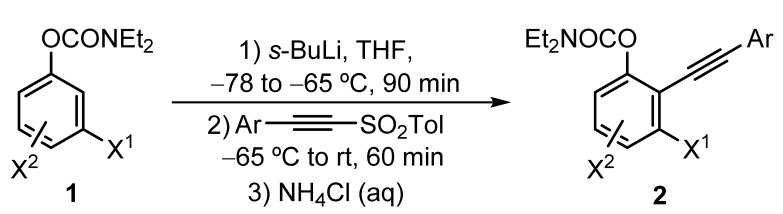
Synthesis of *O*-2-alkynylaryl *N,N*-diethyl carbamates **2** ^a^.

Entry	Starting Carbamate ^b^	X^1^	X^2^	Ar	Product	Yield (%) ^c^
1	**1a**	F	H	Ph	**2aa**	73
2	**1b**	F	4-Cl	Ph	**2ba**	75
3	**1c**	F	5-Cl	Ph	**2ca**	80
4	**1d**	Cl	H	Ph	**2da**	88
5	**1e**	Cl	6-Cl	Ph	**2ea**	70
6	**1a**	F	H	4-MeOC_6_H_4_	**2ab**	71
7	**1a**	F	H	4-ClC_6_H_4_	**2ac**	68
8	**1d**	Cl	H	4-ClC_6_H_4_	**2dc**	79

^a^ Reaction conditions: (1) 1 (1 mmol), s-BuLi (1.2 mmol), THF (2 mL); (2) alkynylsulfone (1.3 mmol). ^b^ Position of the second halogen atom (X^2^) considering that X^1^ is located at the C-3 position. ^c^ Isolated yields of products **2** referred to the corresponding starting carbamate **1**.

**Table 2:**
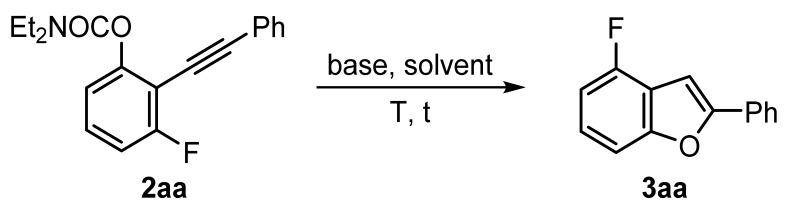
Optimization of the hydrolysis–cyclization reaction of **2aa** ^a^.

Entry	Base ^b^	Solvent	Heating ^c^	T (°C)	t (h)	Conversion (%) ^d^
1 ^e^	Cs_2_CO_3_	EtOH	∆	60	48	0
2	K_2_CO_3_	DMF	∆	150	20	0
4	LiAlH_4_	THF	∆	60	20	0
5 ^f^	Et_3_N	THF	∆	80	20	0
6	NaOH	MeOH	∆	60	48	0
7	KOH	MeOH	∆	60	48	0
8 ^g^	NaOH	EtOH	∆	60	48	100
9 ^g^	NaOH	DMF	∆	140	18	100
10 ^g^	NaOH	DMSO	∆	140	12	100
11 ^g^	NaOH	DMA	μW	160	0.66	>95

^a^ Reaction conditions: **2aa** (0.3 mmol), base (1.2 mmol), solvent (1.2 mL). ^b^ NaOH and KOH were added in powder from freshly grounded pearls. ^c^ ∆: conventional heating; μW: microwave irradiation. ^d^ Determined by ^1^H-NMR analysis of the crude reaction mixture. ^e^ The use of other solvents, such as EtOH, MeCN, or even DMF at 150 °C, resulted in no conversion. ^f^ Carried out in a sealed tube. ^g^ Using KOH, no significant differences were observed.

**Table 3:**
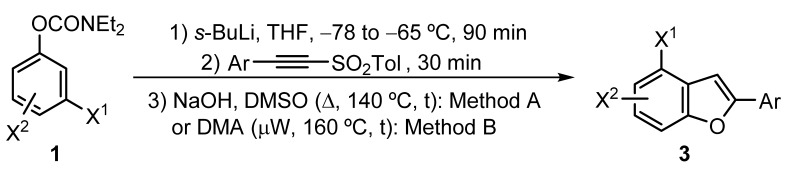
One-pot reaction: Synthesis of 4,*n*-halobenzo[*b*]furans **3** from carbamates **1** ^a^.

Entry	1 ^b^	X^1^	X^2^	Ar	Method	t (h)	Product	Yield (%) ^c^
1	**1a**	F	H	Ph	A	12	**3aa**	51
2	**1a**	F	H	4-MeOC_6_H_4_	B	1	**3ab**	62
3	**1a**	F	H	4-ClC_6_H_4_	B	1	**3ac**	72
5	**1b**	F	4-Cl	Ph	B	1.3	**3ba**	52
6	**1c**	F	5-Cl	Ph	B	1	**3ca**	47
7	**1d**	Cl	H	Ph	A	2	**3da**	67
8	**1e**	Cl	6-Cl	Ph	A	24	**3ea**	52
9	**1f**	Cl	4-F	Ph	A	12	**3fa**	57
10	**1g**	F	4-F	Ph	B	1	**3ga**	53
11	**1h**	Cl	4-Cl	Ph	A	4	**3ha**	70
12	**1h**	Cl	4-Cl	4-MeOC_6_H_4_	B	1	**3hb**	65
13	**1i**	Cl	5-Cl	Ph	A	8	**3ia**	46
14	**1i**	Cl	5-Cl	4-ClC_6_H_4_	B	1	**3ic**	68
15 ^d^	**1j**	F	4-Br	Ph	B	1	**3ja**	53
16 ^d^	**1k**	Cl	4-Br	Ph	A	20	**3ka**	60
17	**1l**	F	6-F	Ph	A	24	**3la**	44
18 ^d^	**1m**	F	6-Br	Ph	A	24	**3ma**	41
19 ^d^	**1n**	Br	H	Ph	A	8	**3na**	56

^a^ Reaction conditions: (1) **1** (1 mmol), *s*-BuLi (1.2 mmol), THF (2 mL); (2) alkynylsulfone (1.3 mmol); (3) NaOH (2 mmol), DMA (160 °C) or DMSO (140 °C) (2 mL). ^b^ Position of the second halogen atom (X^2^) considering that X^1^ is located at the C-3 position. ^c^ Isolated yields of products **3** referred to the corresponding starting carbamate **1**. ^d^ LDA was employed as the base instead of *s*-BuLi.

**Table 4:**
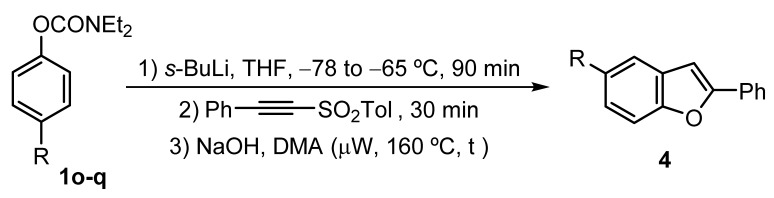
Synthesis of 5-functionalized benzo[*b*]furans **4** from carbamates **1o-q**.

Entry	1	R	t (h)	Product	Yield (%) ^a^
1	**1o**	F	1	**4oa**	59
2	**1p**	Cl	1.5	**4pa**	55
3	**1q**	OMe	1.5	**4qa**	56

^a^ Isolated yield of products **4** referred to as starting carbamates **1**.

## Data Availability

The data presented in this study are available in this article.

## Supplementary Materials

The following supporting information can be downloaded online. Copies of ^1^H-NMR and ^13^C-NMR spectra of all new compounds.

## Sample Availability

Not available.
