# Supplementary material for: Transition Metal-Free Synthesis of Halobenzo[b]furans from O-Aryl Carbamates via o-Lithiation Reactions
Source: Molecules. 2022 Jan 14;27(2):525. doi: 10.3390/molecules27020525 (PMC8778012; doi:10.3390/molecules27020525)

# SUPPLEMENTARY MATERIAL

# Transition Metal-Free Synthesis of Halobenzo[*b*]furans from *O*-Arylcarbamates via *o*-Lithiation Reactions

Claudia Feberero [1], Cintia Virumbrales [1], Carlos Sedano [1], Lorena Renedo [1], Samuel Suárez-Pantiga [1] and Roberto Sanz [1,*]

[1] Área de Química Orgánica, Departamento de Química, Facultad de Ciencias, Universidad de Burgos, Pza. Misael Bañuelos s/n, 09001 Buros, Spain; rsd@ubu.es

* Correspondence: rsd@ubu.es

**Table of Contents**

$^1$H NMR and $^{13}$C NMR spectra of compounds **2-9** ............................................S2-S79

$^1H$ NMR (300 MHz, $CDCl_3$)

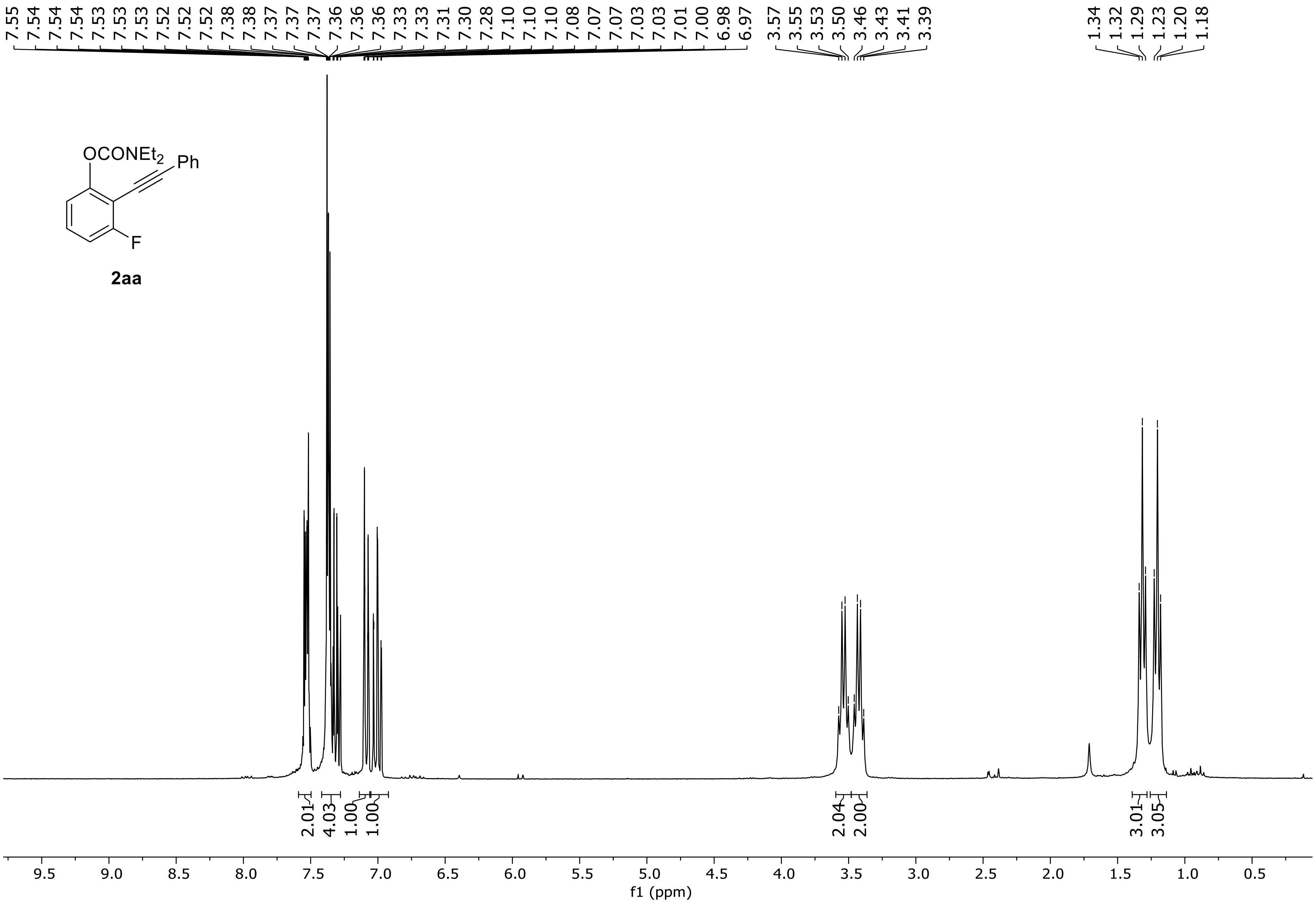

$^{13}C$ NMR (75.4 MHz, $CDCl_3$)

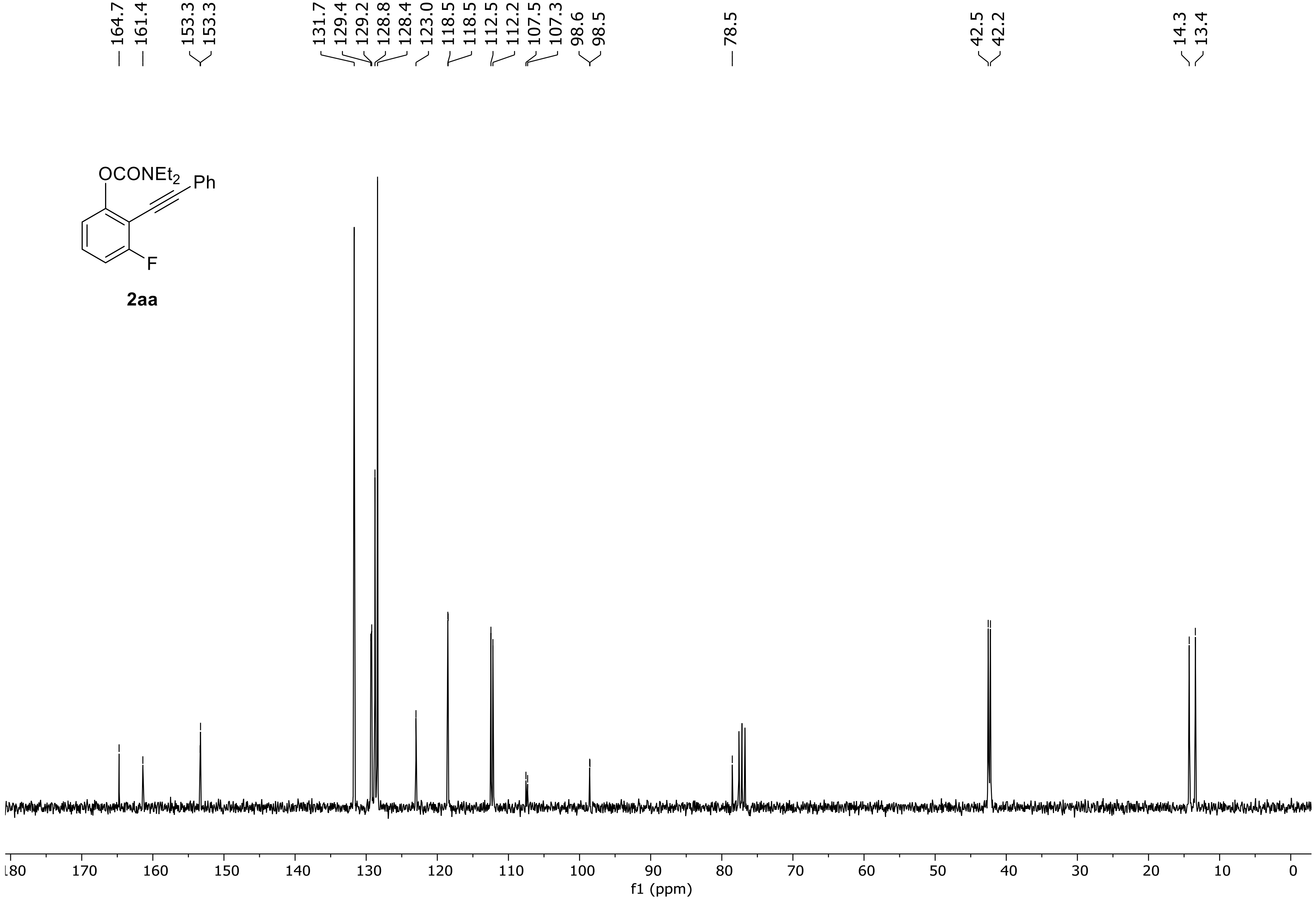


**2aa**

$^{1}H$ NMR (300 MHz, $CDCl_3$)

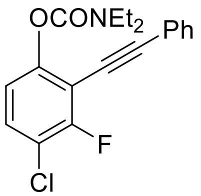


**2ba**

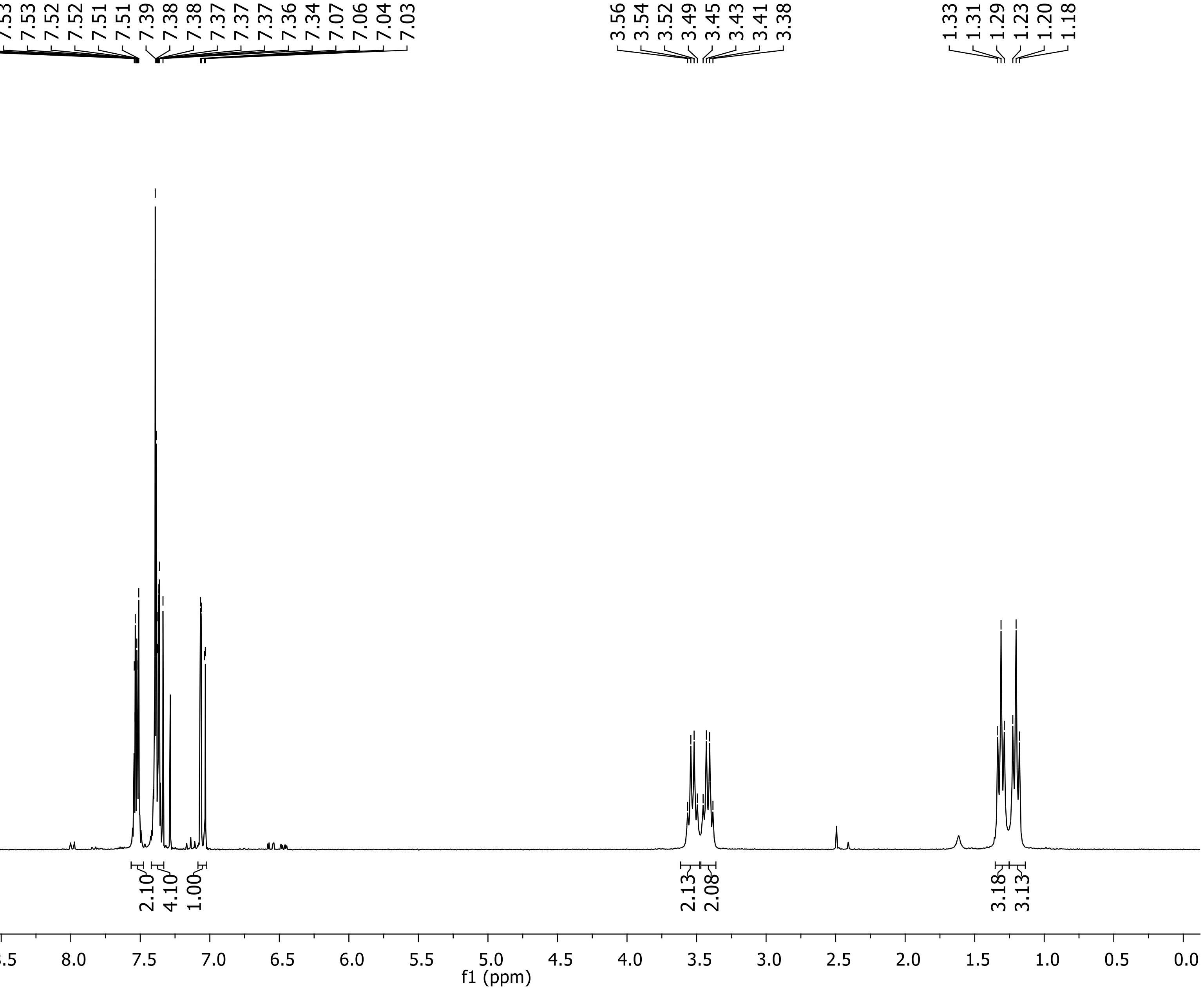

$^{13}C$ NMR (75.4 MHz, $CDCl_3$)

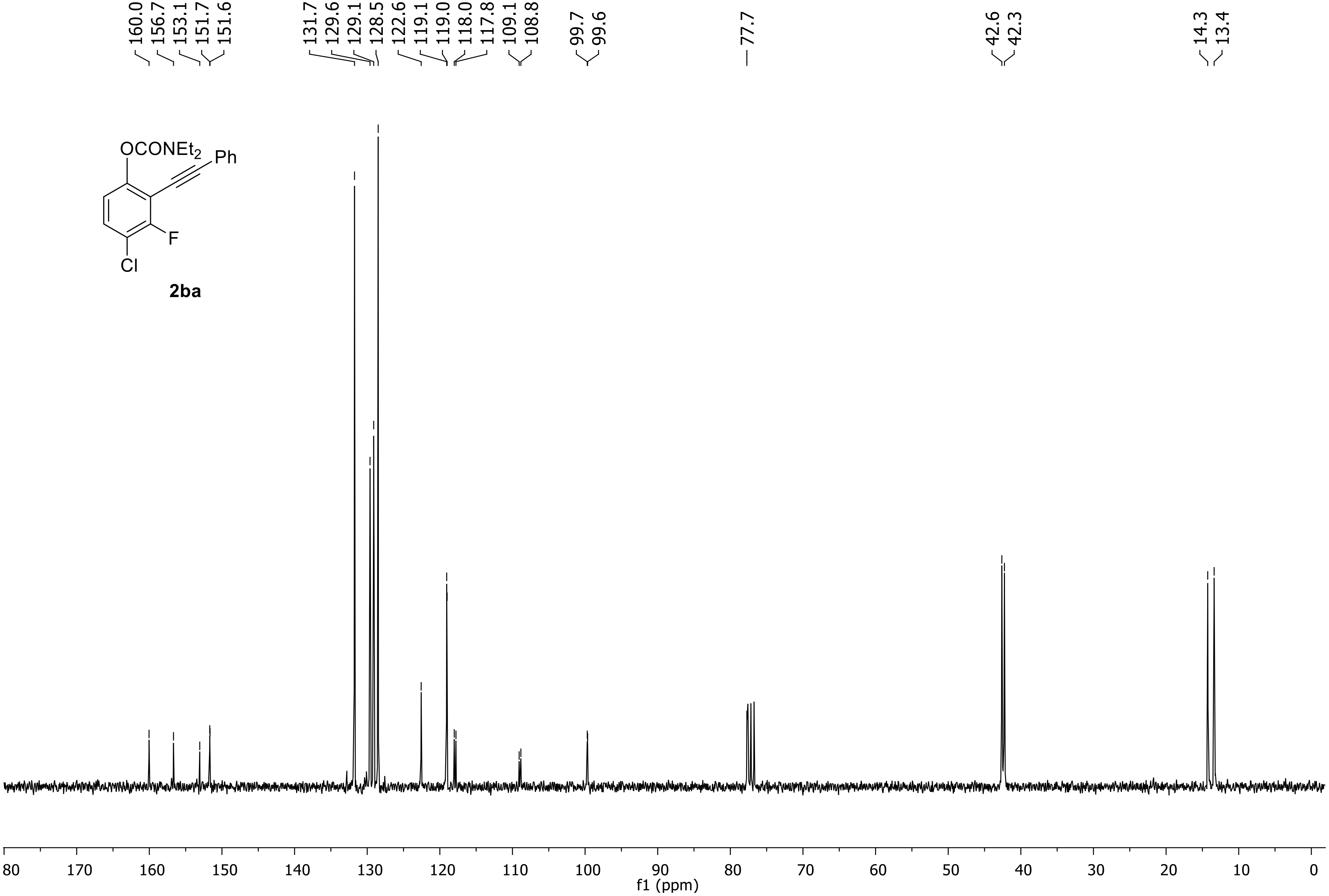

$^{1}H$ NMR (300 MHz, $CDCl_3$)

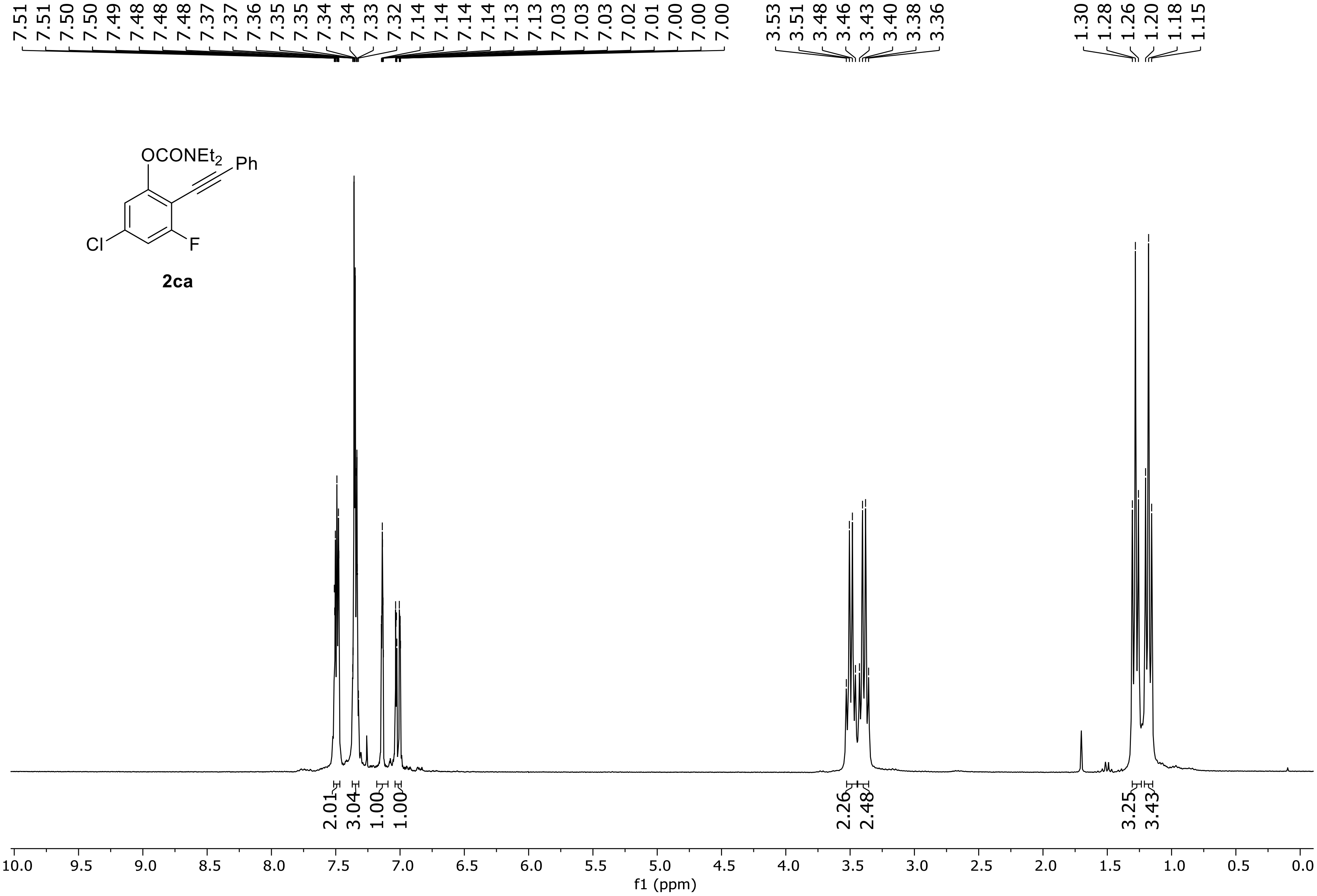


**2ca**

$^{13}C$ NMR (75.4 MHz, $CDCl_3$)

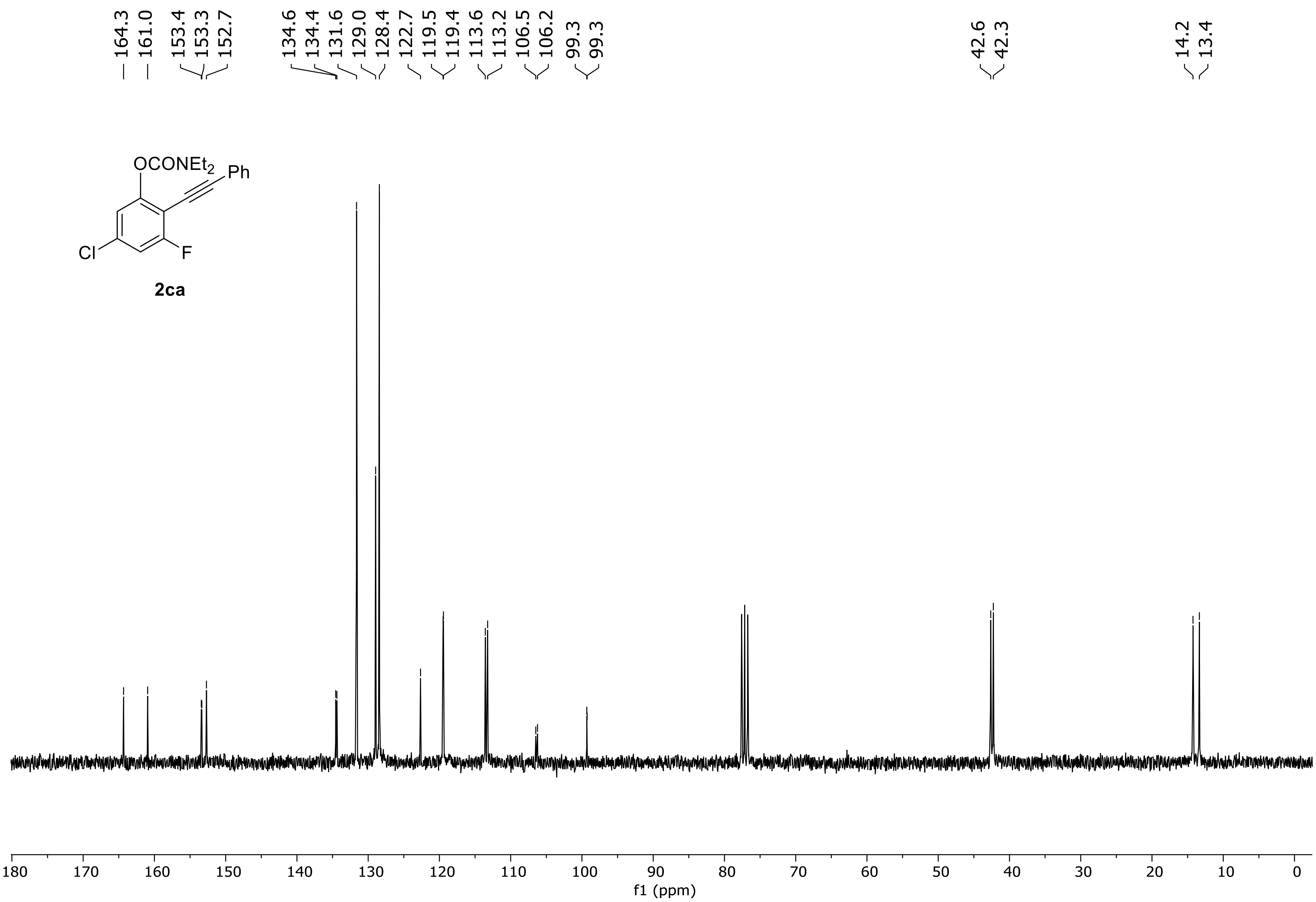


**2ca**

$^{1}$H NMR (300 MHz, $CDCl_3$)

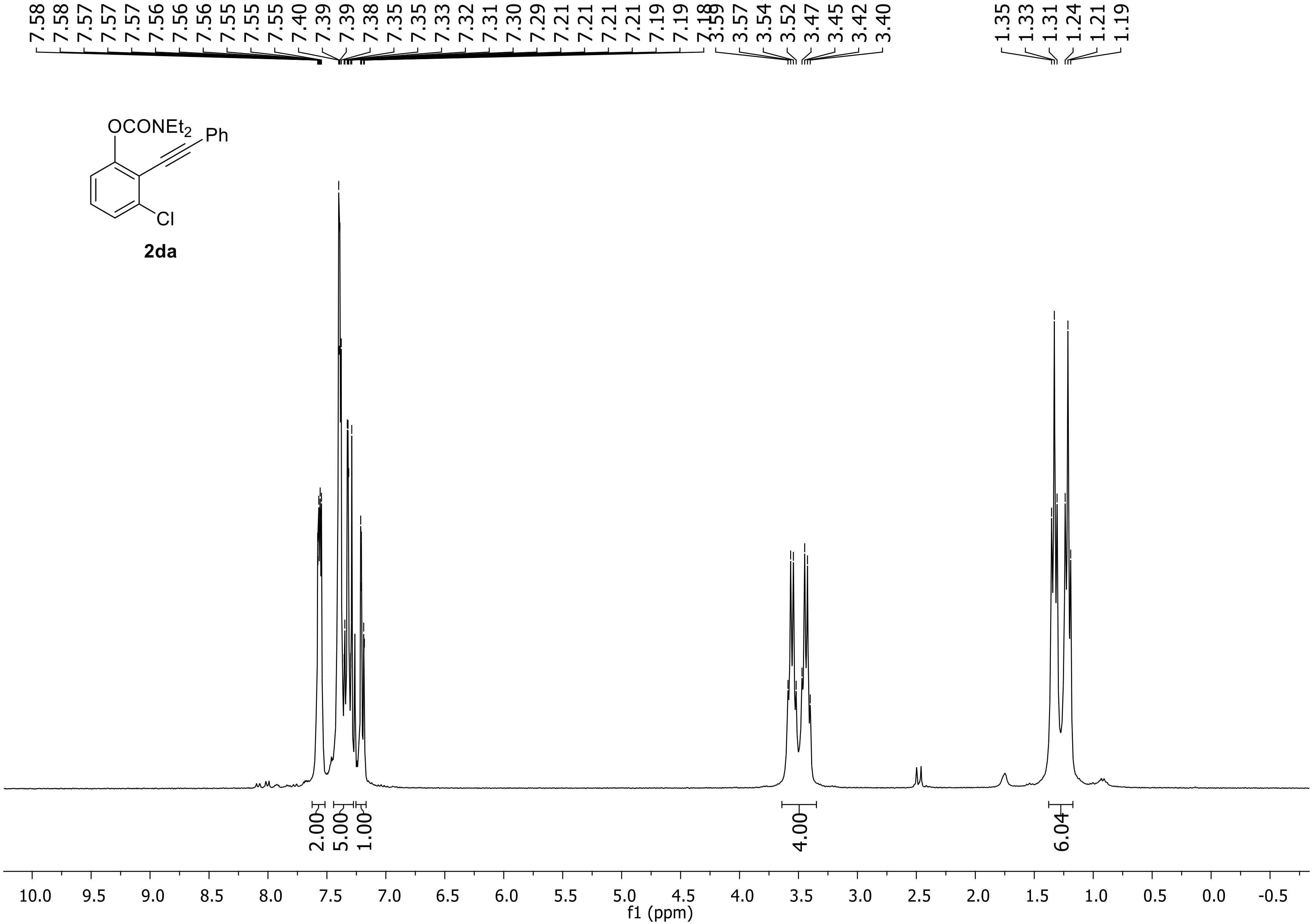

$^{13}C$ NMR (75.4 MHz, $CDCl_3$)

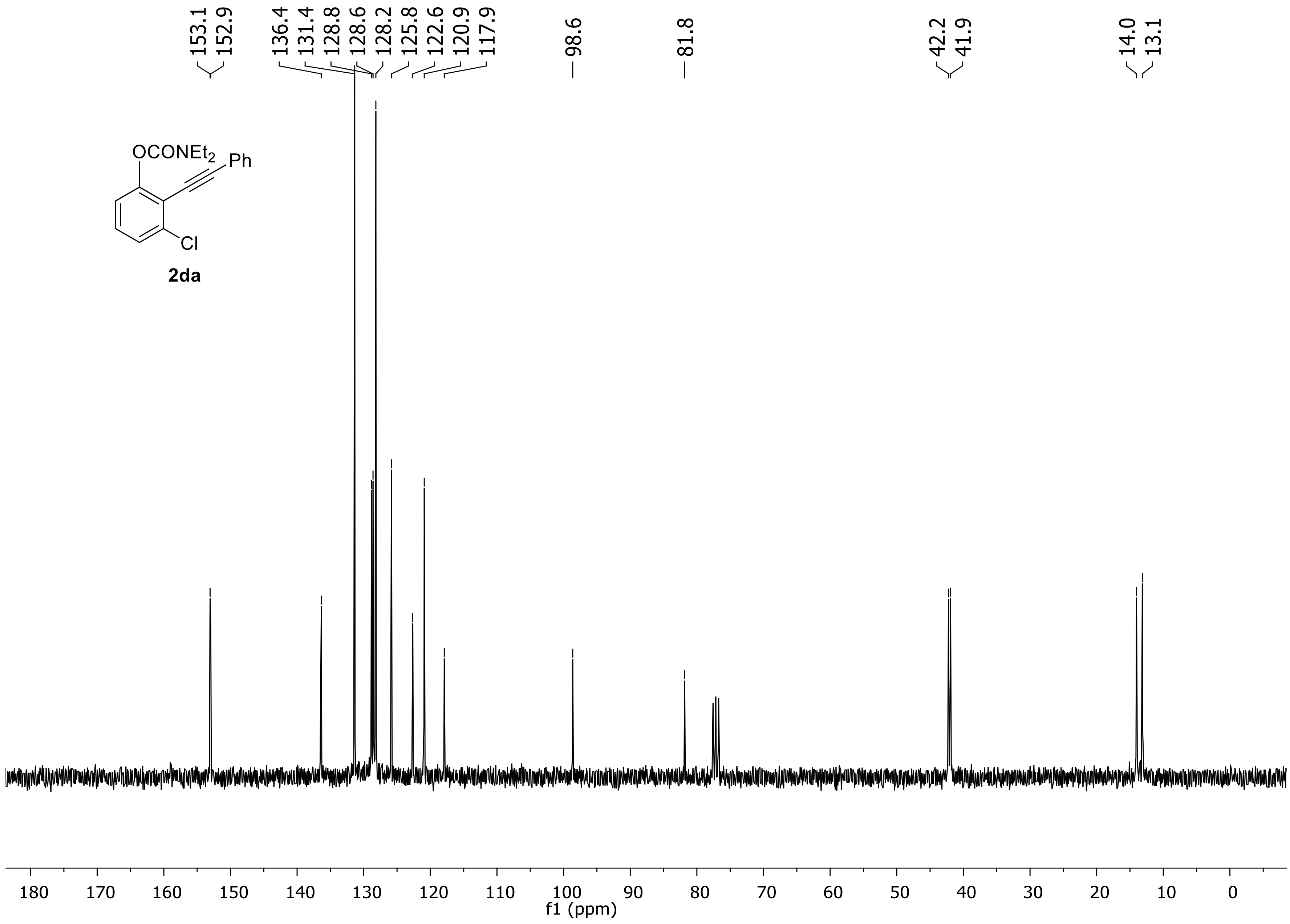

$^1$H NMR (300 MHz, $CDCl_3$)

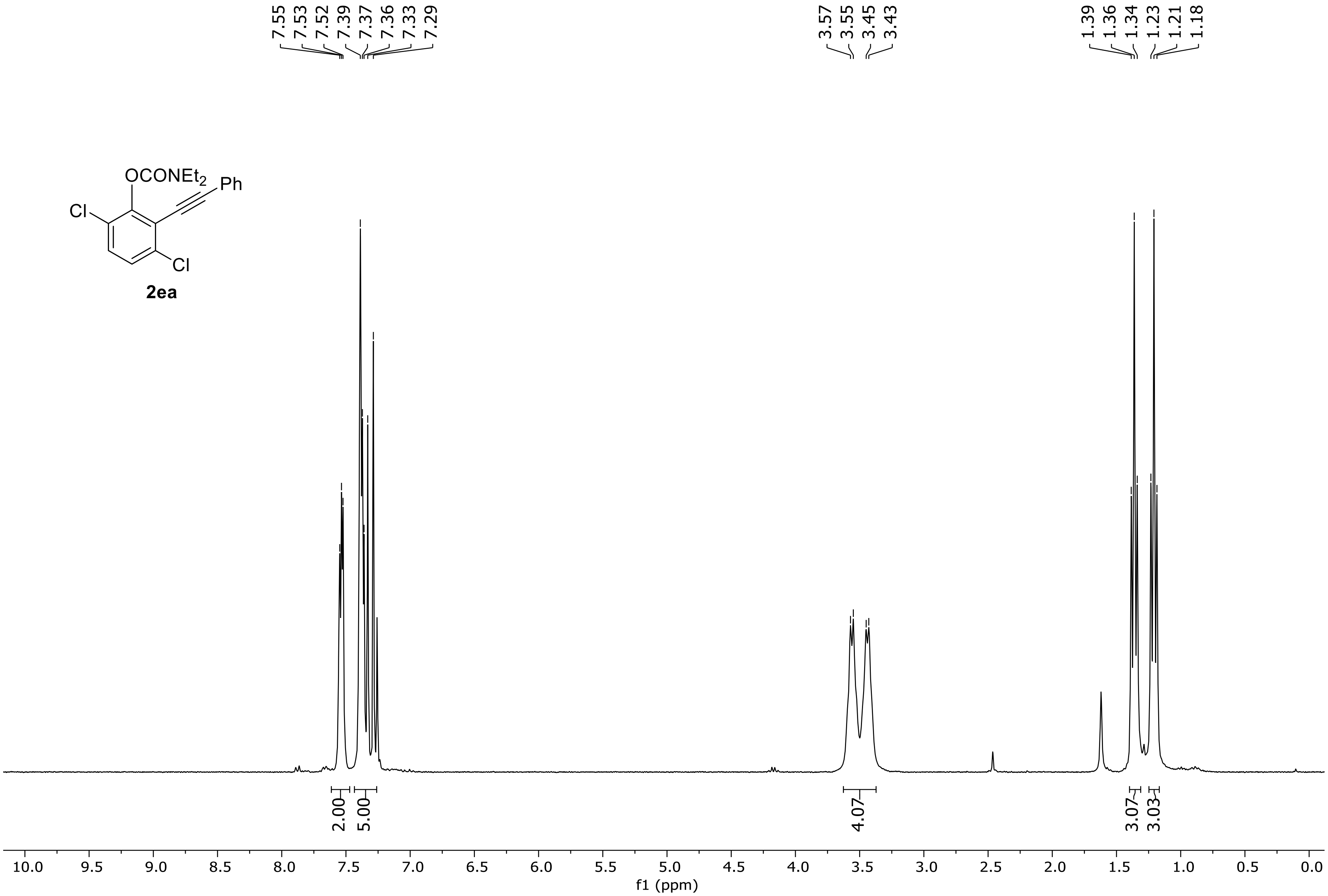

$^{13}$C NMR (75.4 MHz, $CDCl_3$)

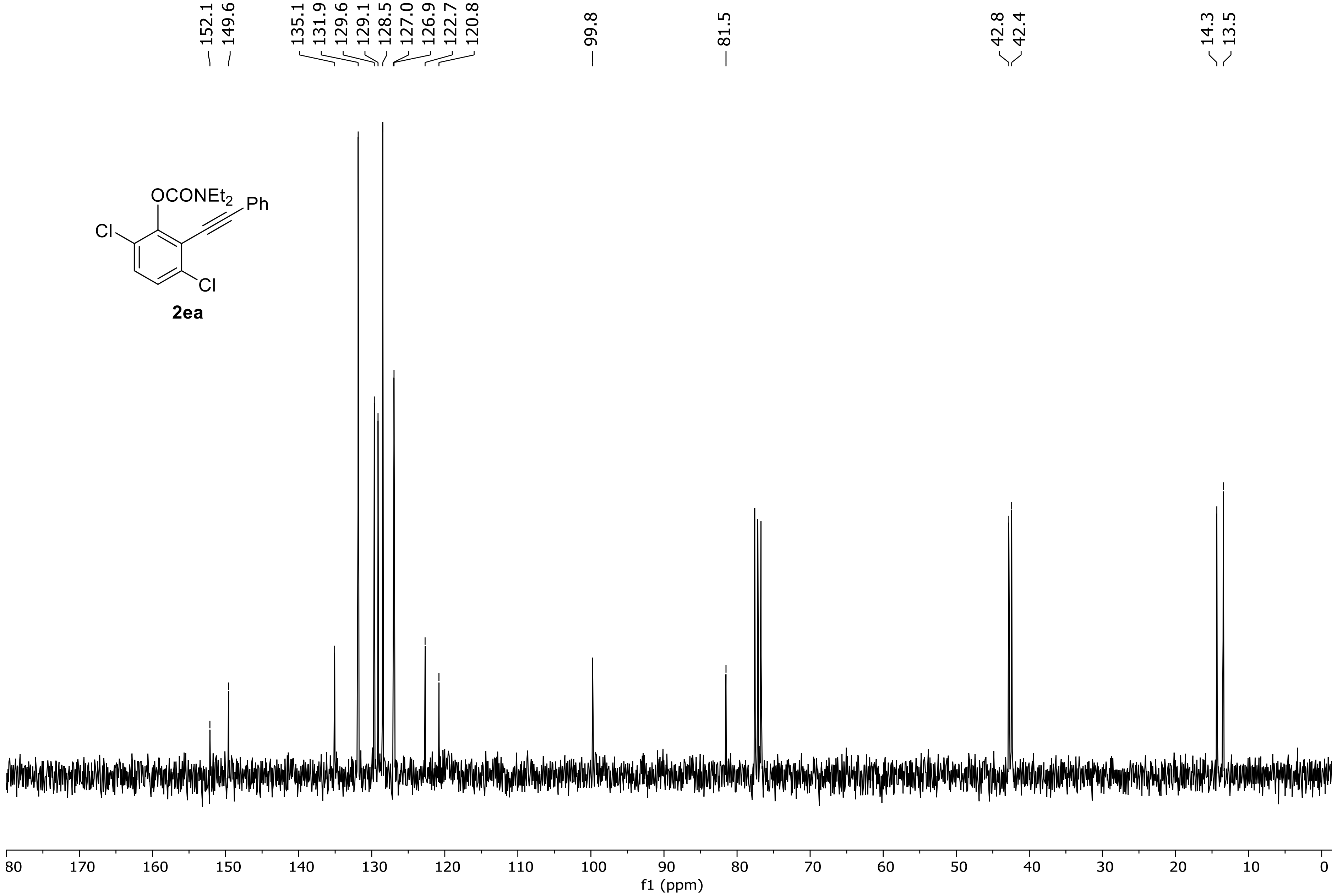

$^1$H NMR (300 MHz, $CDCl_3$)

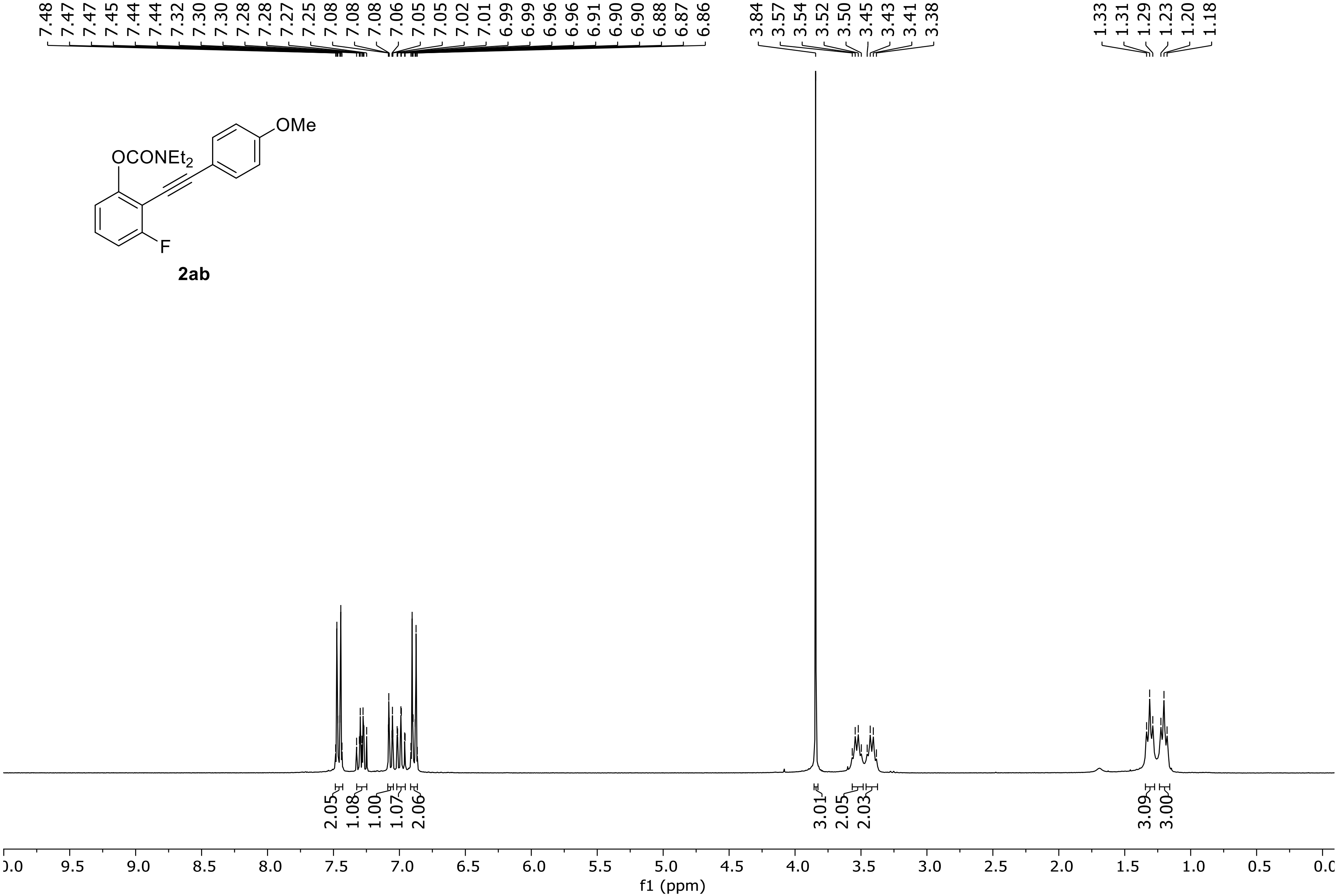

$^{13}C$ NMR (75.4 MHz, $CDCl_3$)

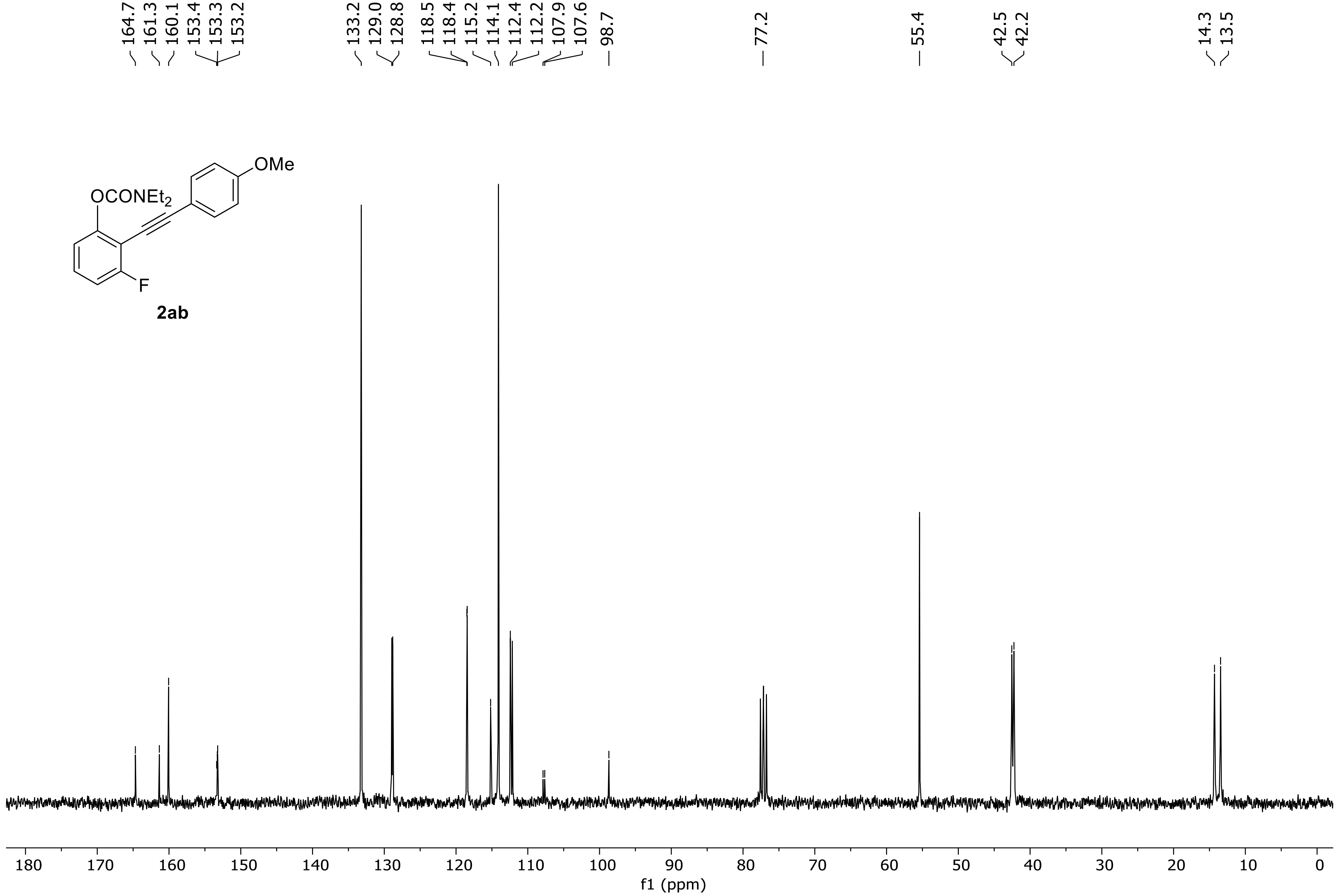

$^1H$ NMR (300 MHz, $CDCl_3$)

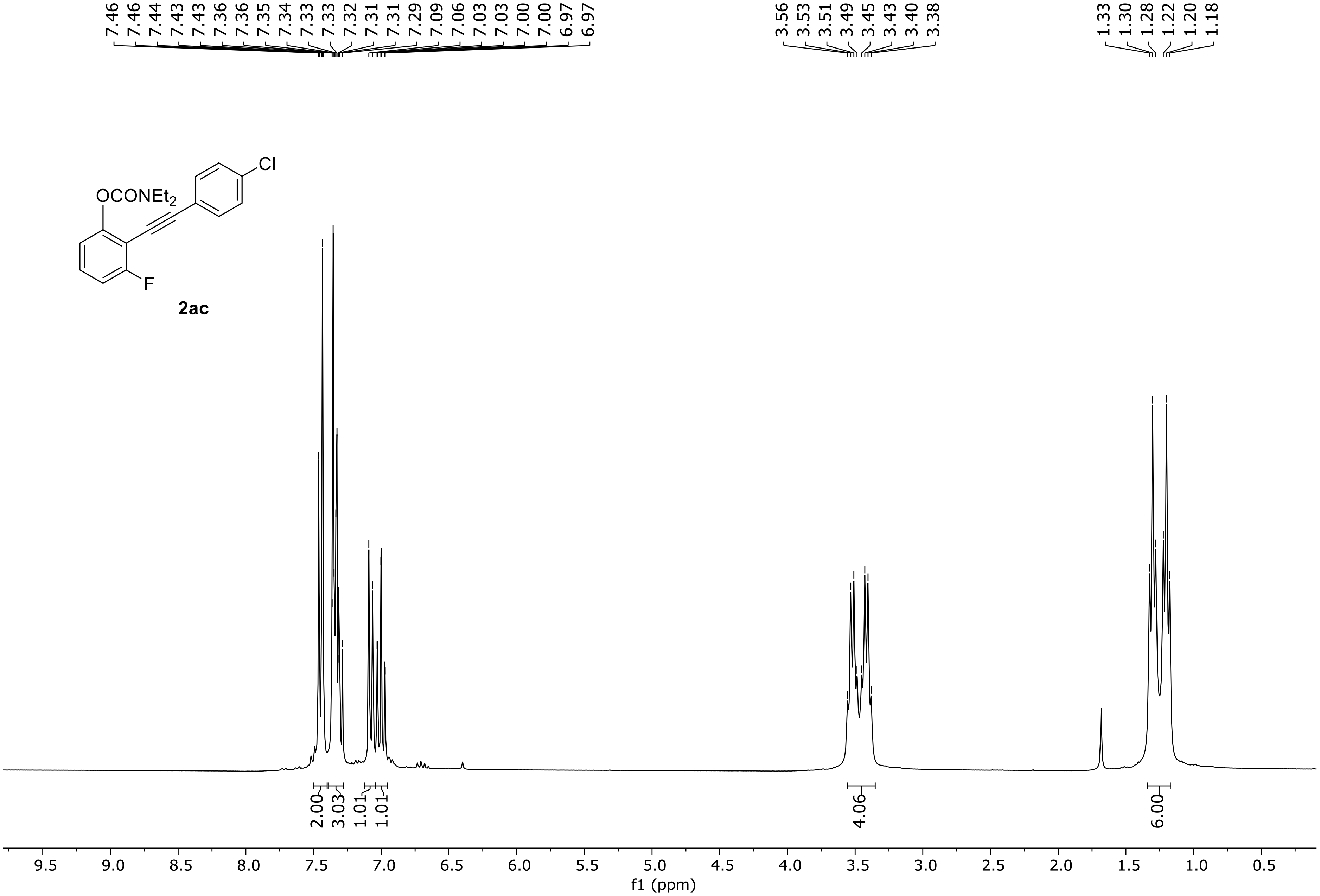

$^{13}C$ NMR (75.4 MHz, $CDCl_3$)

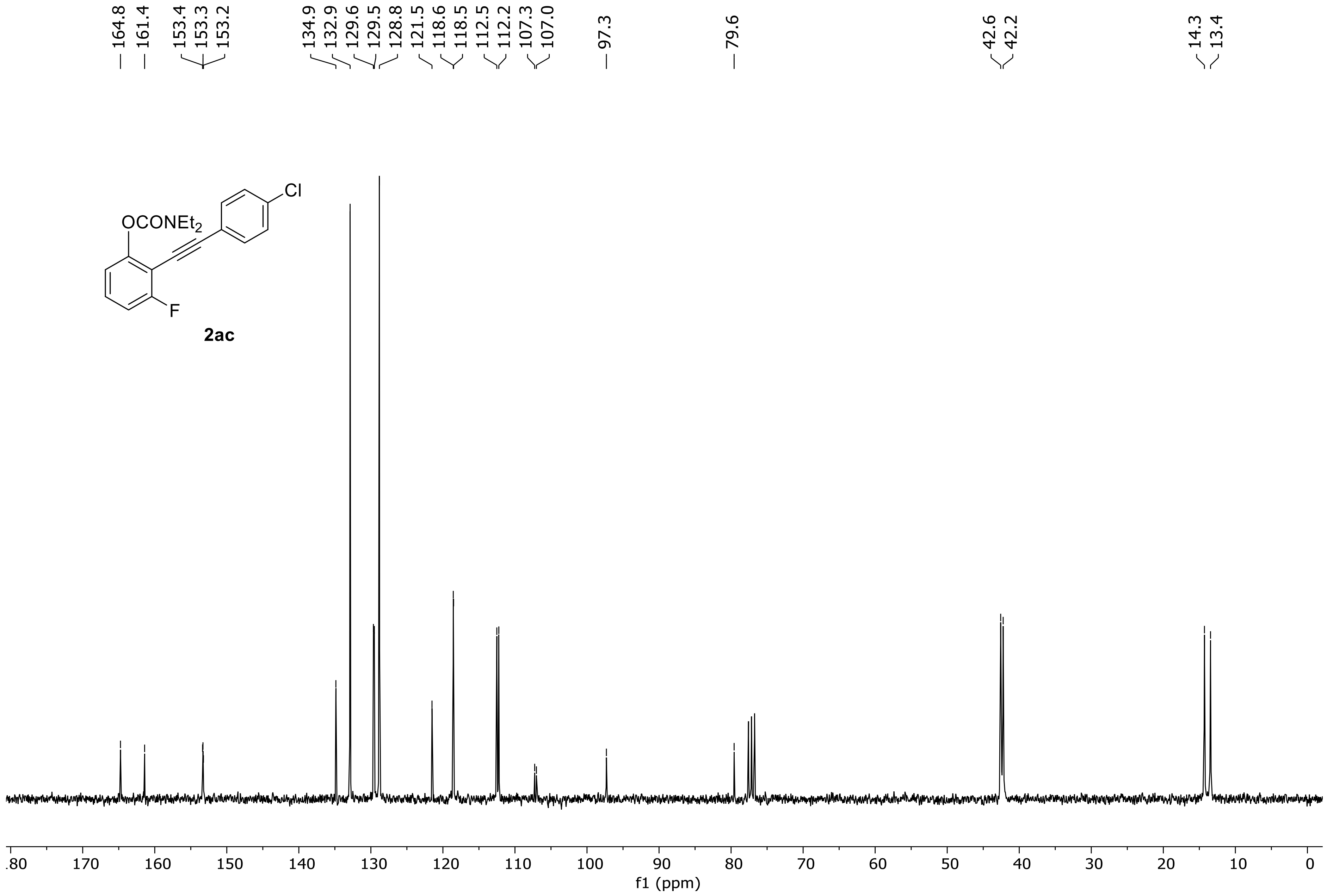

$^{1}H$ NMR (300 MHz, $CDCl_3$)

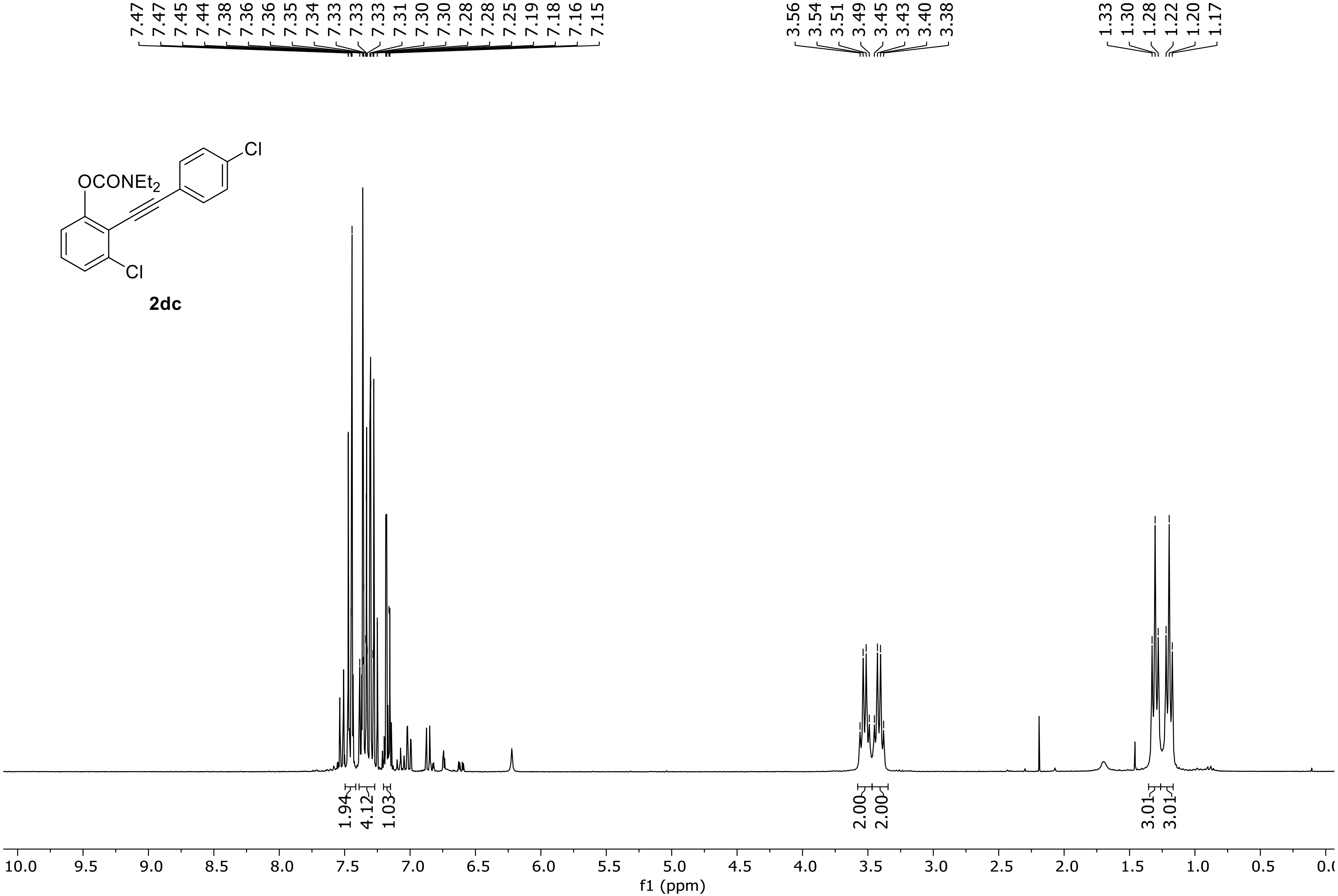

$^{13}C$ NMR (75.4 MHz, $CDCl_3$)

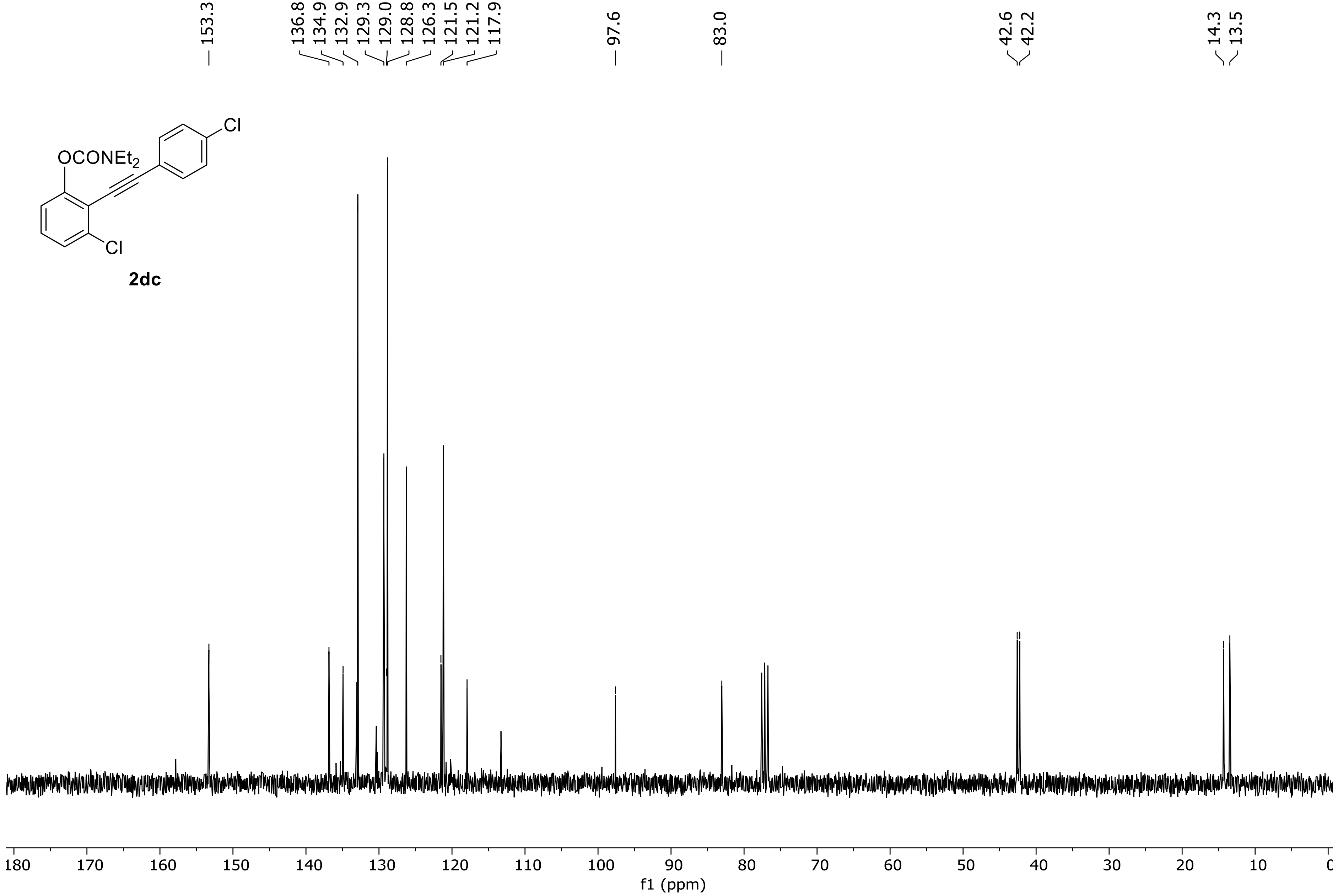

$^1$H NMR (300 MHz, $CDCl_3$)

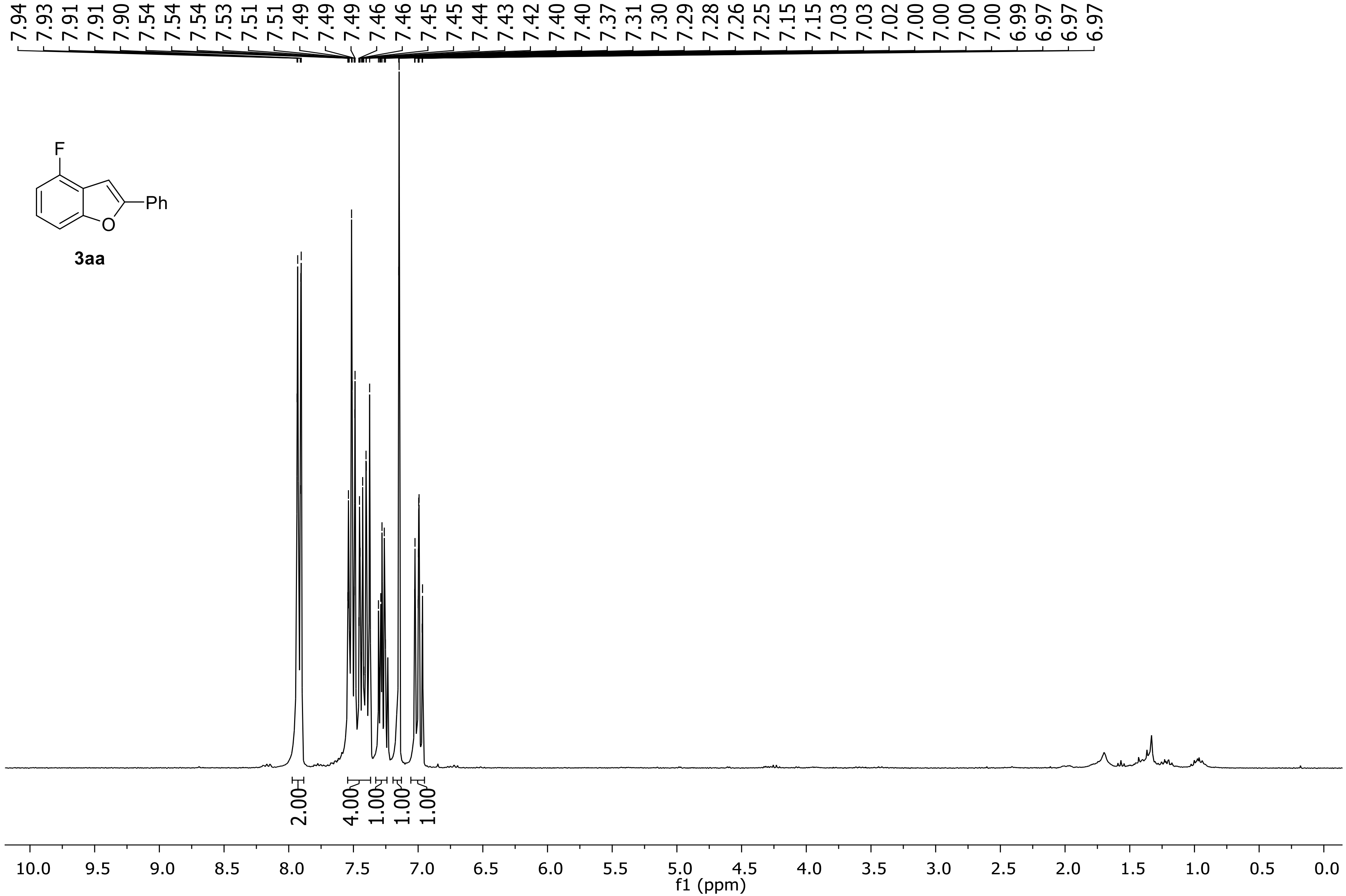

$^{13}C$ NMR (75.4 MHz, $CDCl_3$)

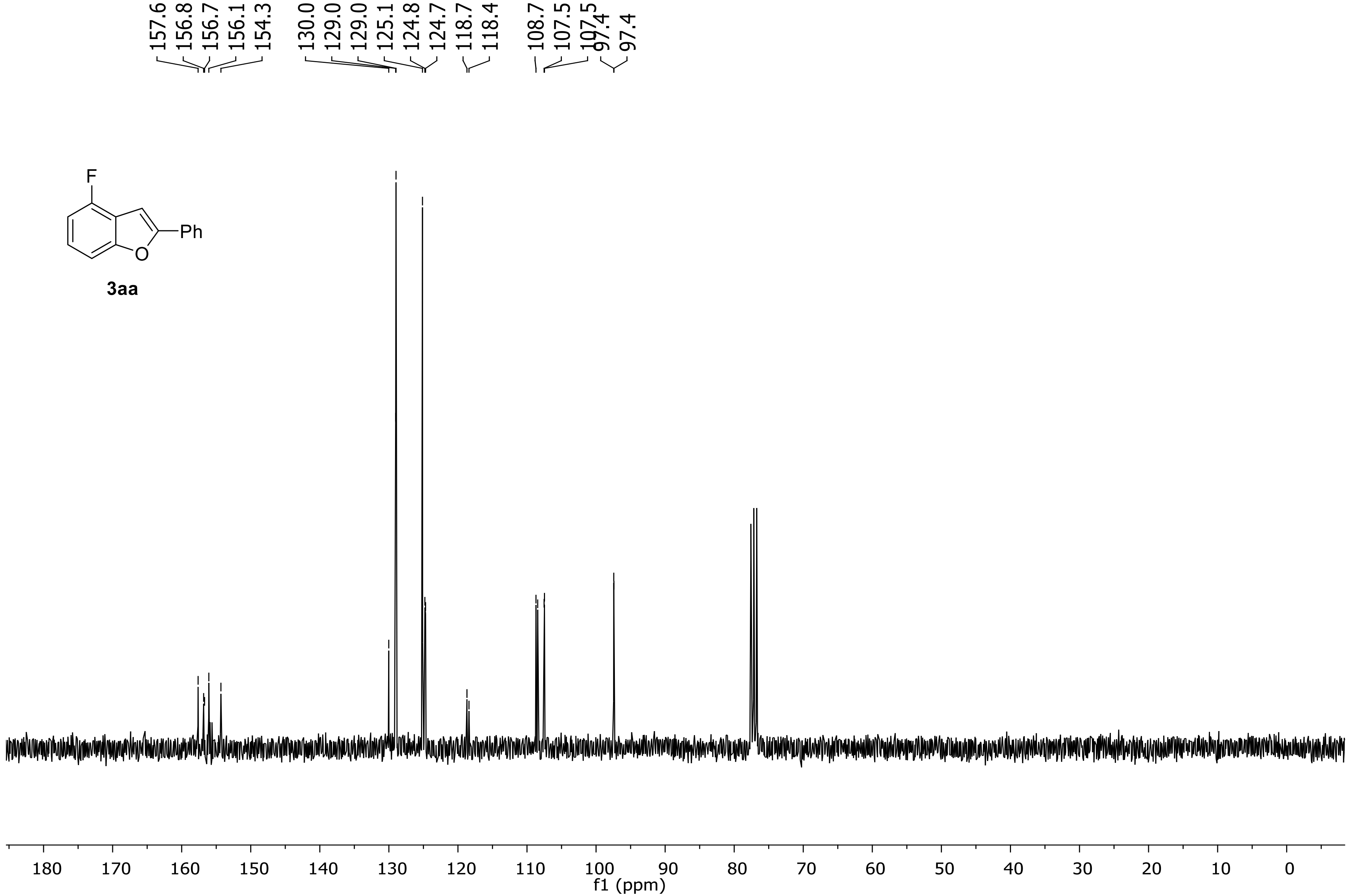

$^1H$ NMR (300 MHz, $CDCl_3$)

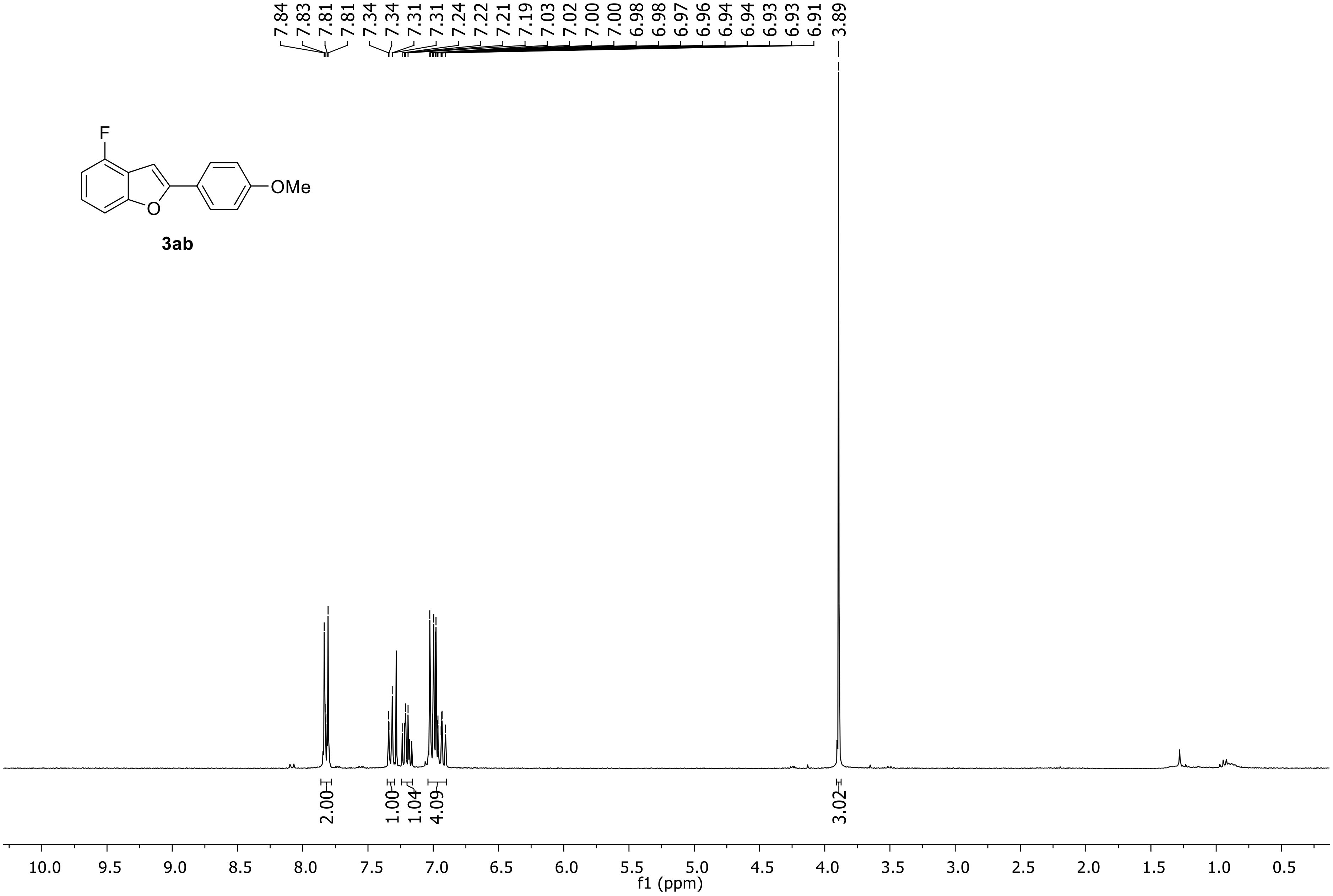

$^{13}C$ NMR (75.4 MHz, $CDCl_3$)

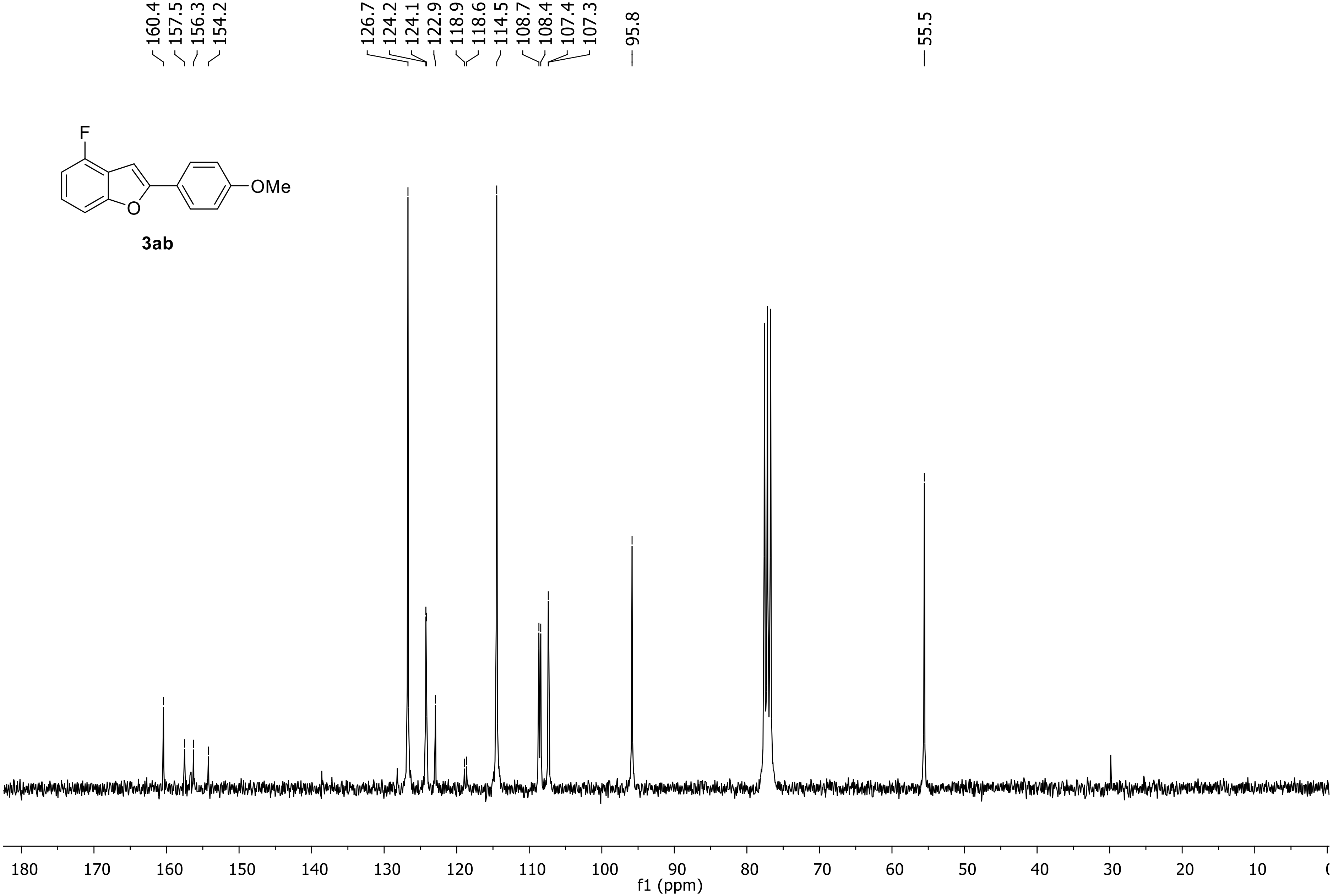

$^1H$ NMR (300 MHz, $CDCl_3$)

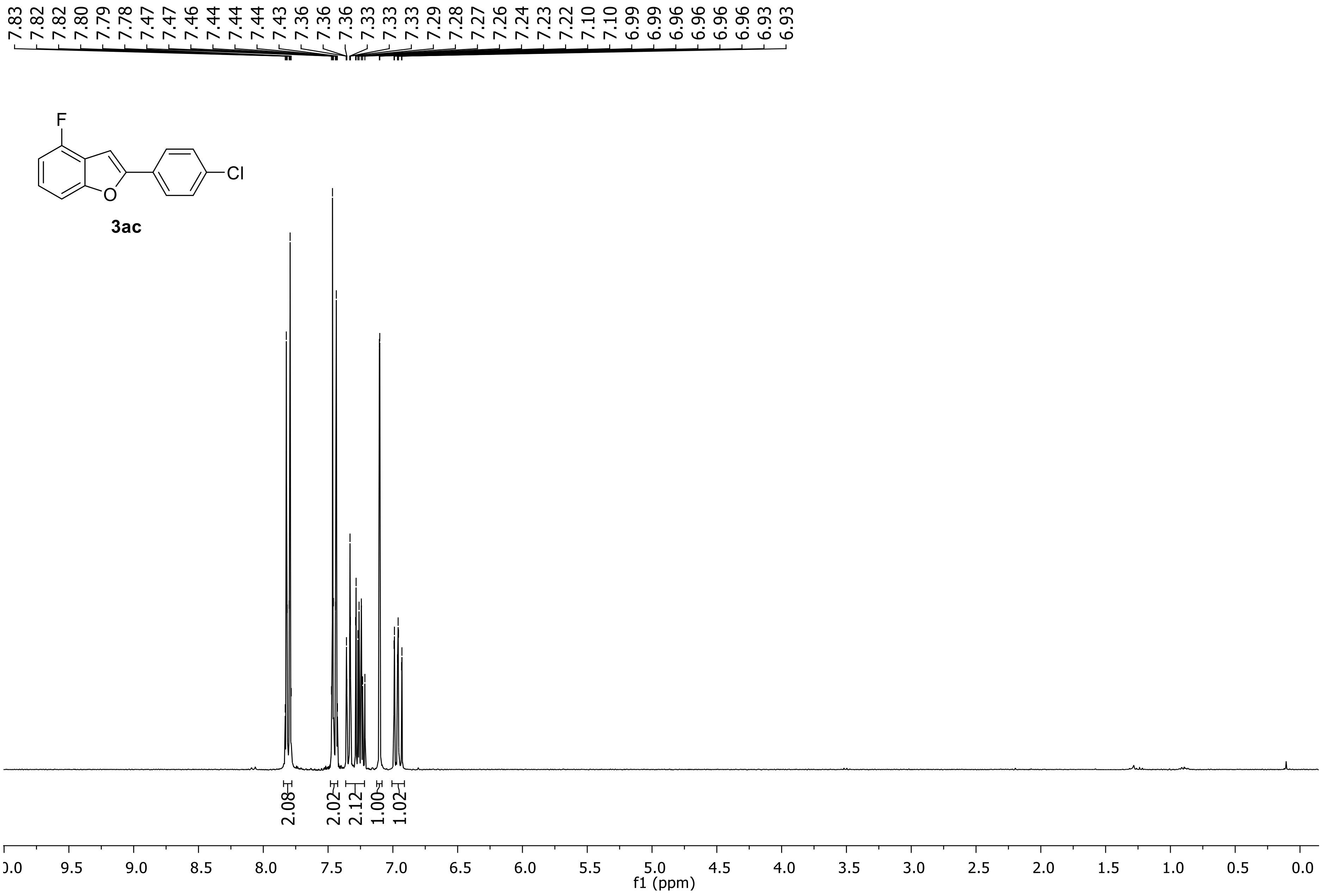


**3ac**

$^{13}C$ NMR (75.4 MHz, $CDCl_3$)

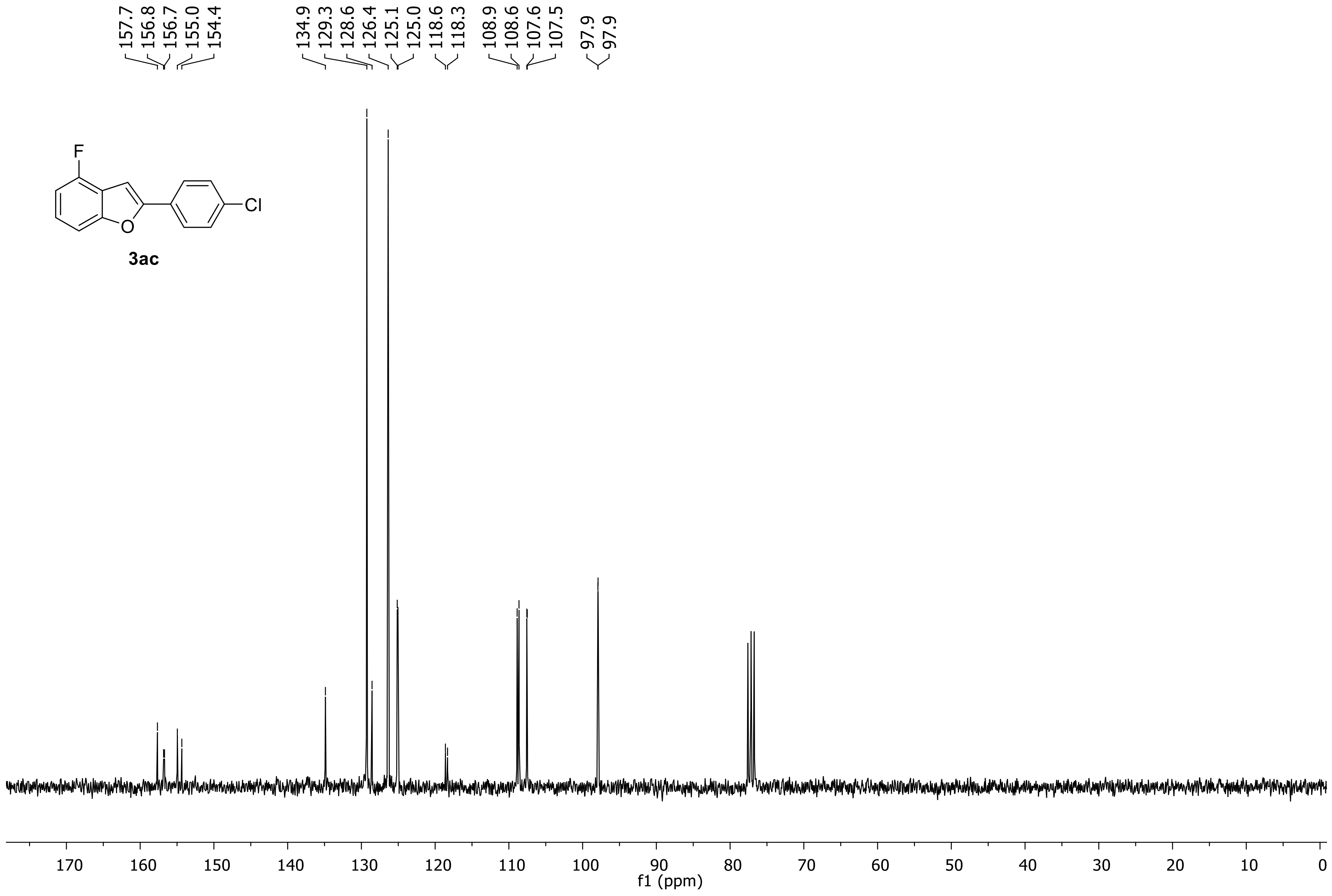


**3ac**

$^1H$ NMR (300 MHz, $CDCl_3$)

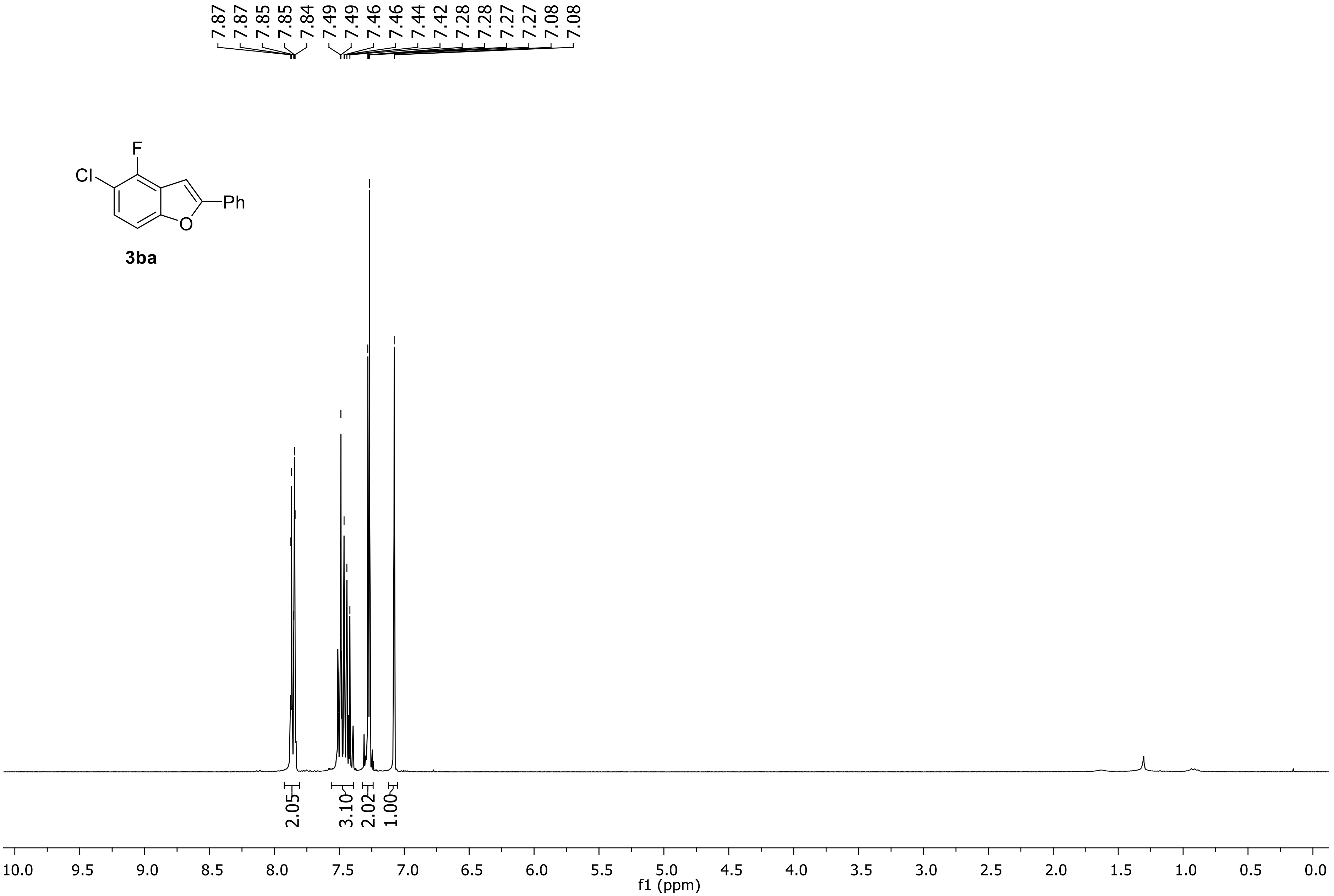

$^{13}C$ NMR (75.4 MHz, $CDCl_3$)

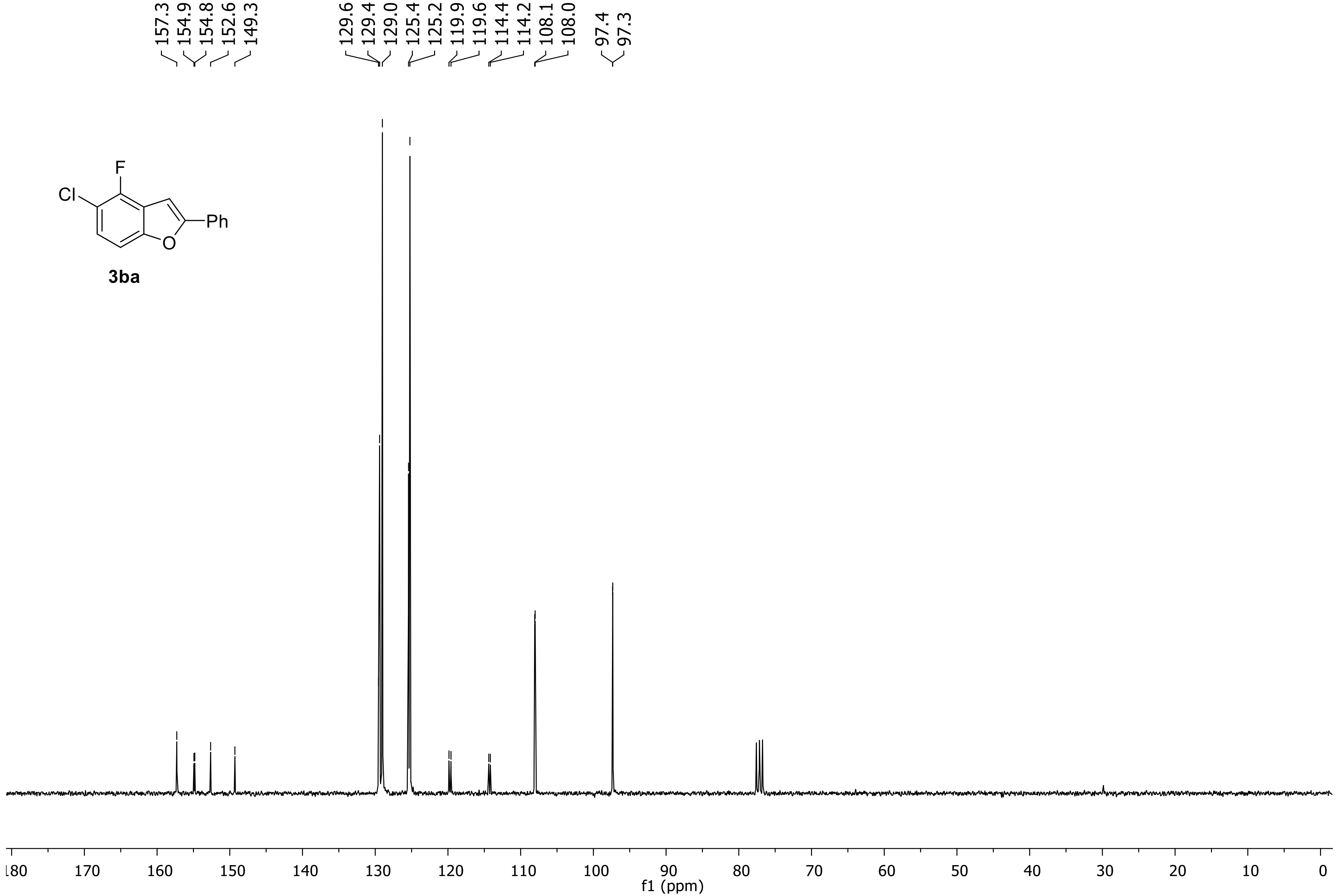

$^1H$ NMR (300 MHz, $CDCl_3$)

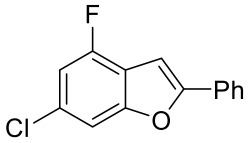

3ca

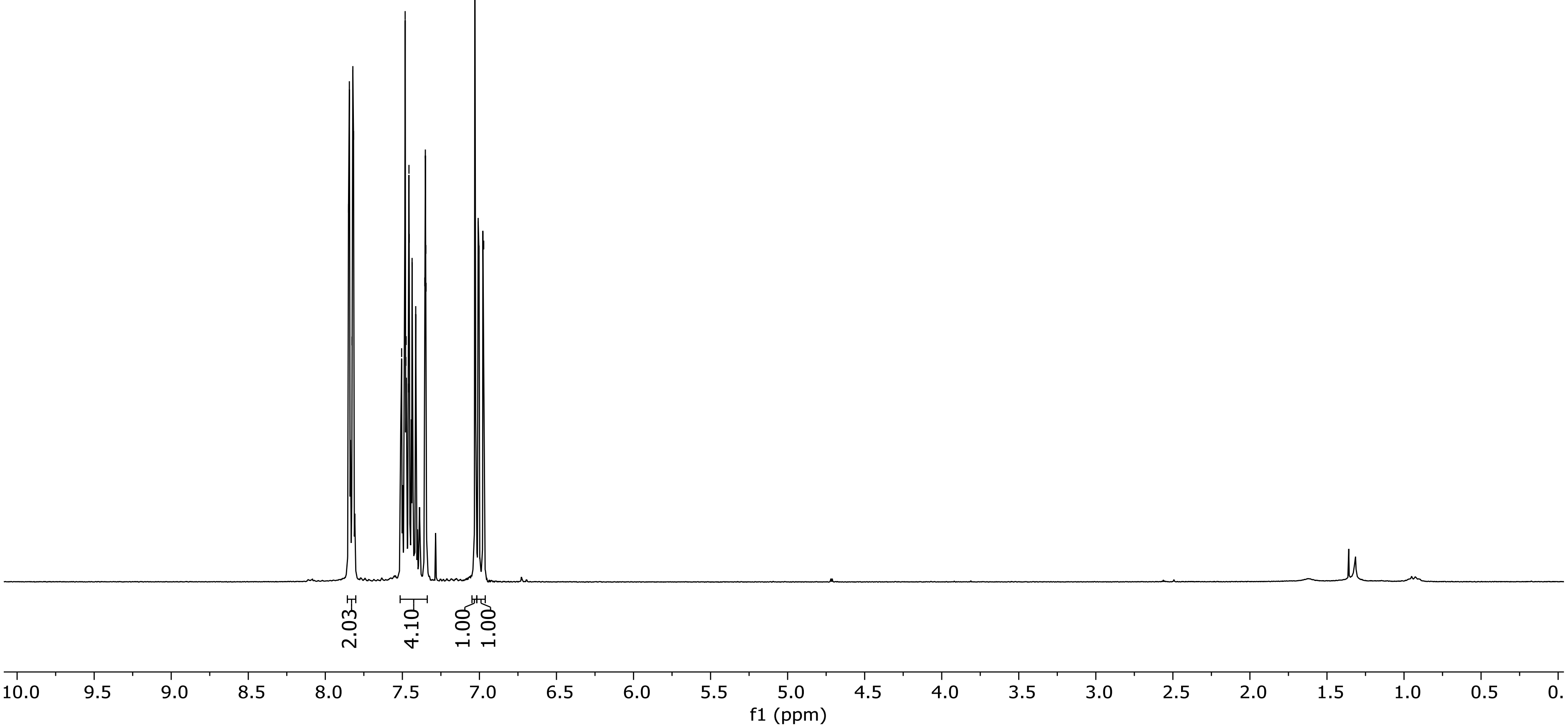

$^{13}C$ NMR (75.4 MHz, $CDCl_3$)

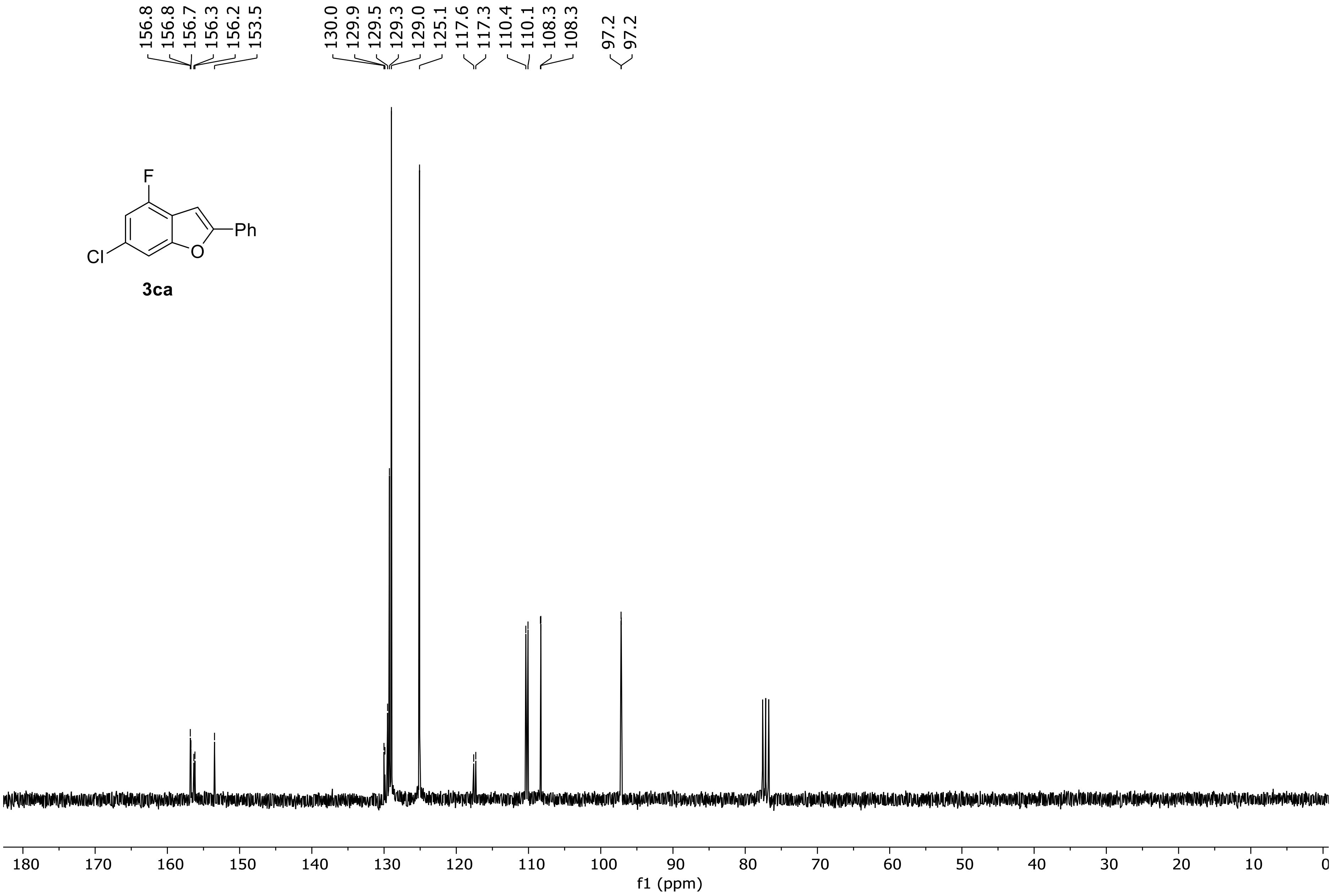

$^{1}H$ NMR (300 MHz, $CDCl_3$)

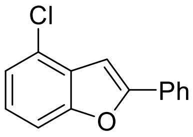


**3da**

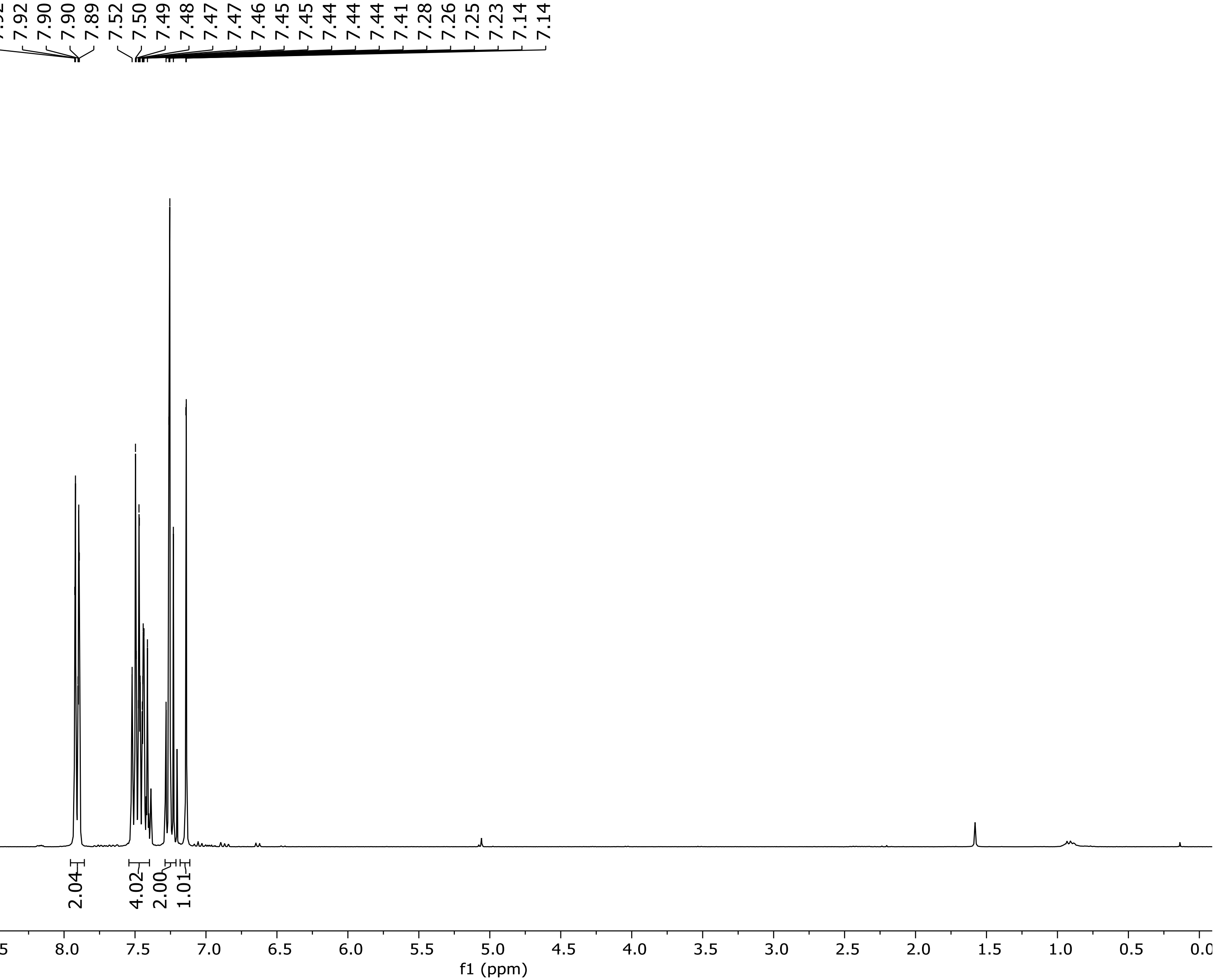

$^{13}C$ NMR (75.4 MHz, $CDCl_3$)

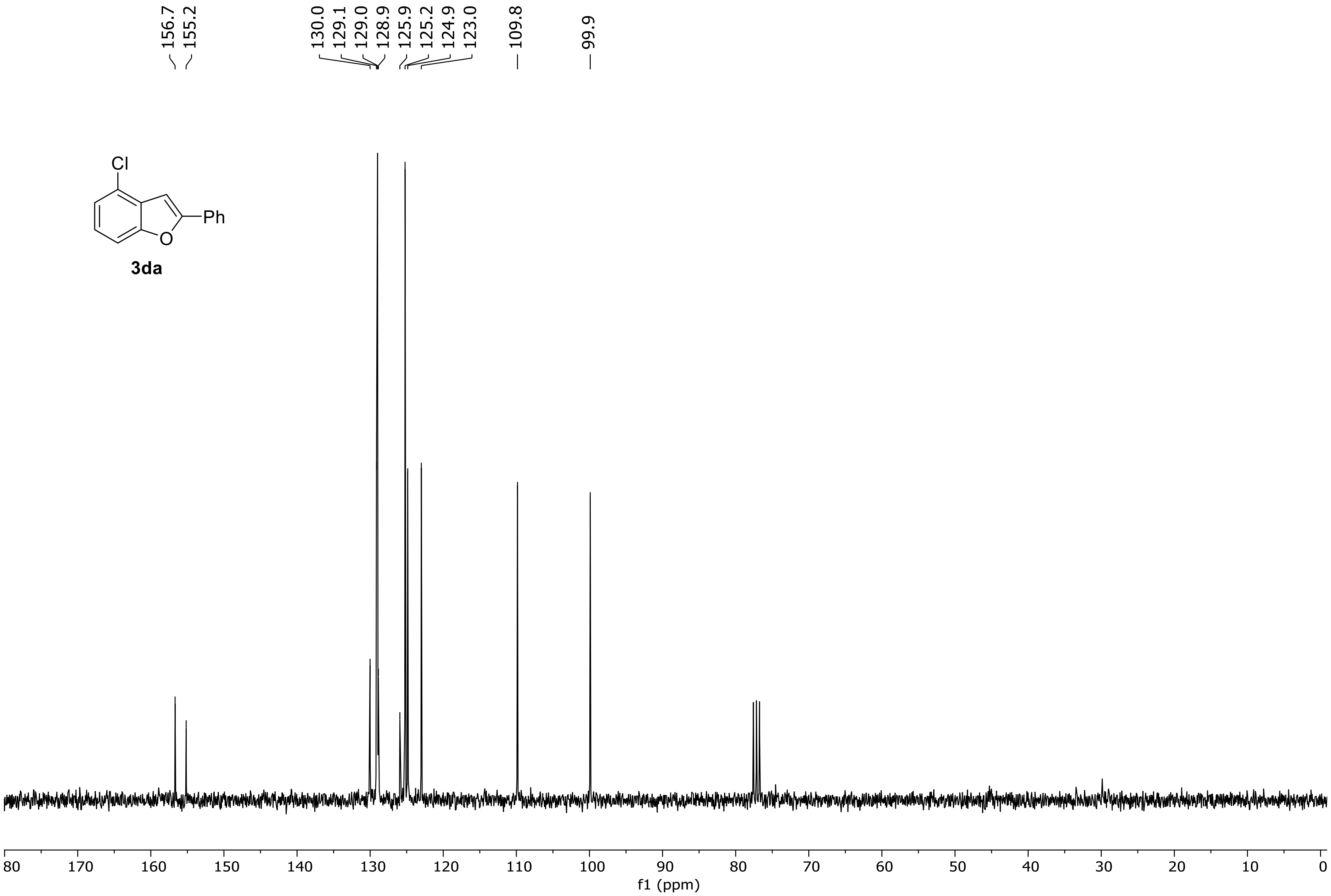


**3da**

$^1H$ NMR (300 MHz, $CDCl_3$)

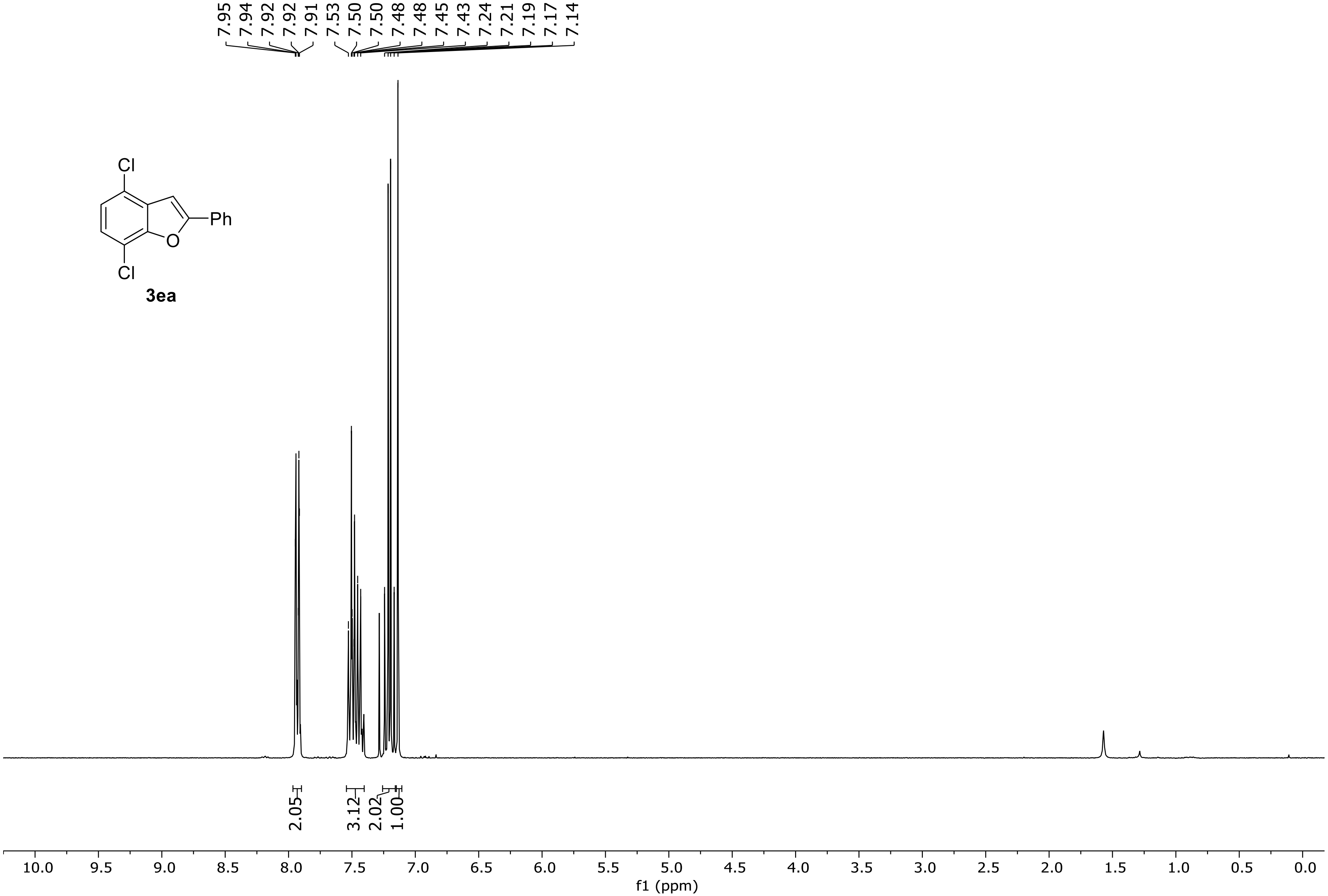


**3ea**

$^{13}C$ NMR (75.4 MHz, $CDCl_3$)

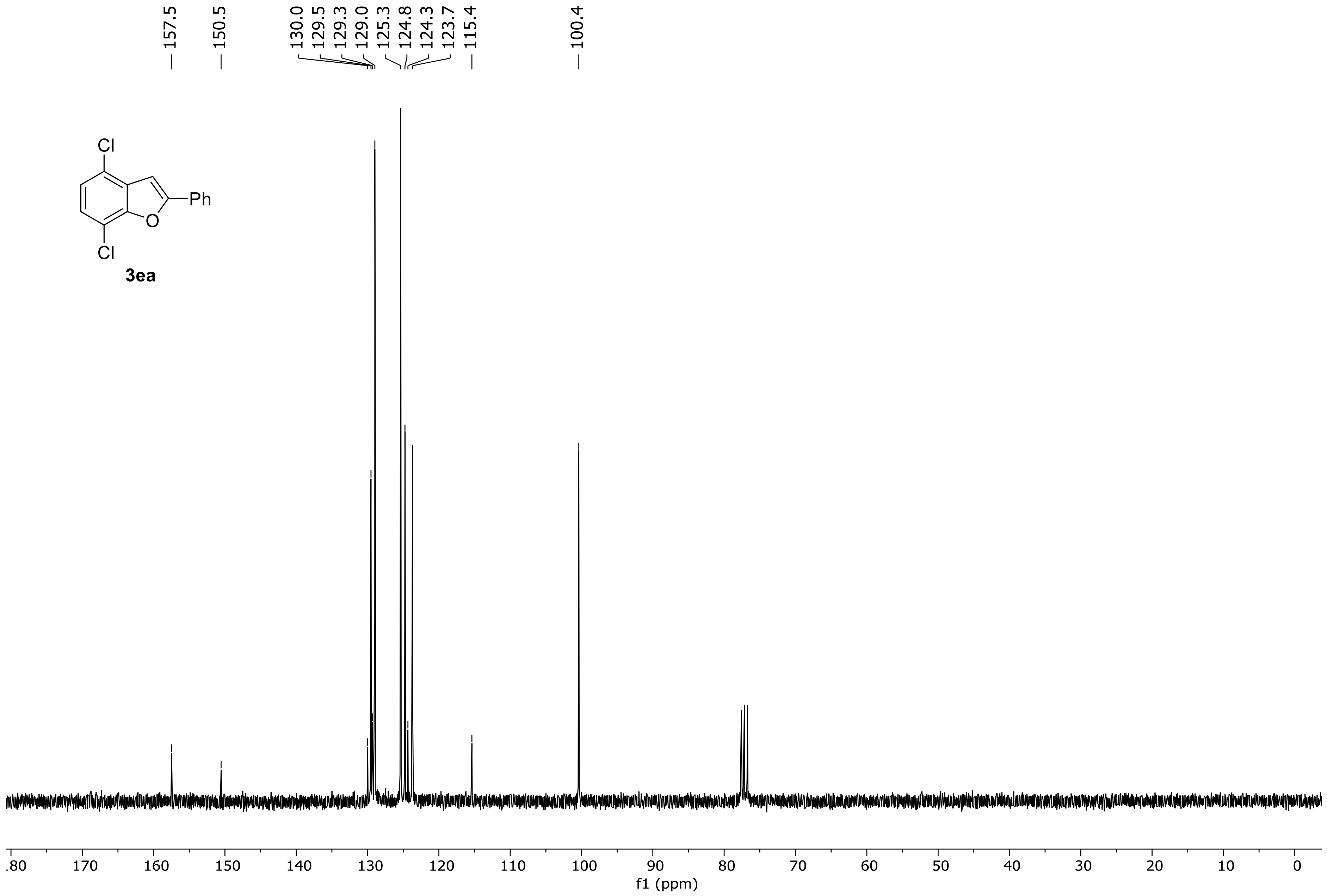

$^{1}$H NMR (300 MHz, $CDCl_3$)

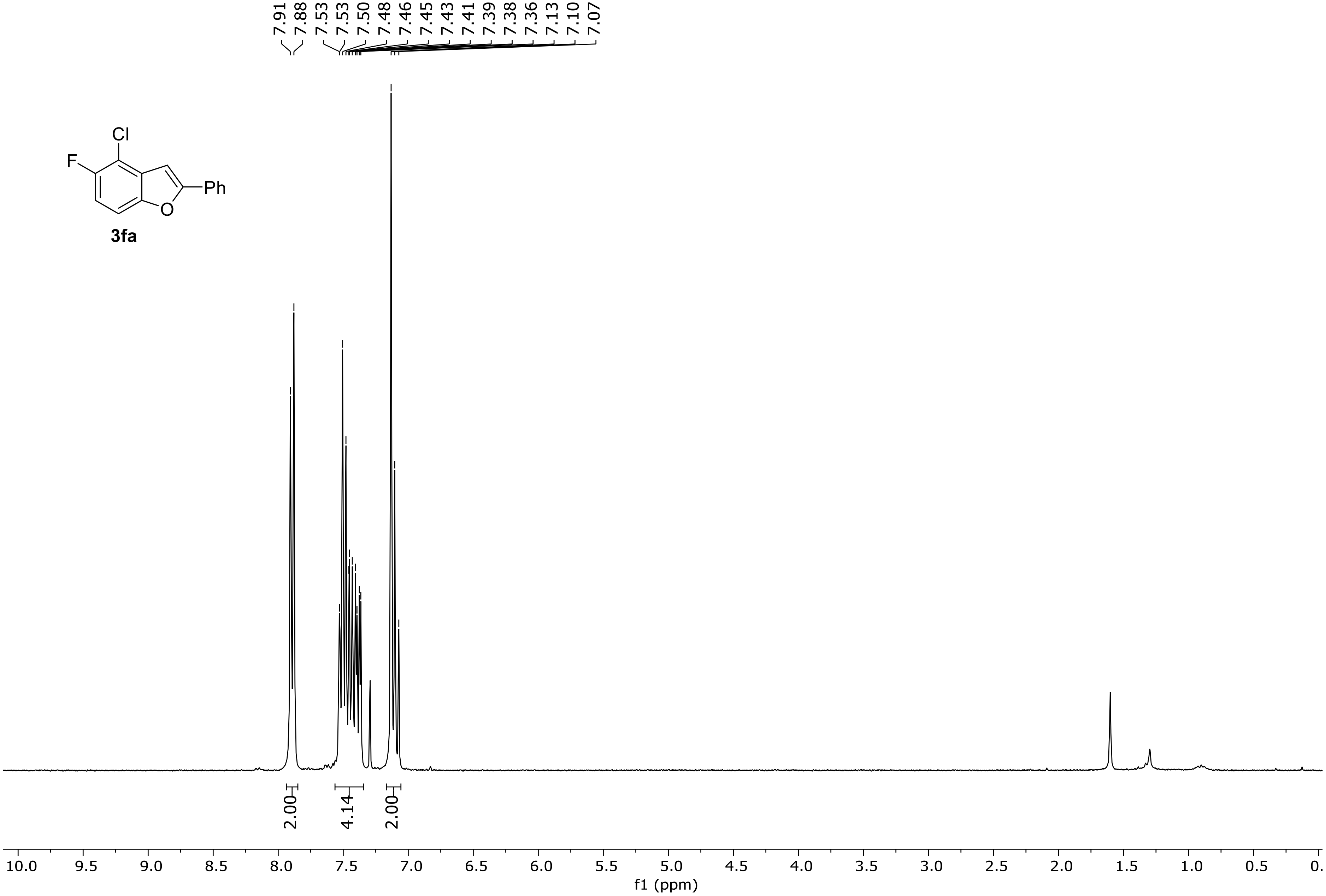

¹³C NMR (75.4 MHz, $CDCl_3$)

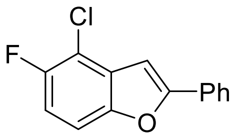


**3fa**

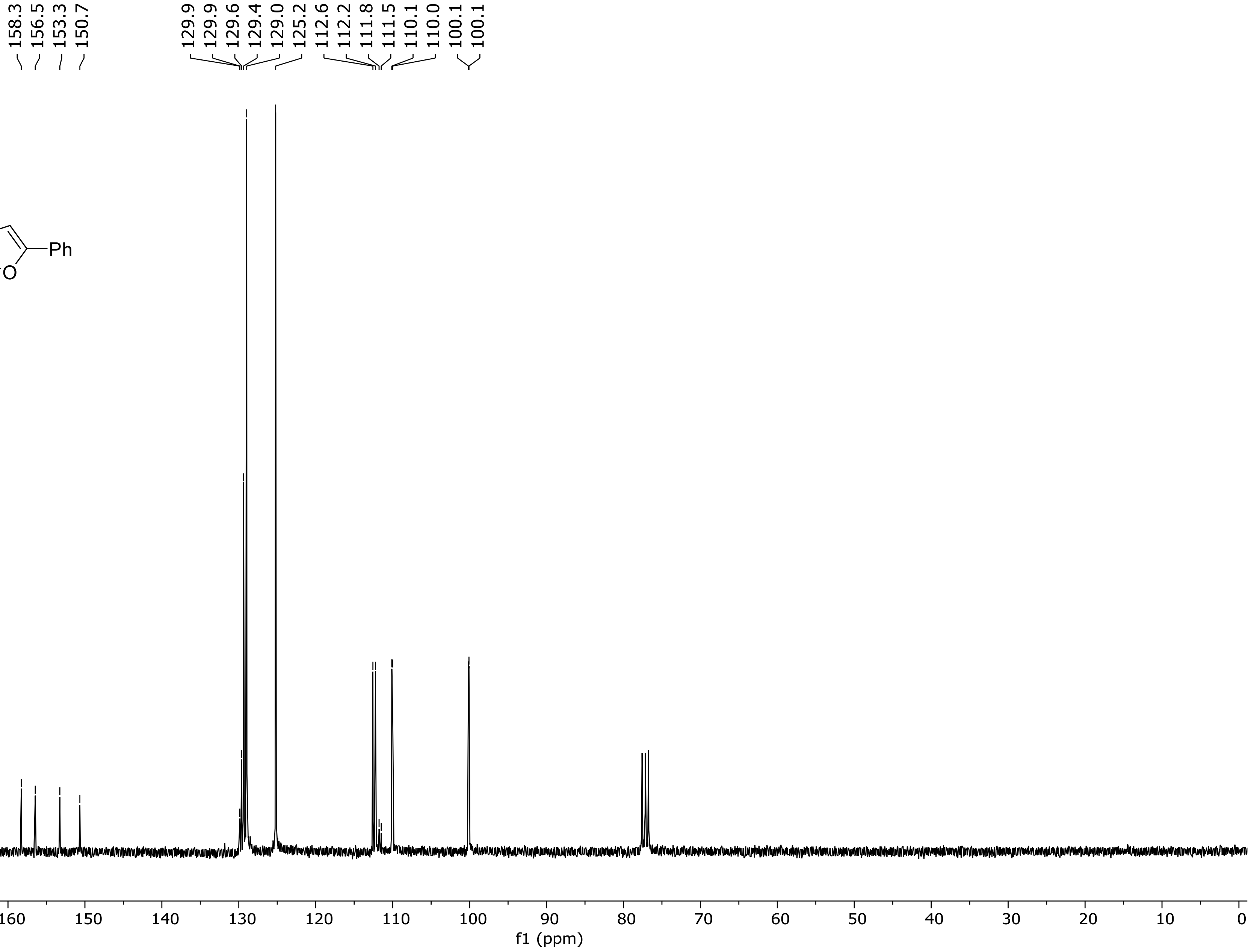

$^1$H NMR (300 MHz, $CDCl_3$)

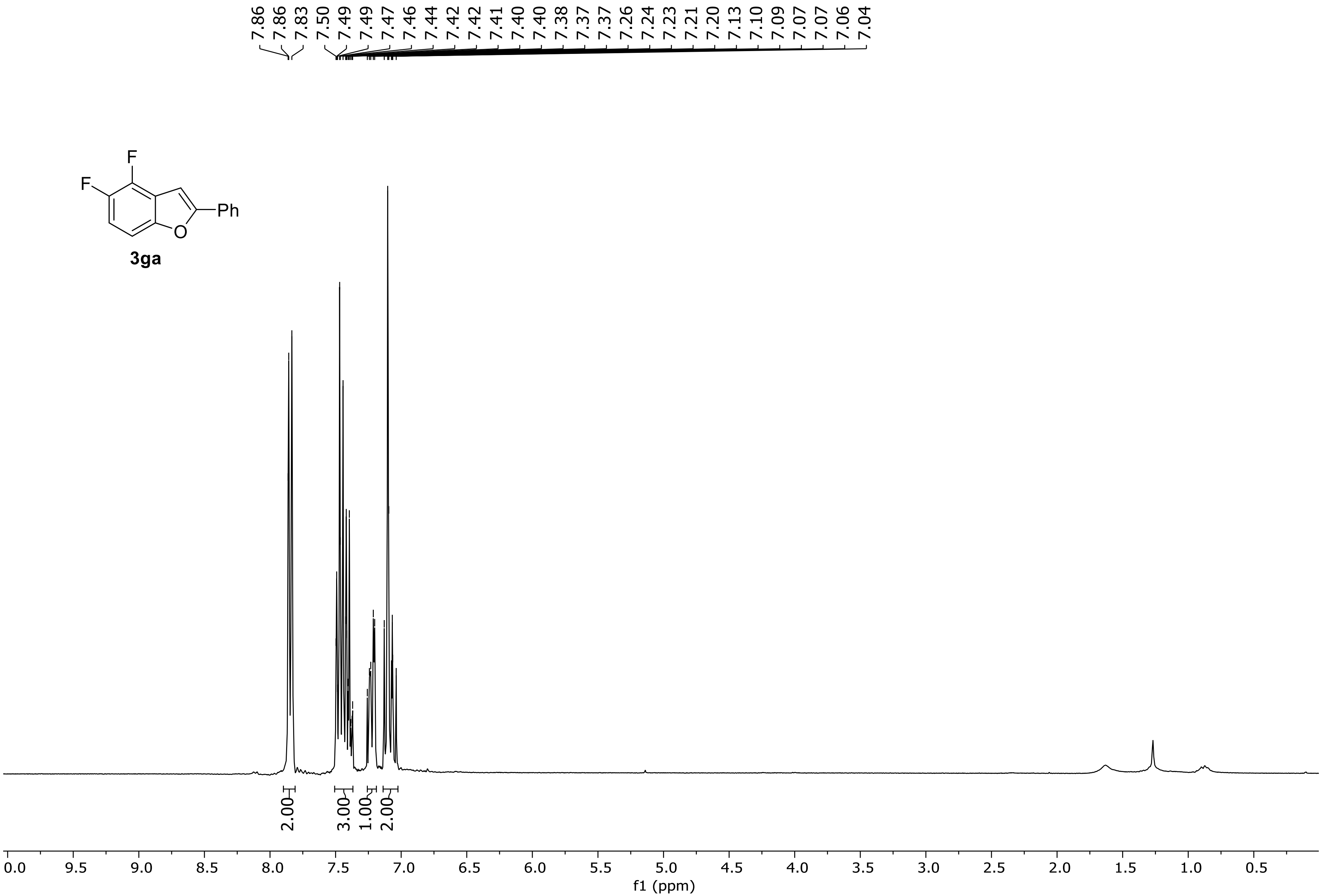


**3ga**

$^{13}C$ NMR (75.4 MHz, $CDCl_3$)

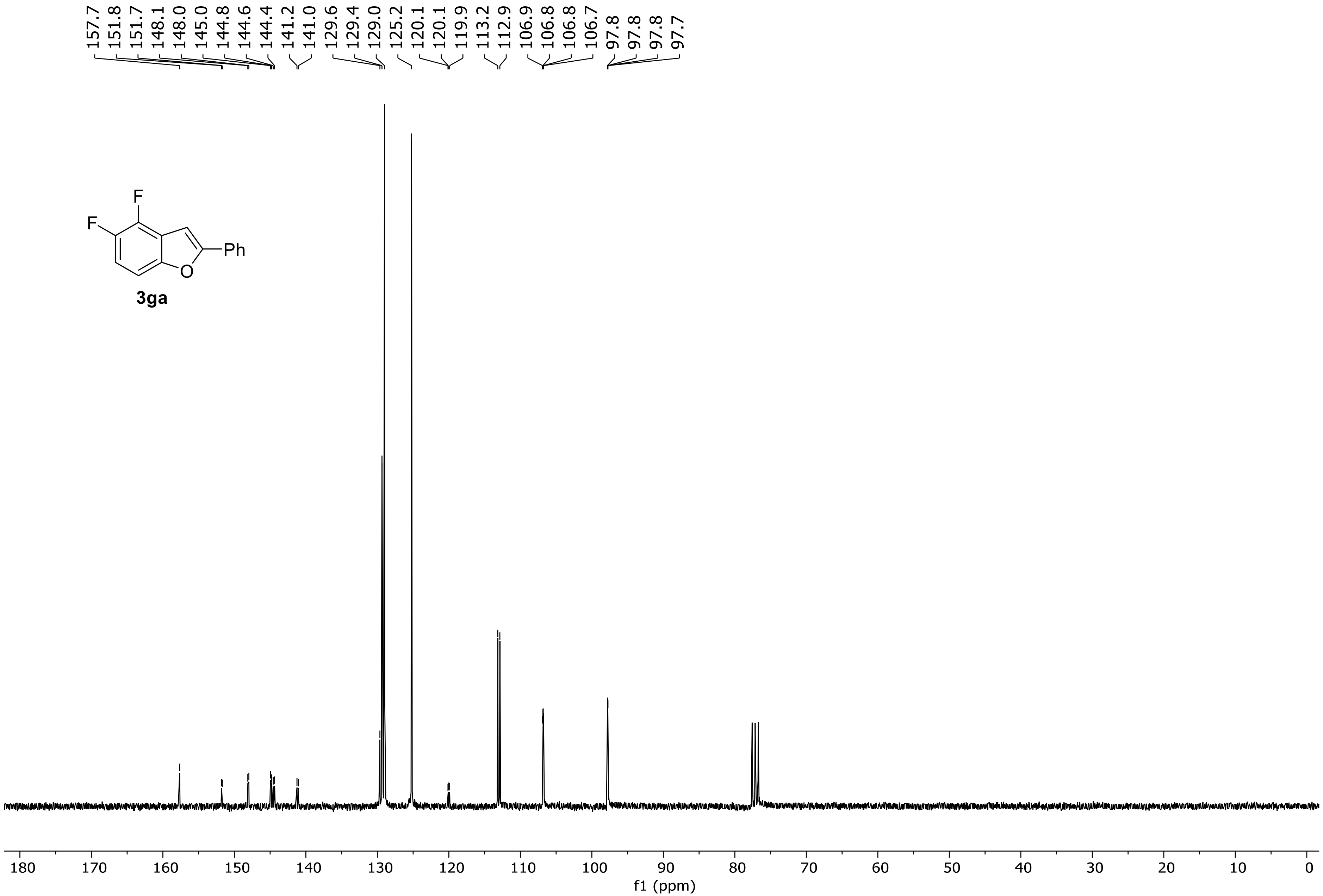


**3ga**

$^{1}$H NMR (300 MHz, $CDCl_3$)

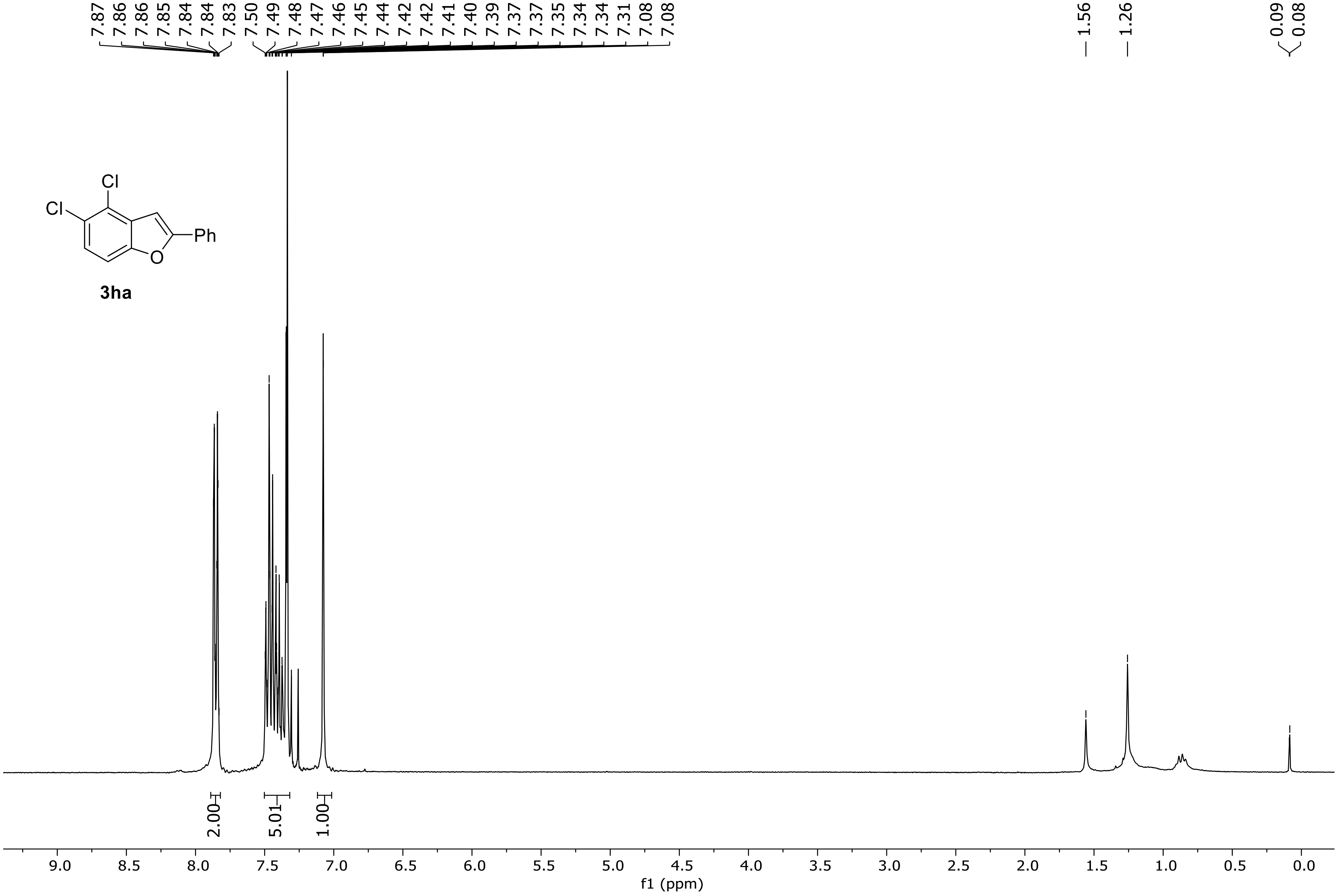


**3ha**

$^{13}C$ NMR (75.4 MHz, $CDCl_3$)

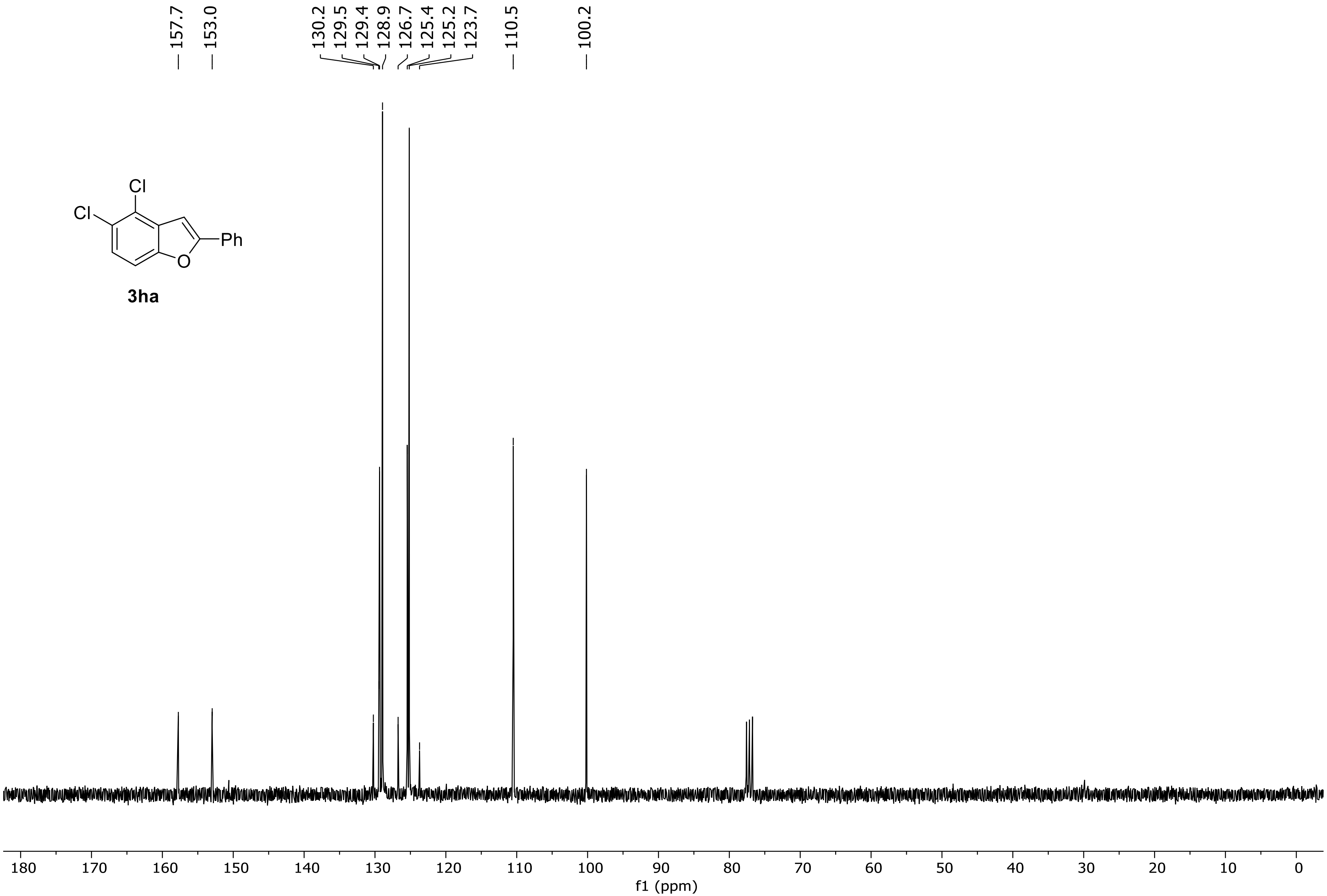


**3ha**

$^{1}H$ NMR (300 MHz, $CDCl_3$)

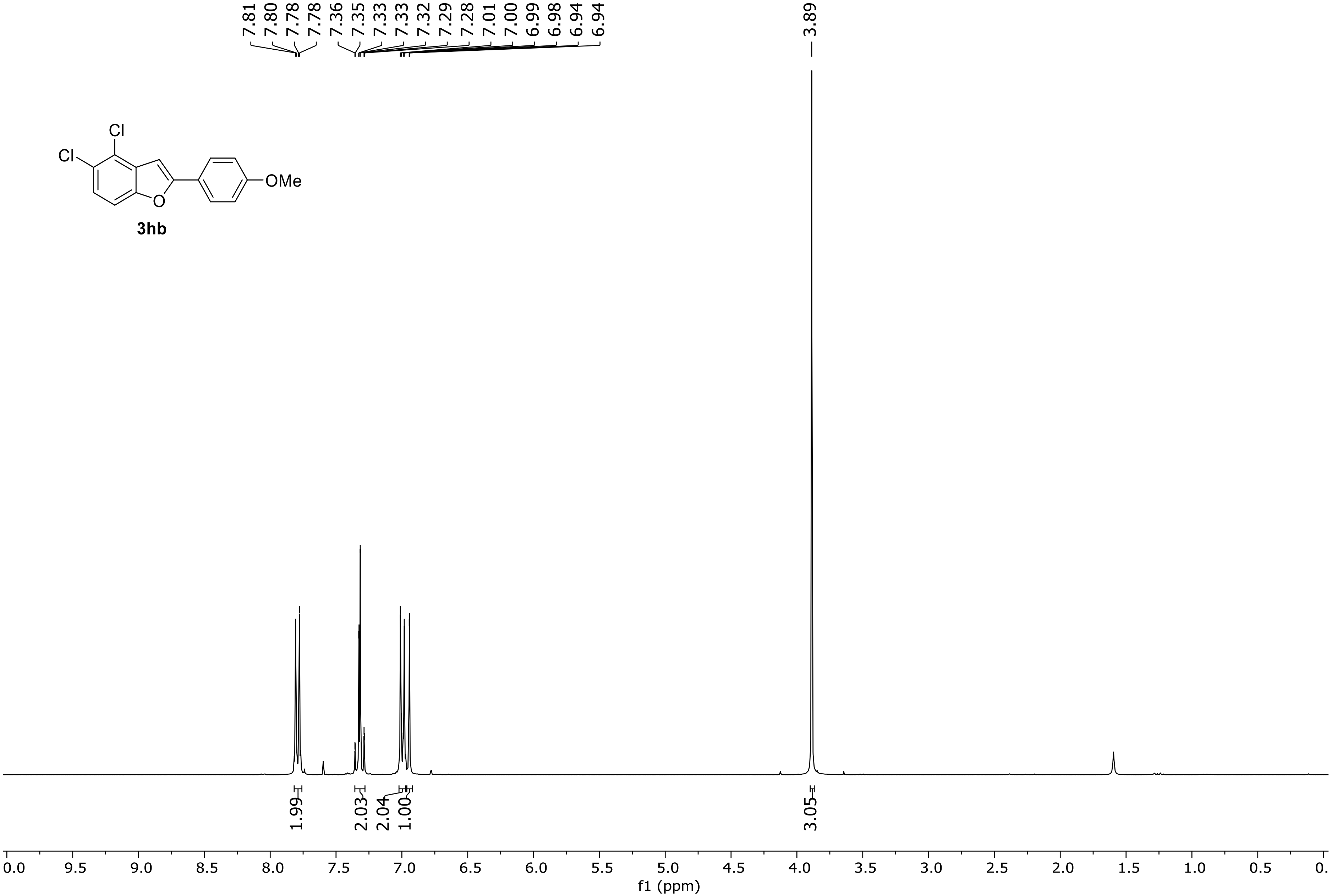


**3hb**

$^{13}C$ NMR (75.4 MHz, $CDCl_3$)

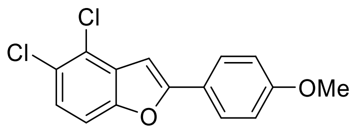


3hb

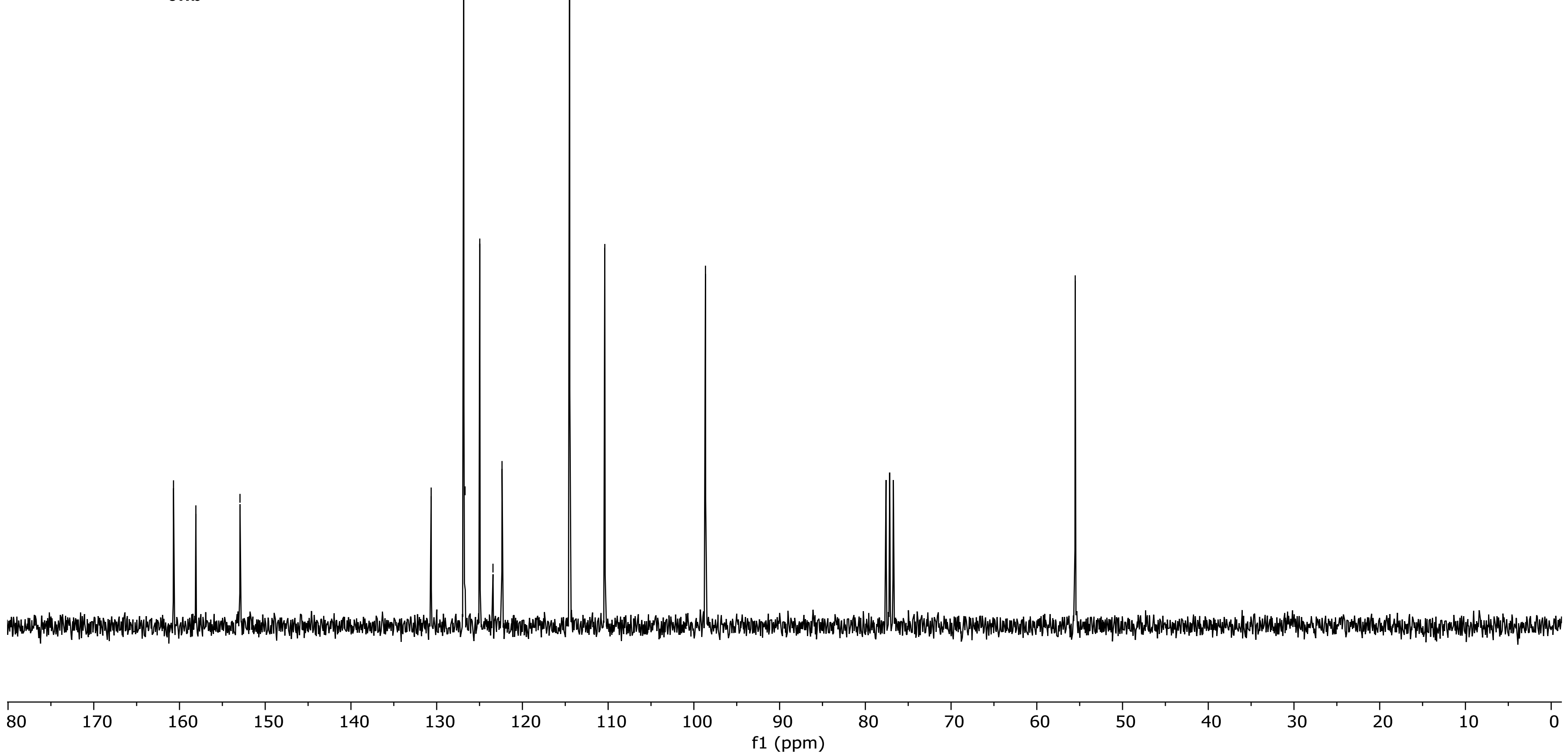

¹H NMR (300 MHz, $CDCl_3$)

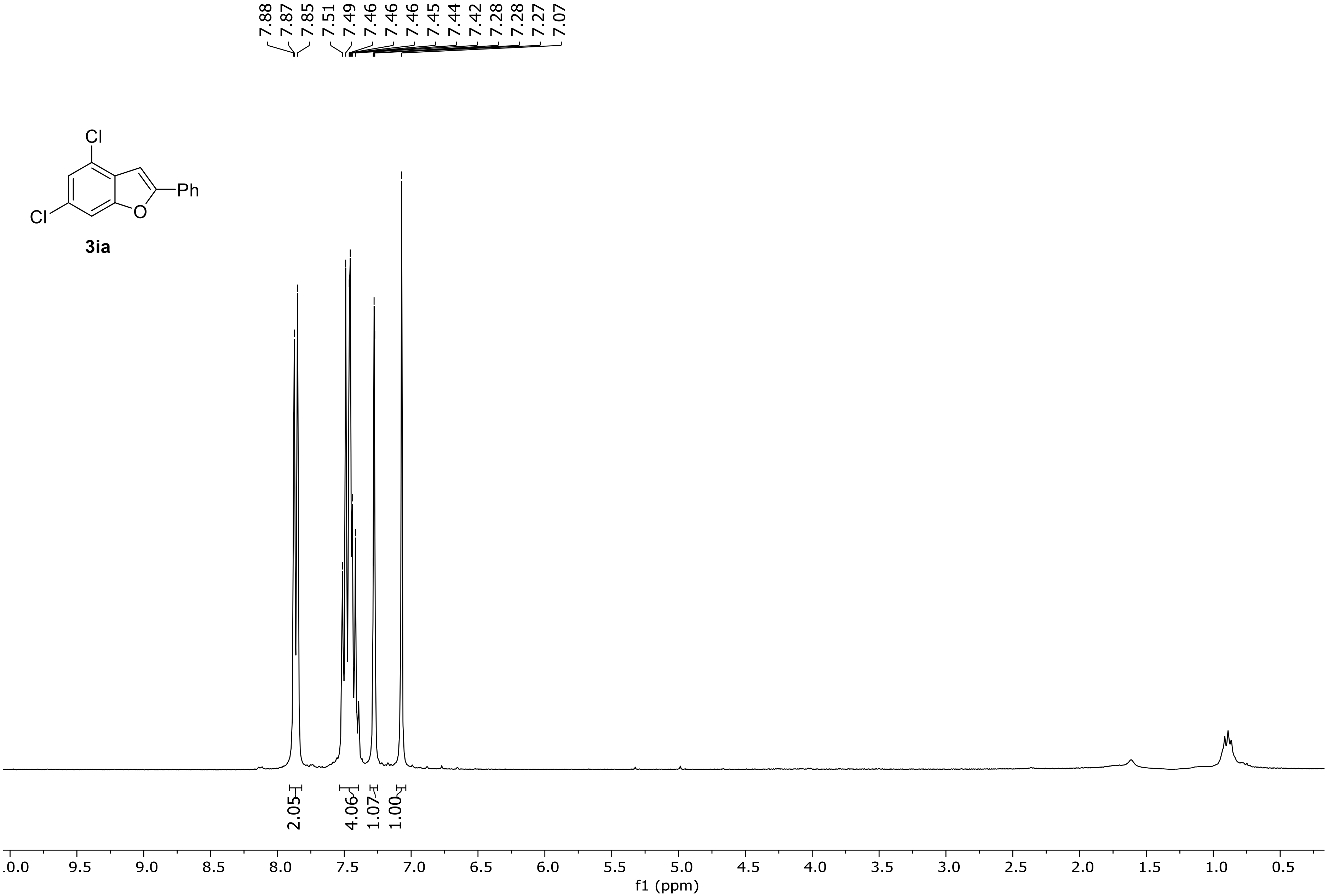

$^{13}C$ NMR (75.4 MHz, $CDCl_3$)

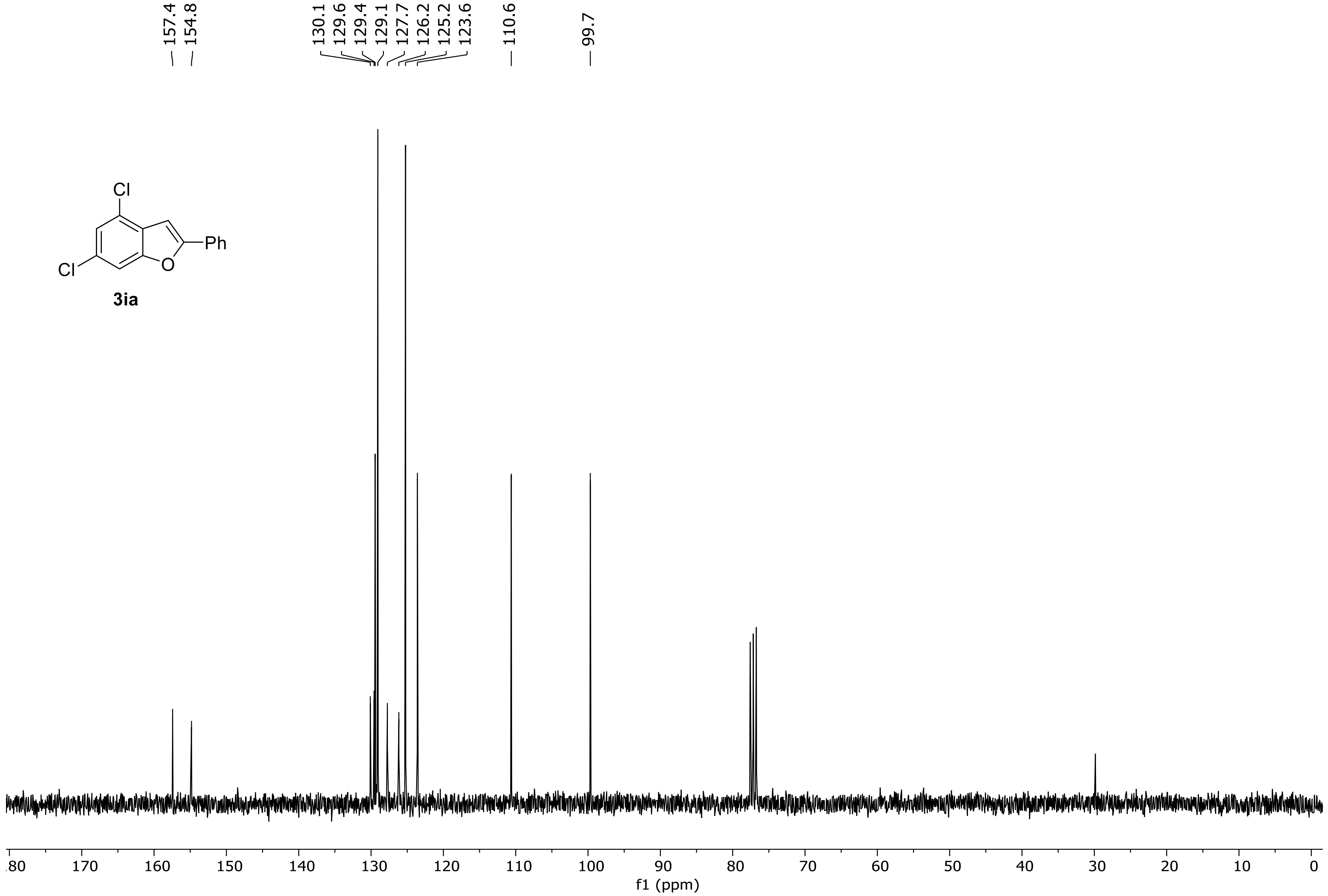

$^1H$ NMR (300 MHz, $CDCl_3$)

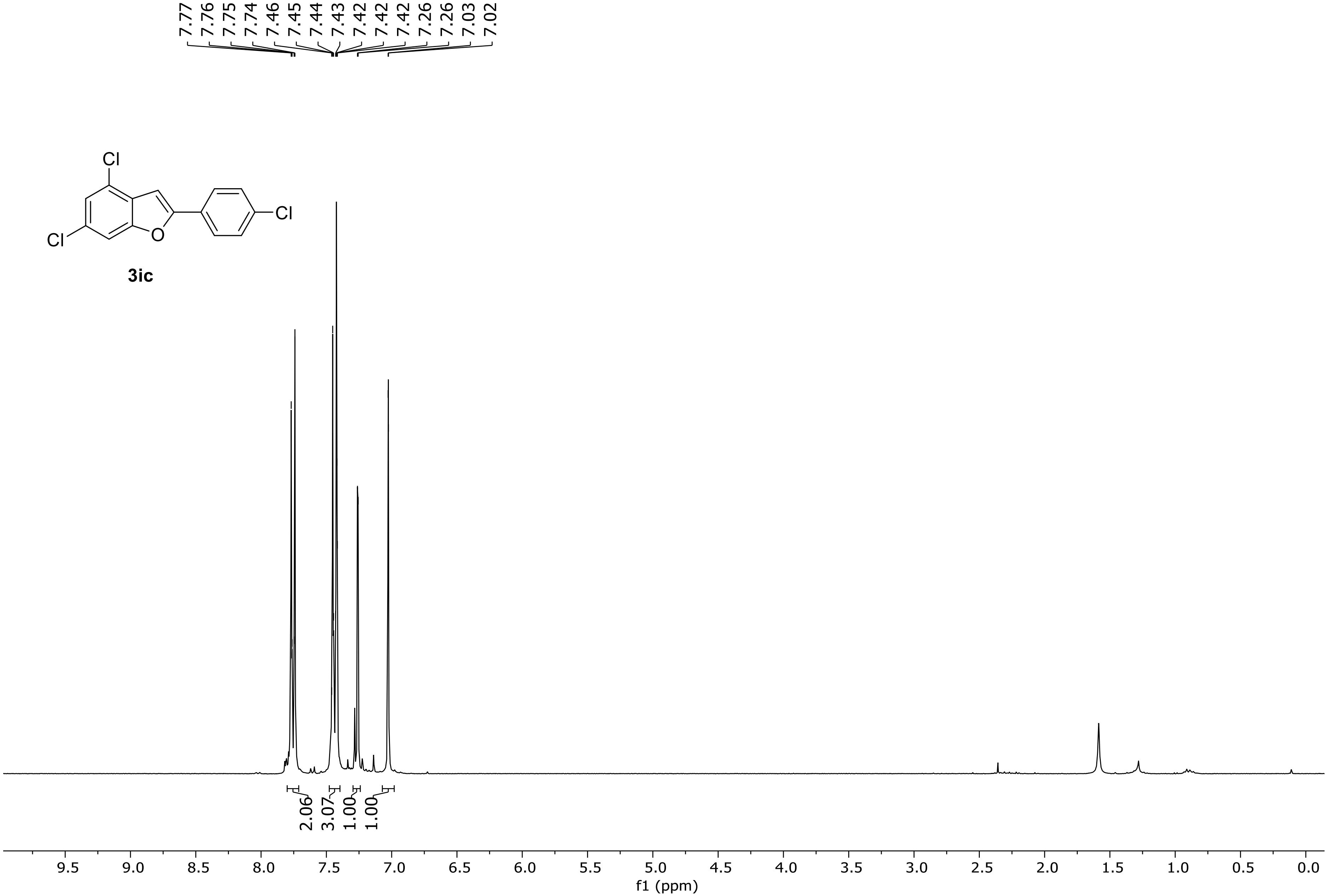

$^{13}C$ NMR (75.4 MHz, $CDCl_3$)

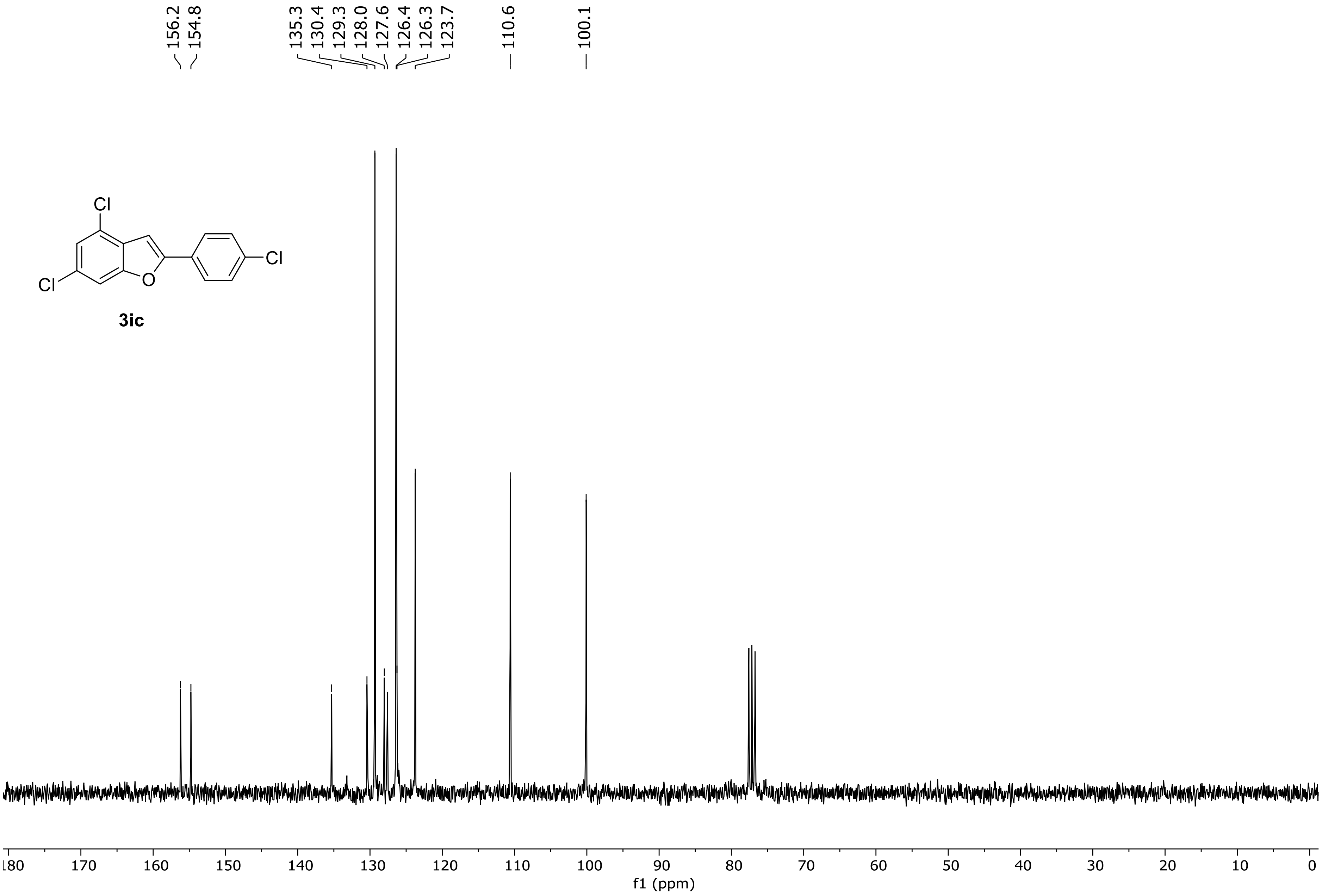

¹H NMR (300 MHz, $CDCl_3$)

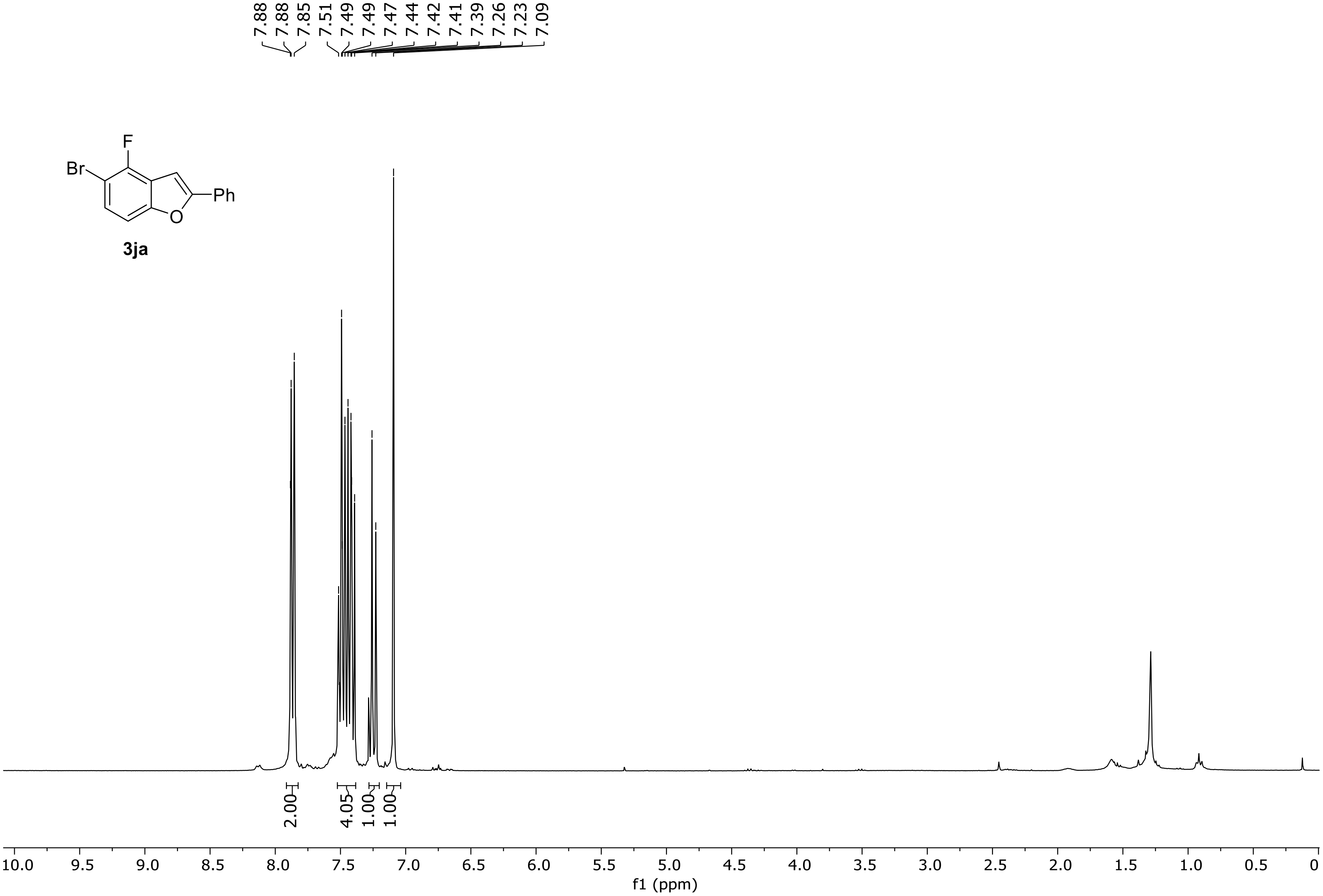

$^{13}C$ NMR (75.4 MHz, $CDCl_3$)

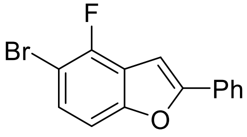


**3ja**

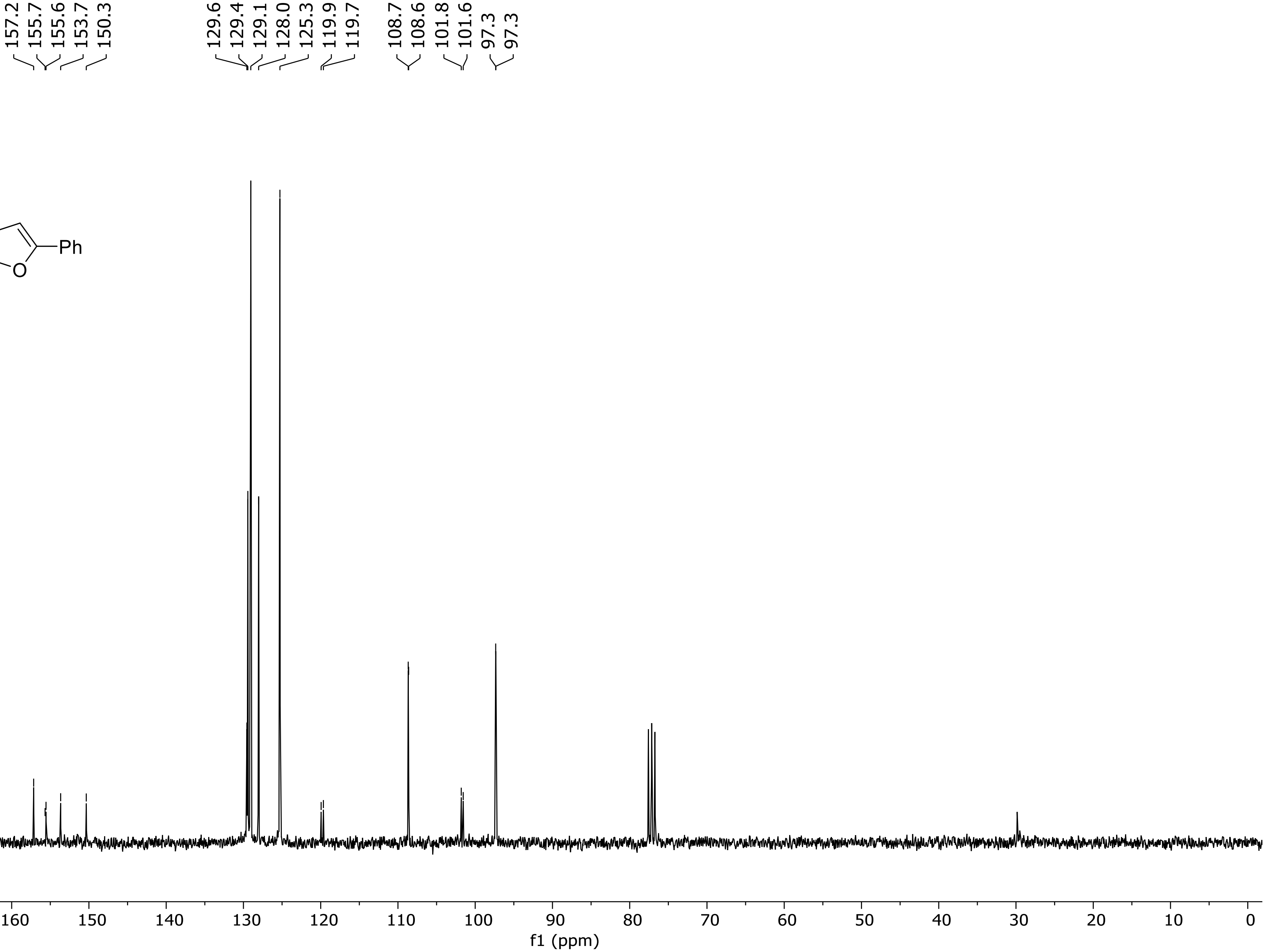

1H NMR (300 MHz, $CDCl_3$)

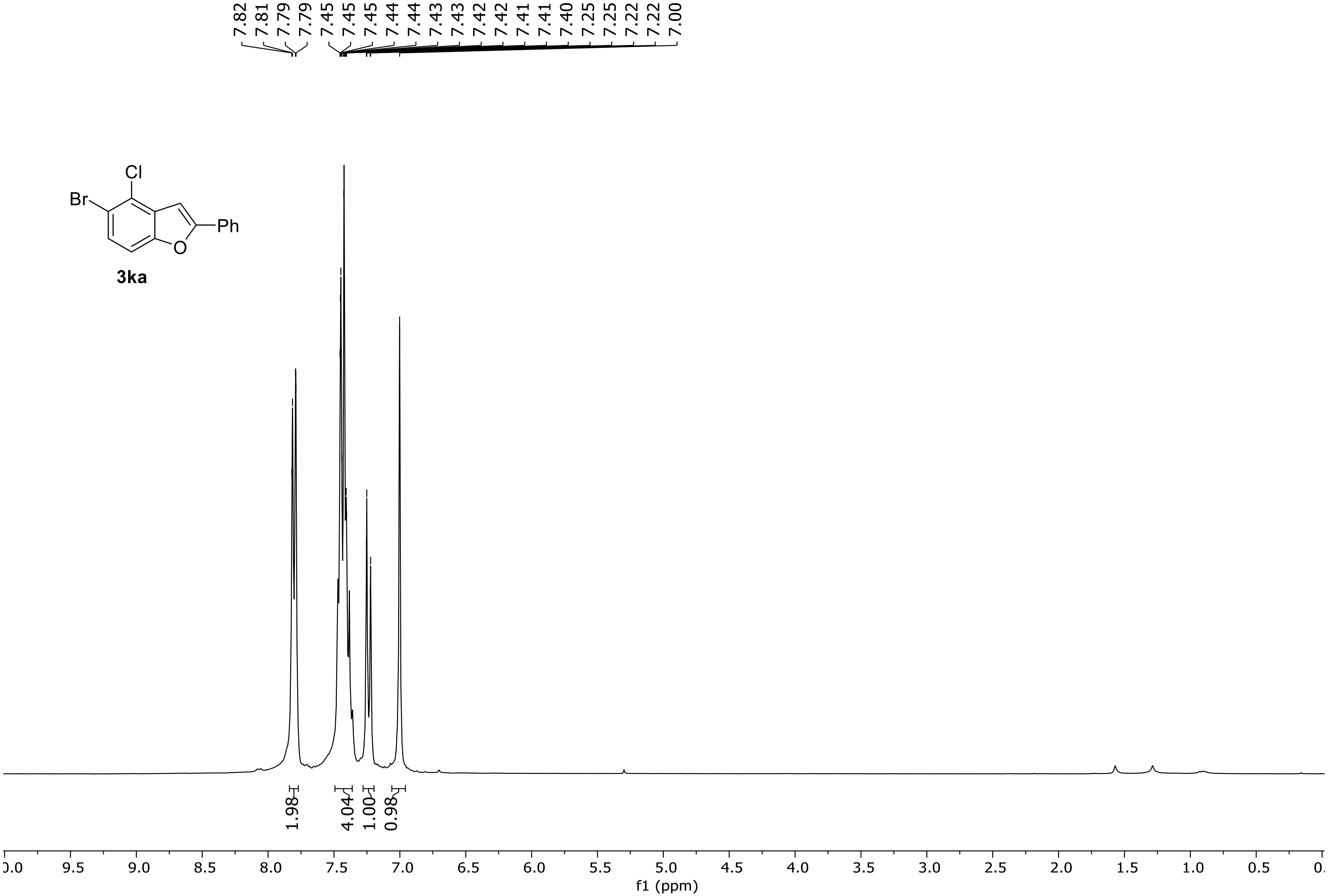

$^{13}C$ NMR (75.4 MHz, $CDCl_3$)

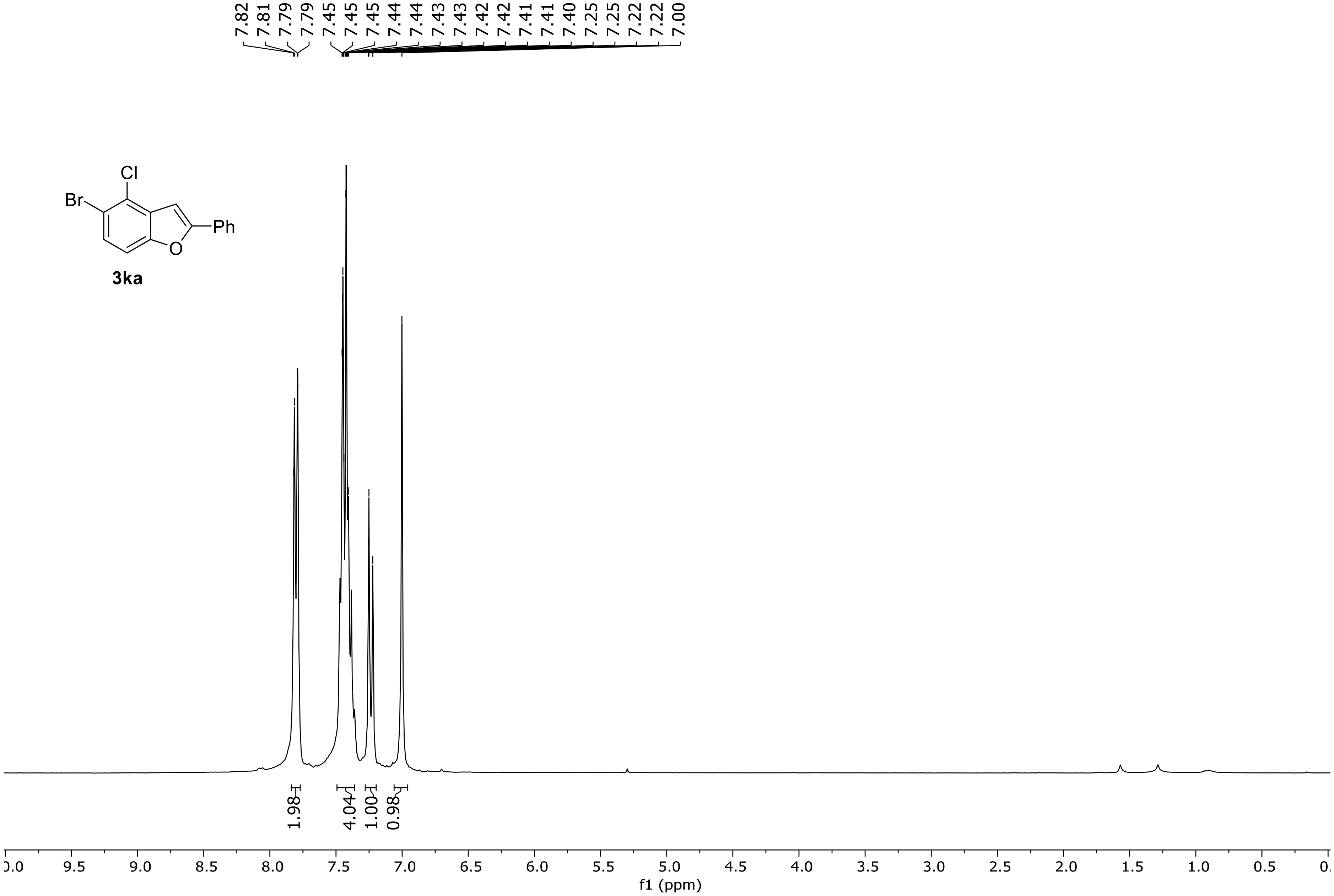

$^1H$ NMR (300 MHz, $CDCl_3$)

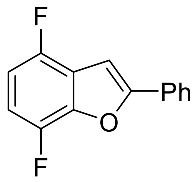


**3la**

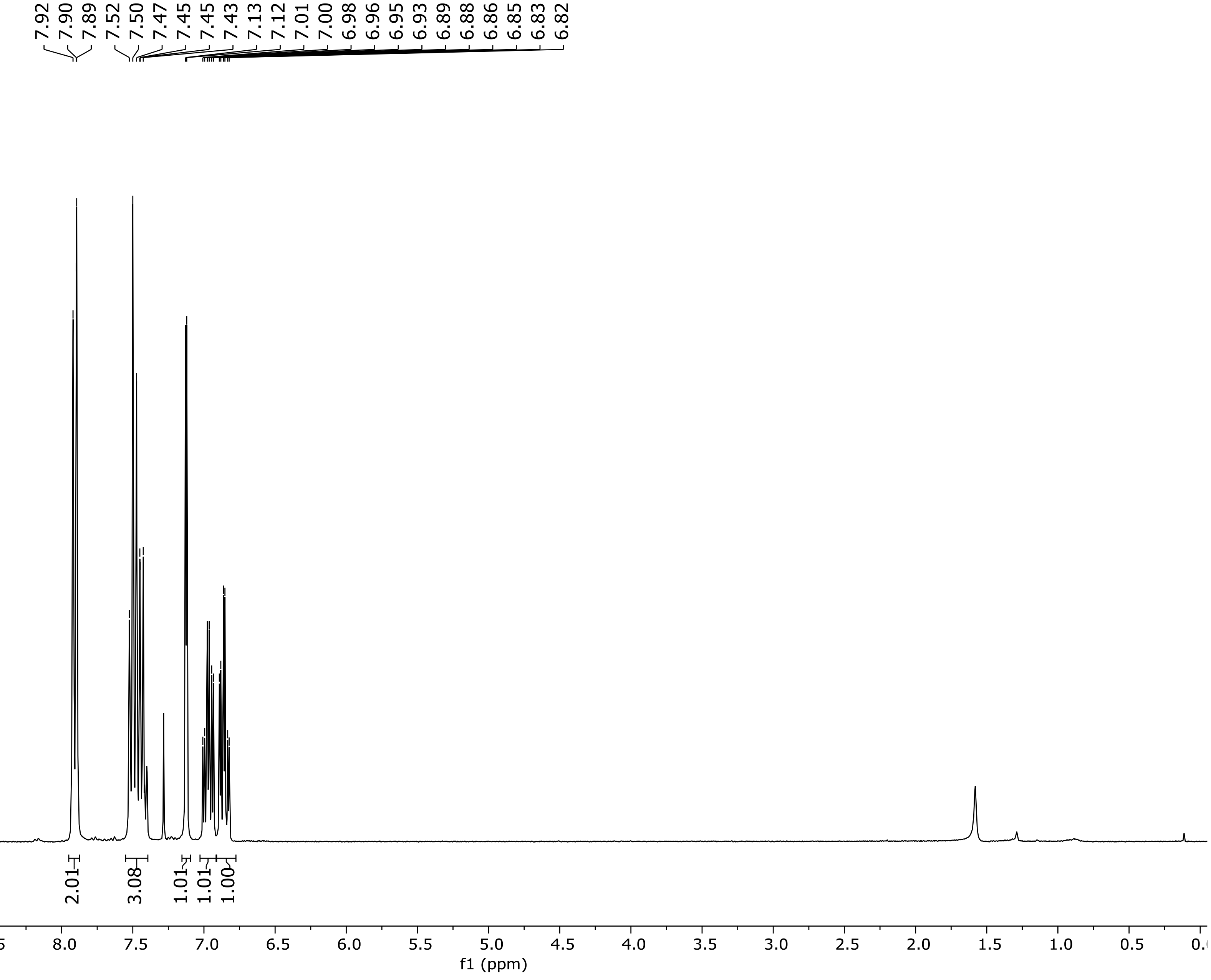

$^{13}C$ NMR (75.4 MHz, $CDCl_3$)

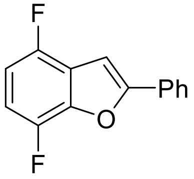


**3la**

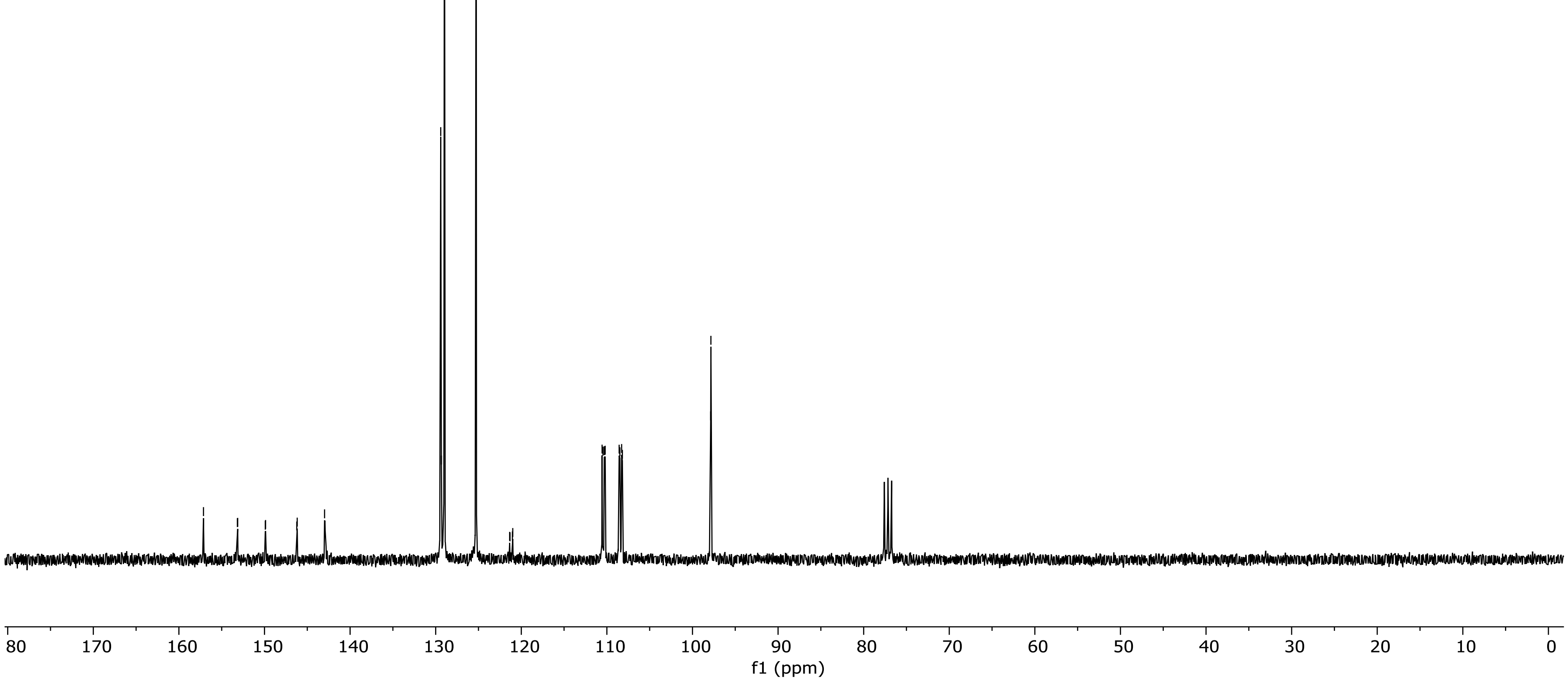

$^1$H NMR (300 MHz, $CDCl_3$)

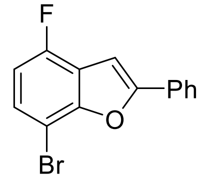


**3ma**

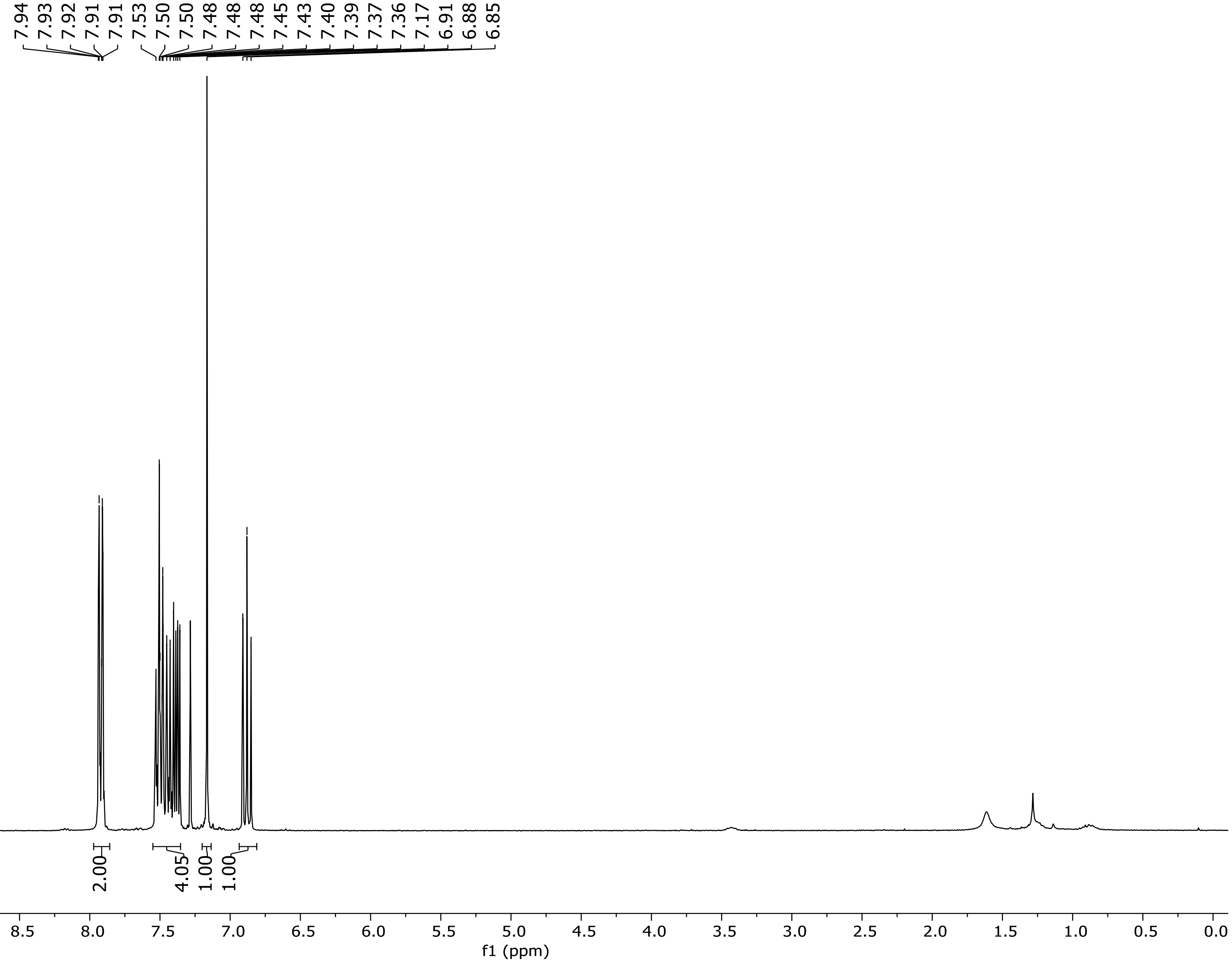

$^{13}$C NMR (75.4 MHz, $CDCl_3$)

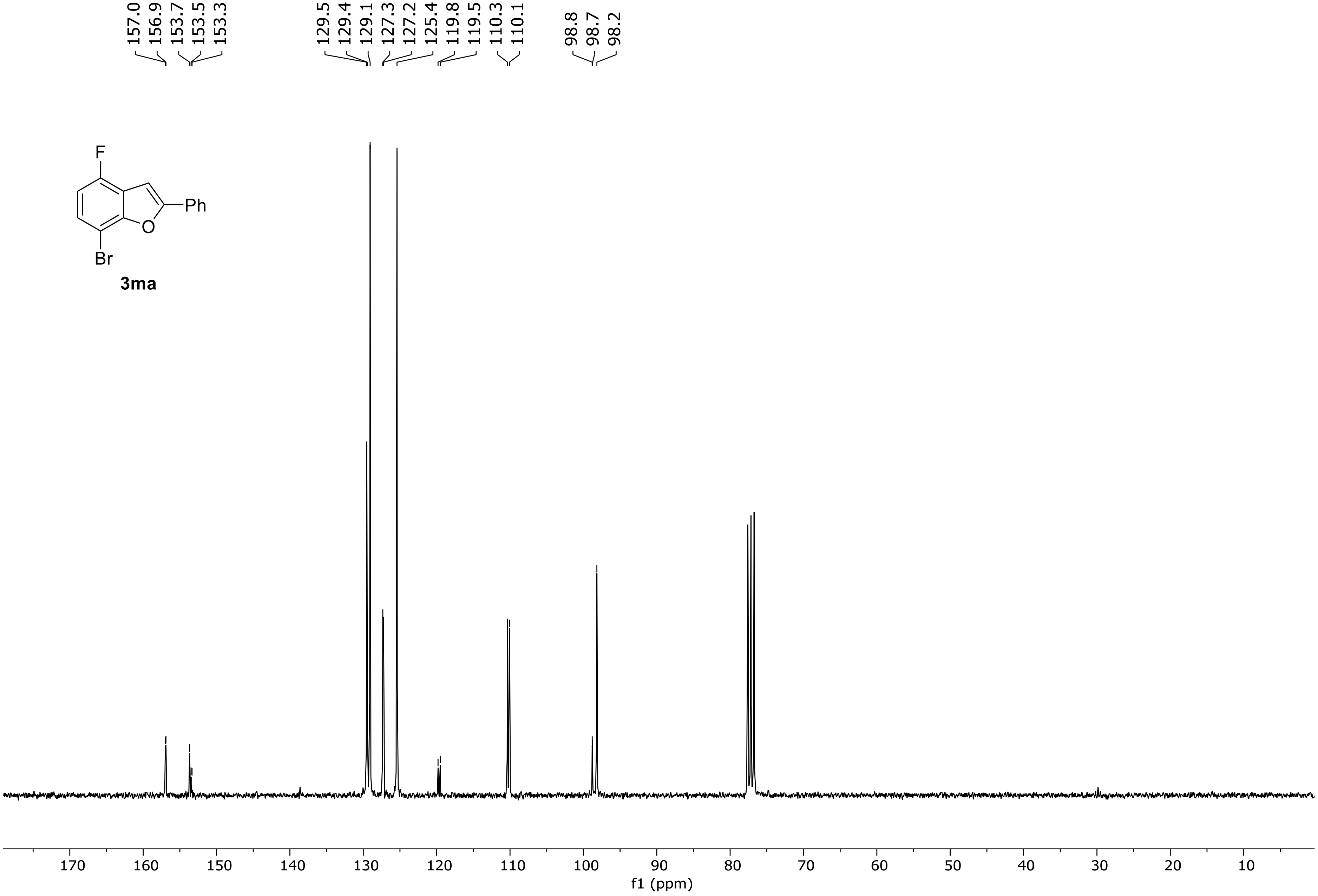

$^1$H NMR (300 MHz, $CDCl_3$)

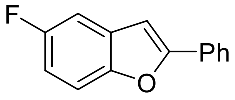

**4oa**

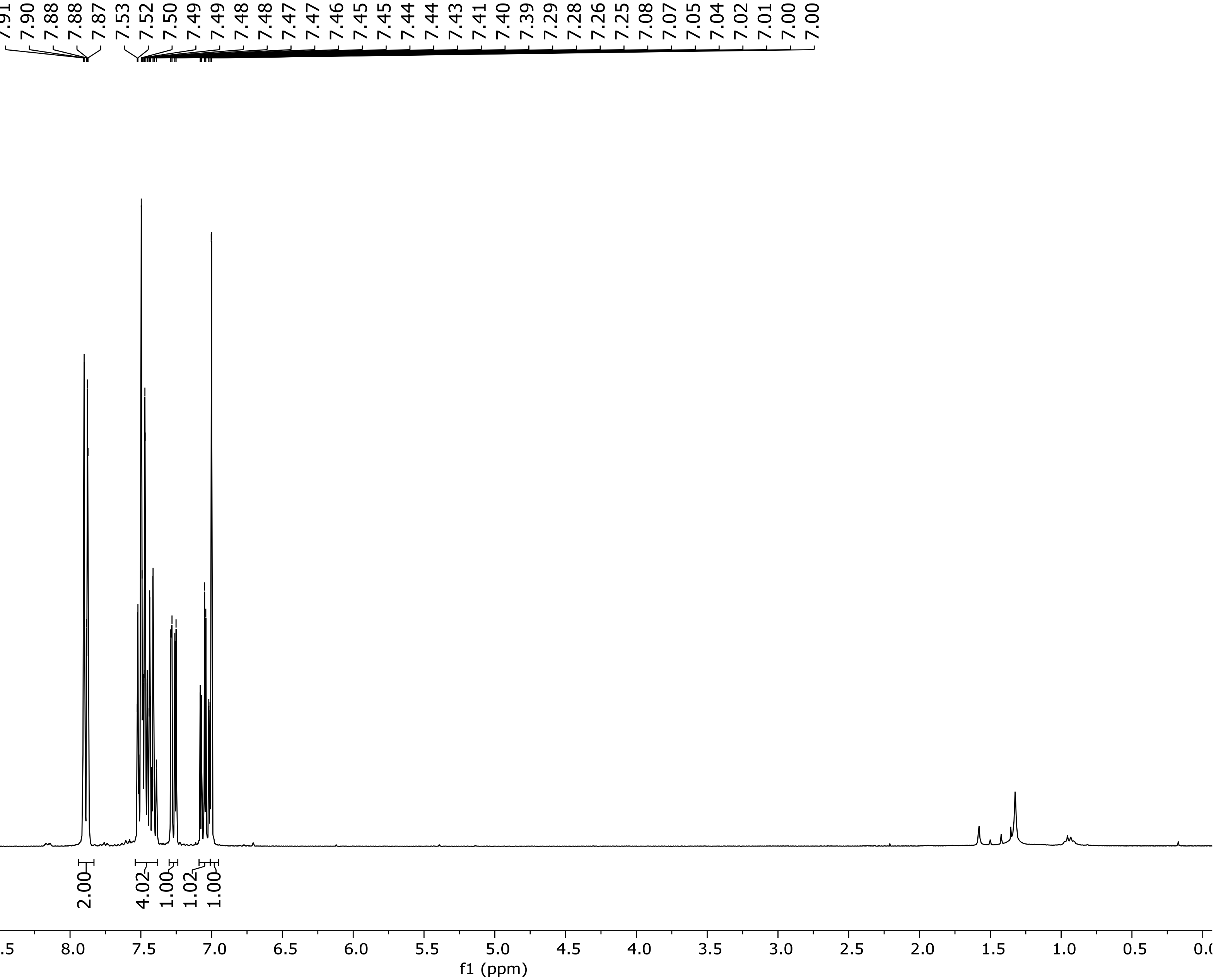

$^{13}$C NMR (75.4 MHz, $CDCl_3$)

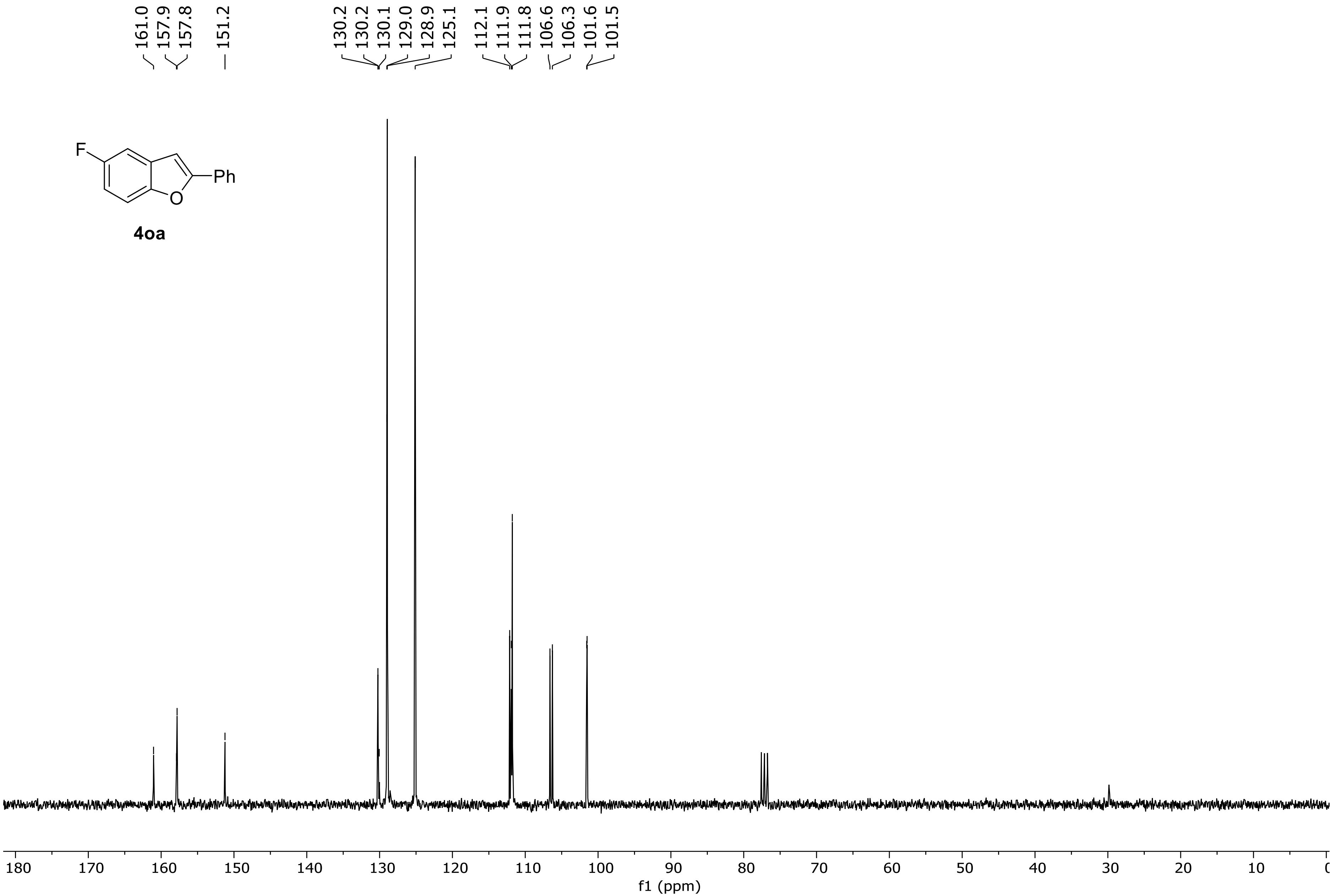

$^1$H NMR (300 MHz, $CDCl_3$)

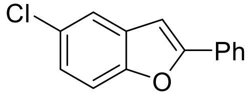


**4pa**

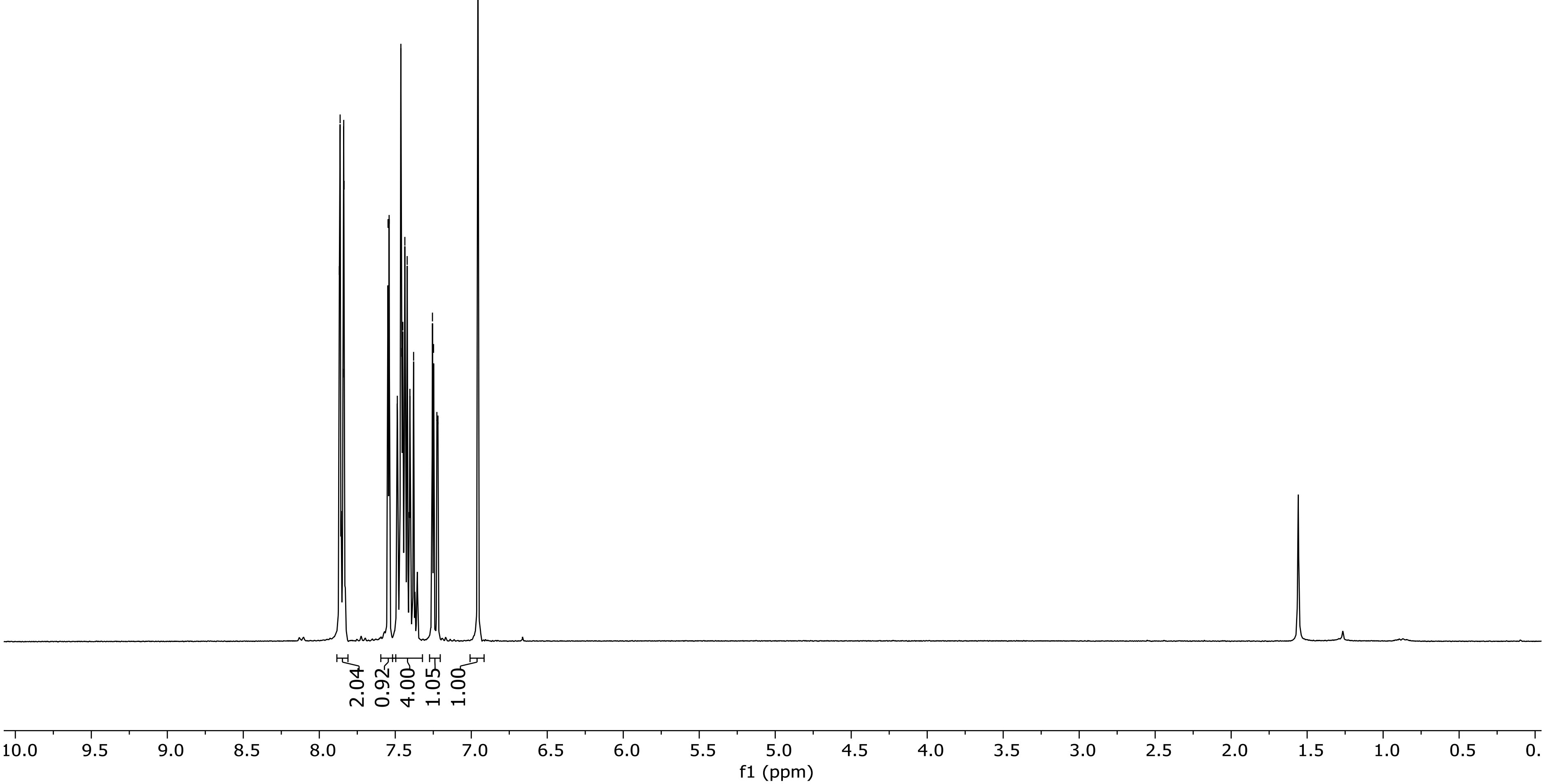

$^{13}C$ NMR (75.4 MHz, $CDCl_3$)

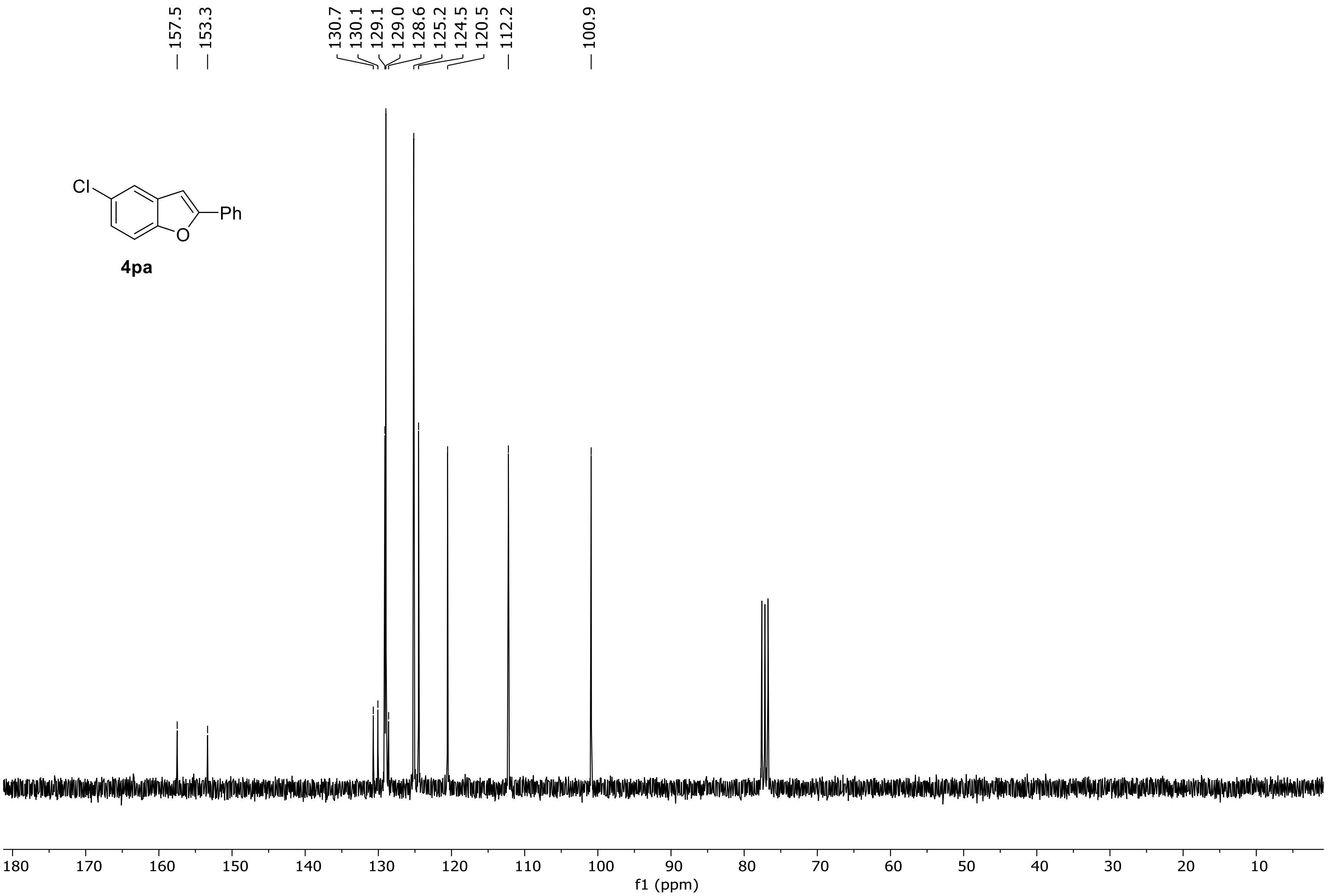


**4pa**

$^{1}H$ NMR (300 MHz, $CDCl_3$)

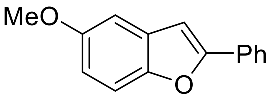


**4qa**

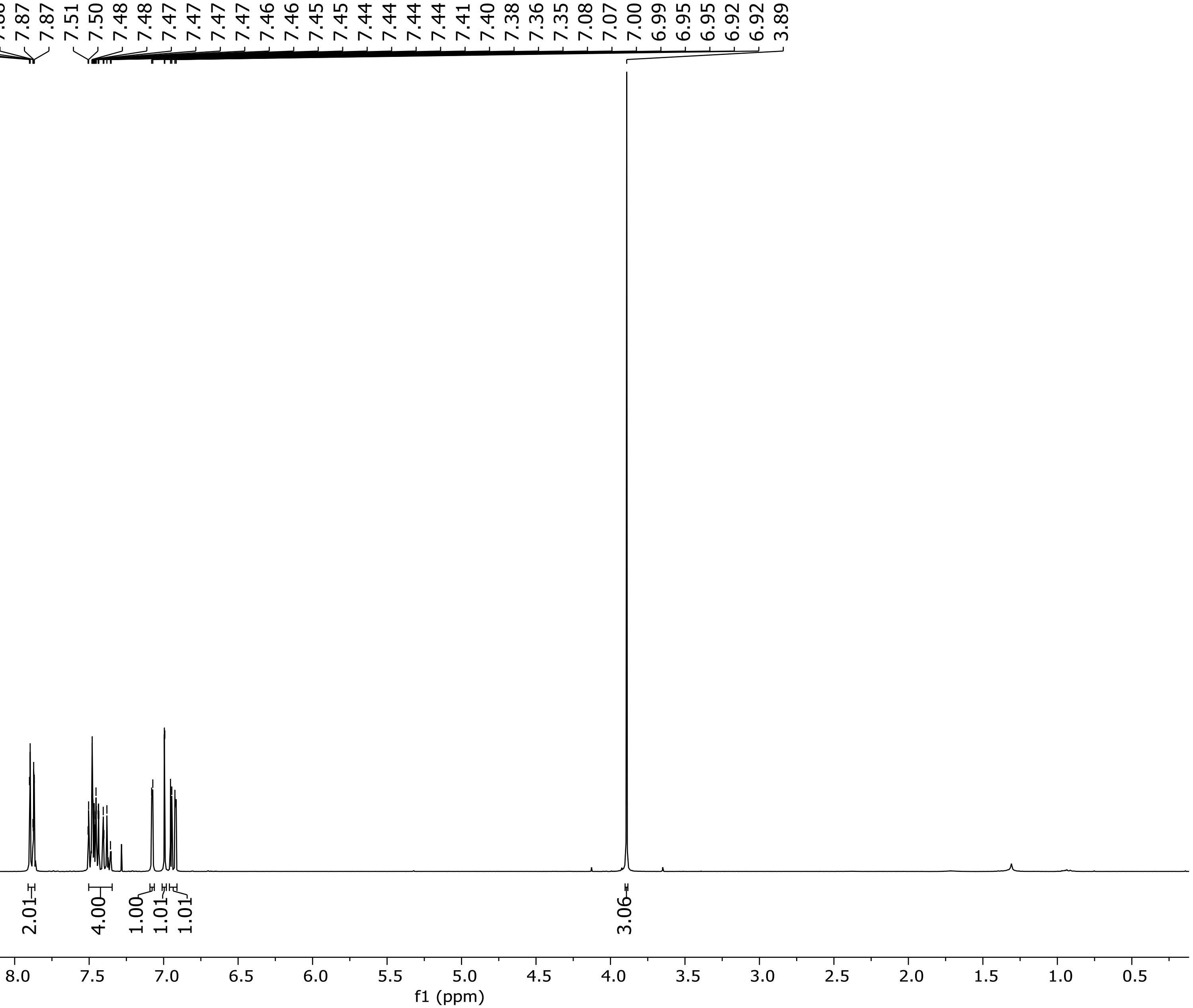

$^{13}$C NMR (75.4 MHz, $CDCl_3$)

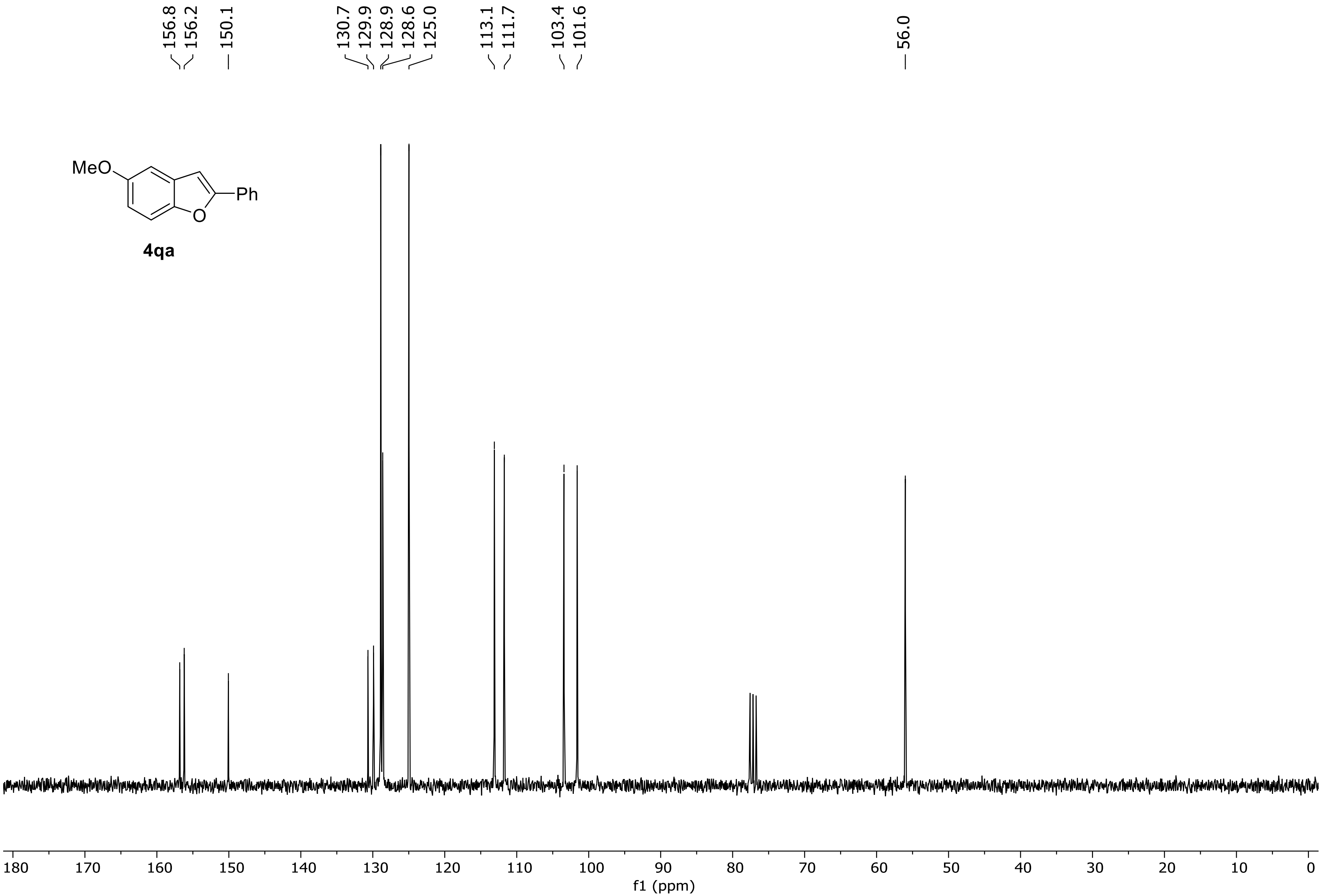

¹H NMR (300 MHz, $CDCl_3$)

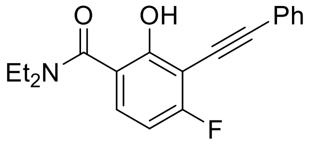


**5aa**

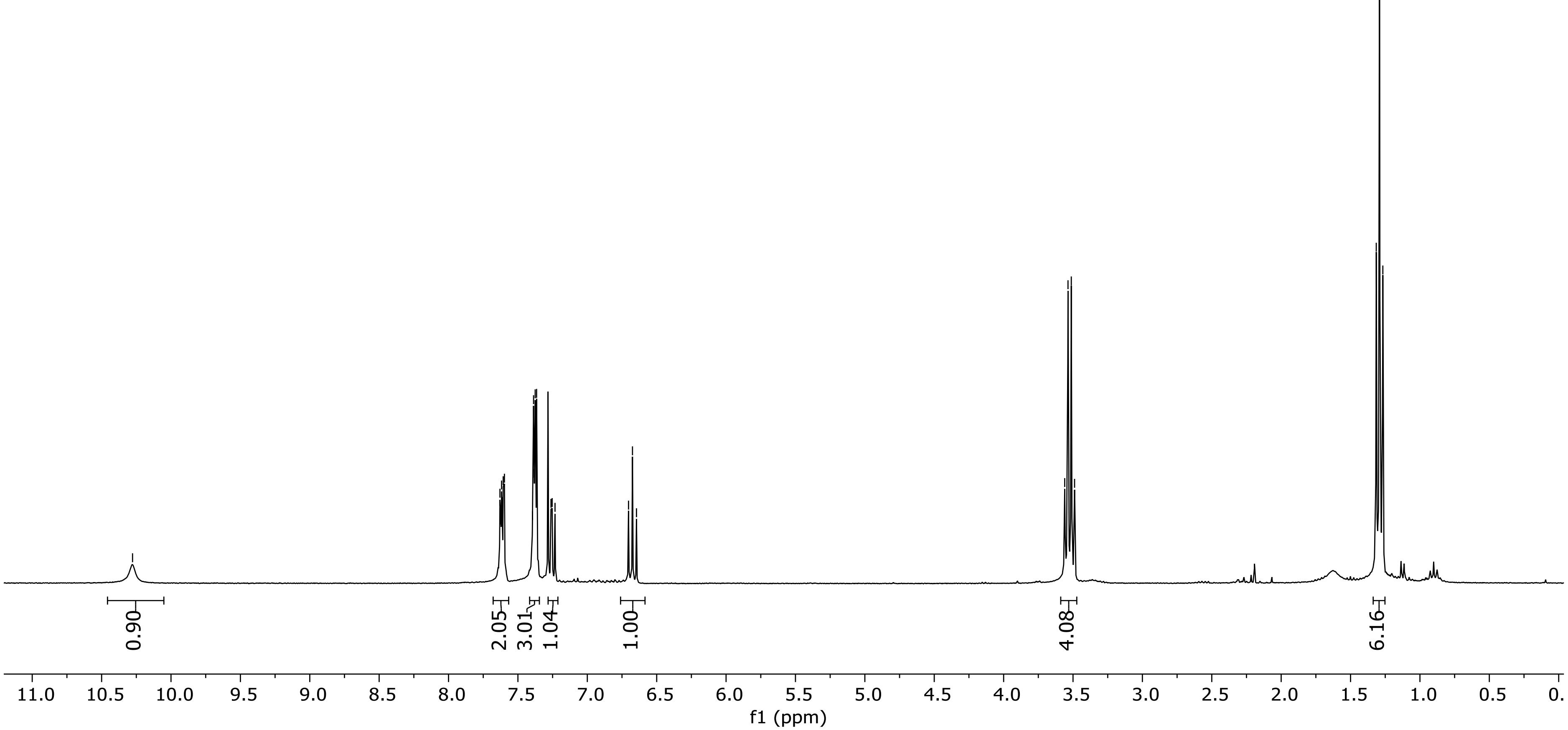

13C NMR (75.4 MHz, $CDCl_3$)

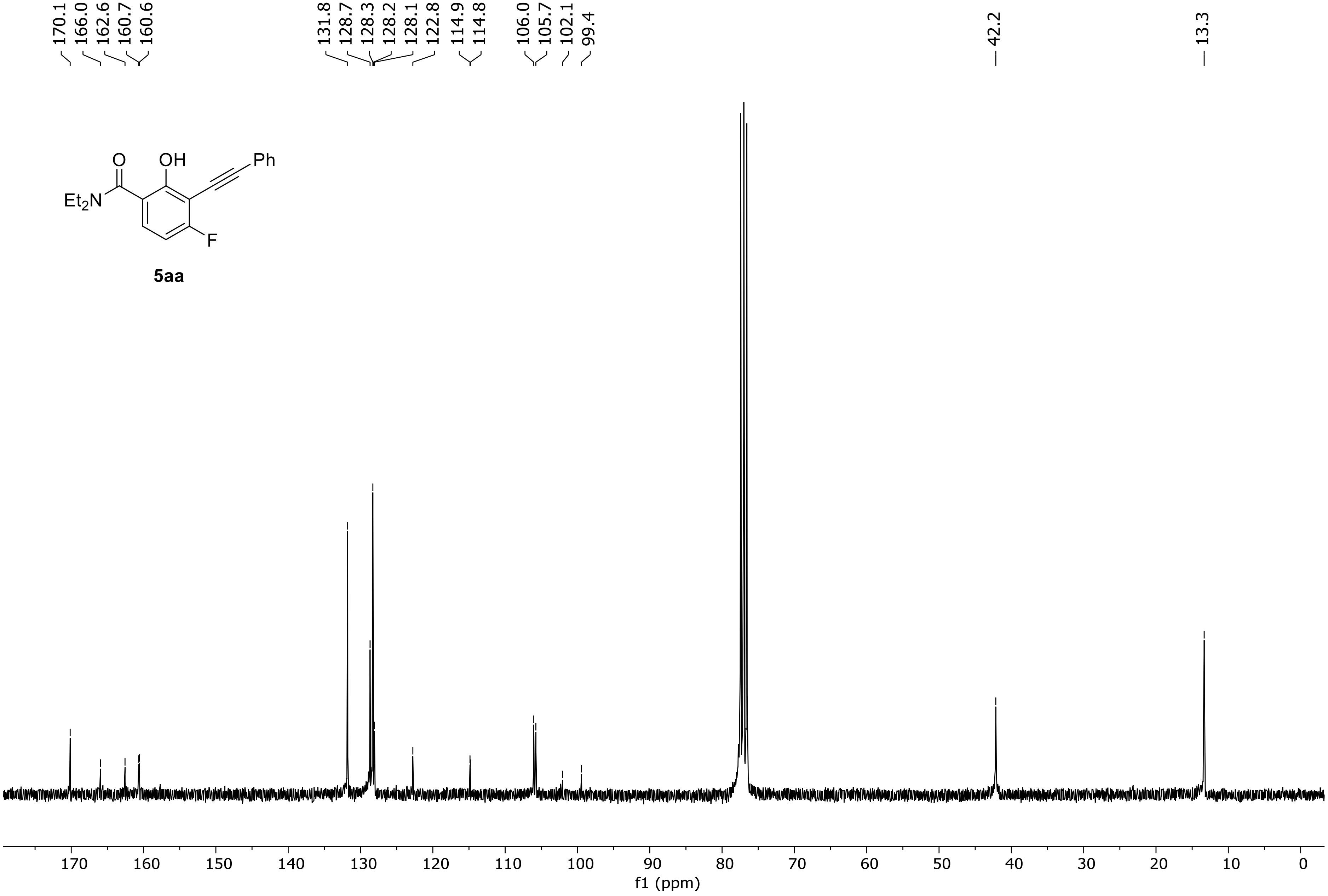

¹H NMR (300 MHz, $CDCl_3$)

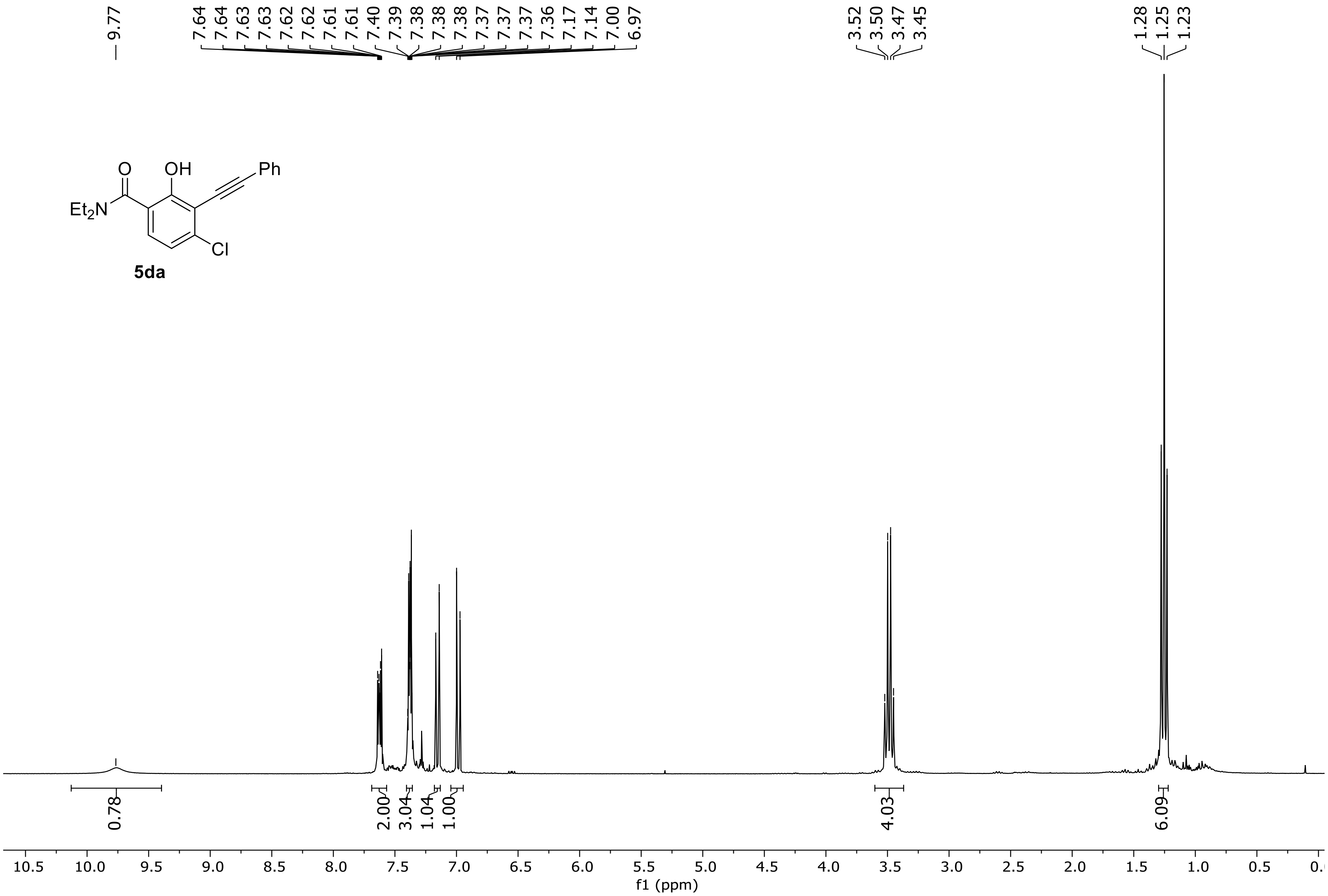

$^{13}C$ NMR (75.4 MHz, $CDCl_3$)

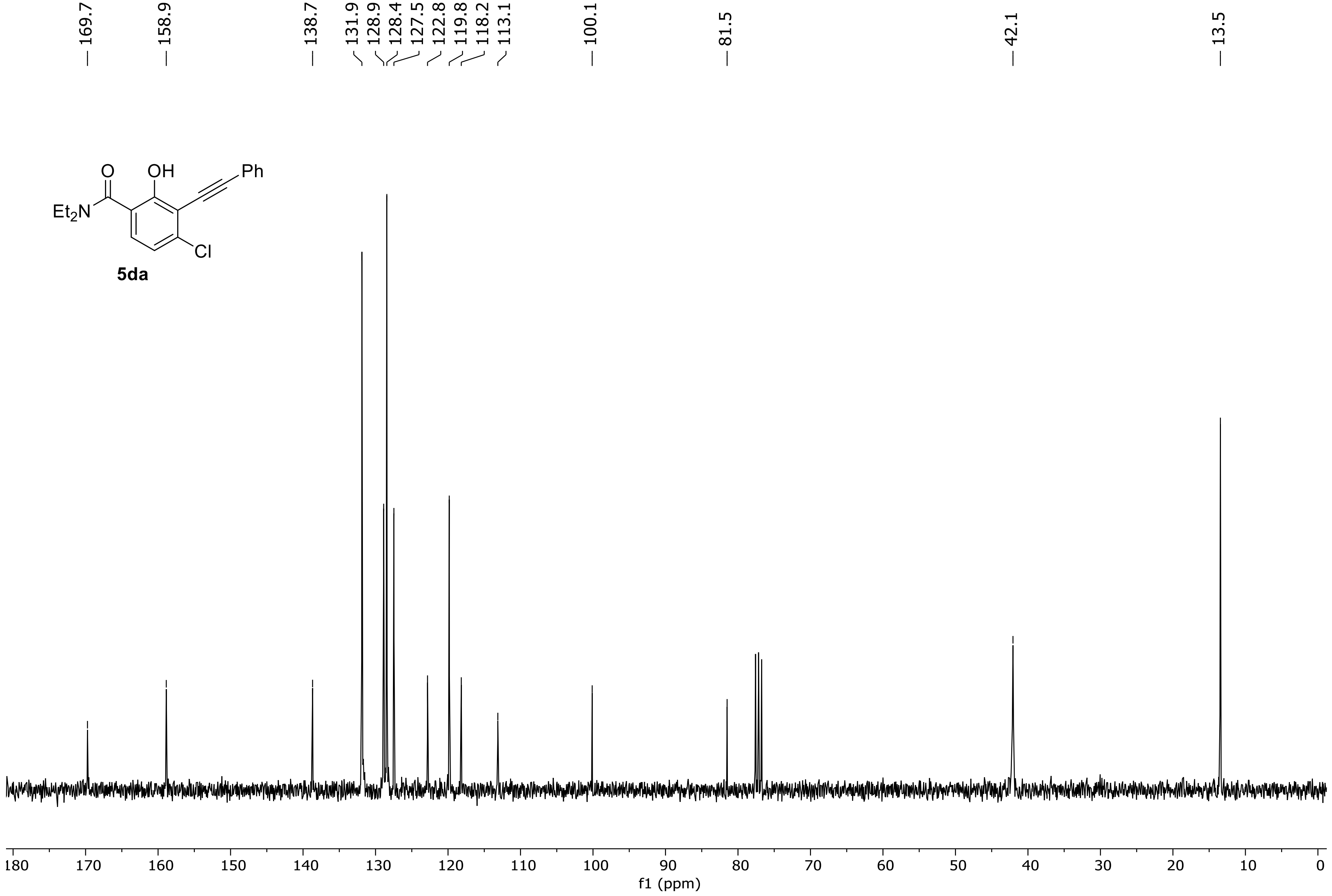


**5da**

$^1$H NMR (300 MHz, $CDCl_3$)

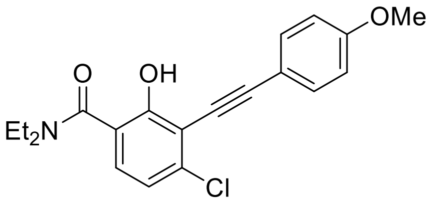


**5db**

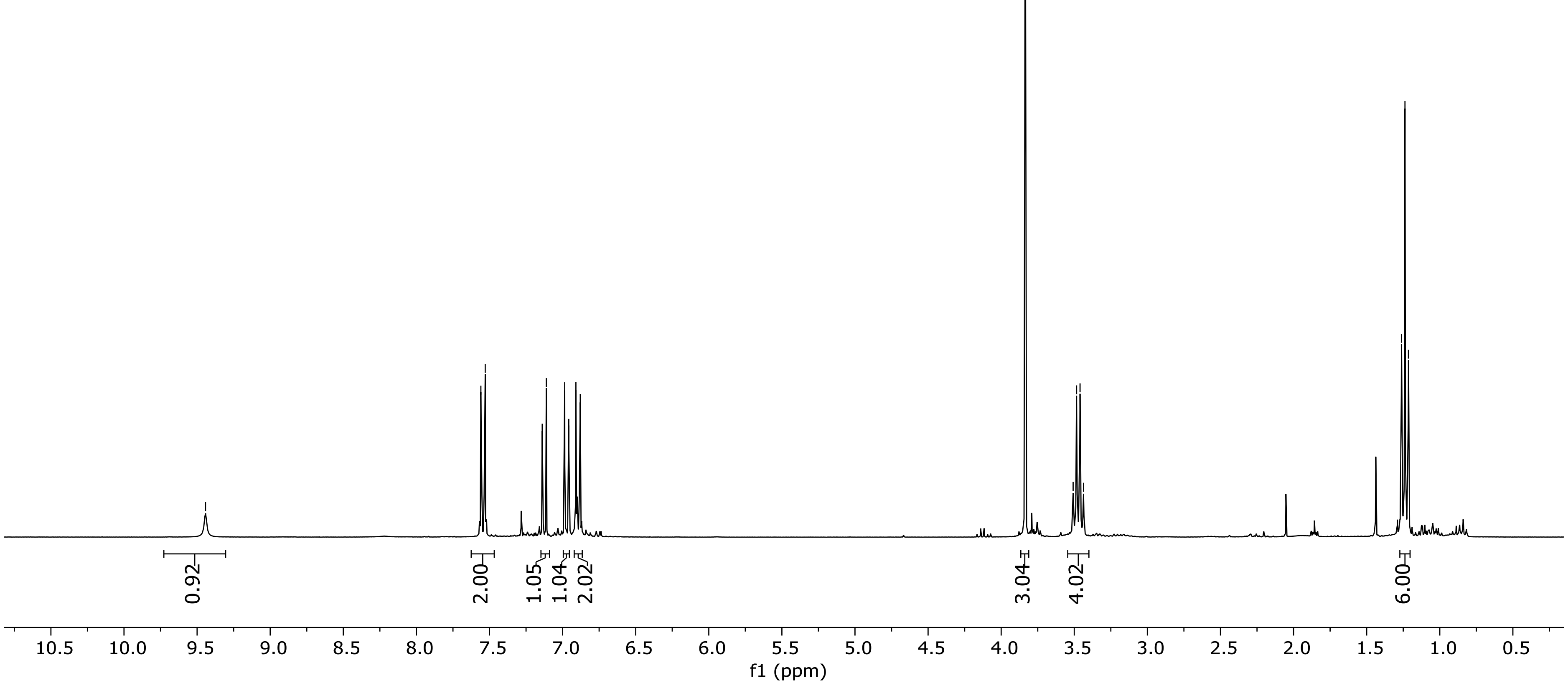

$^{13}C$ NMR (75.4 MHz, $CDCl_3$)

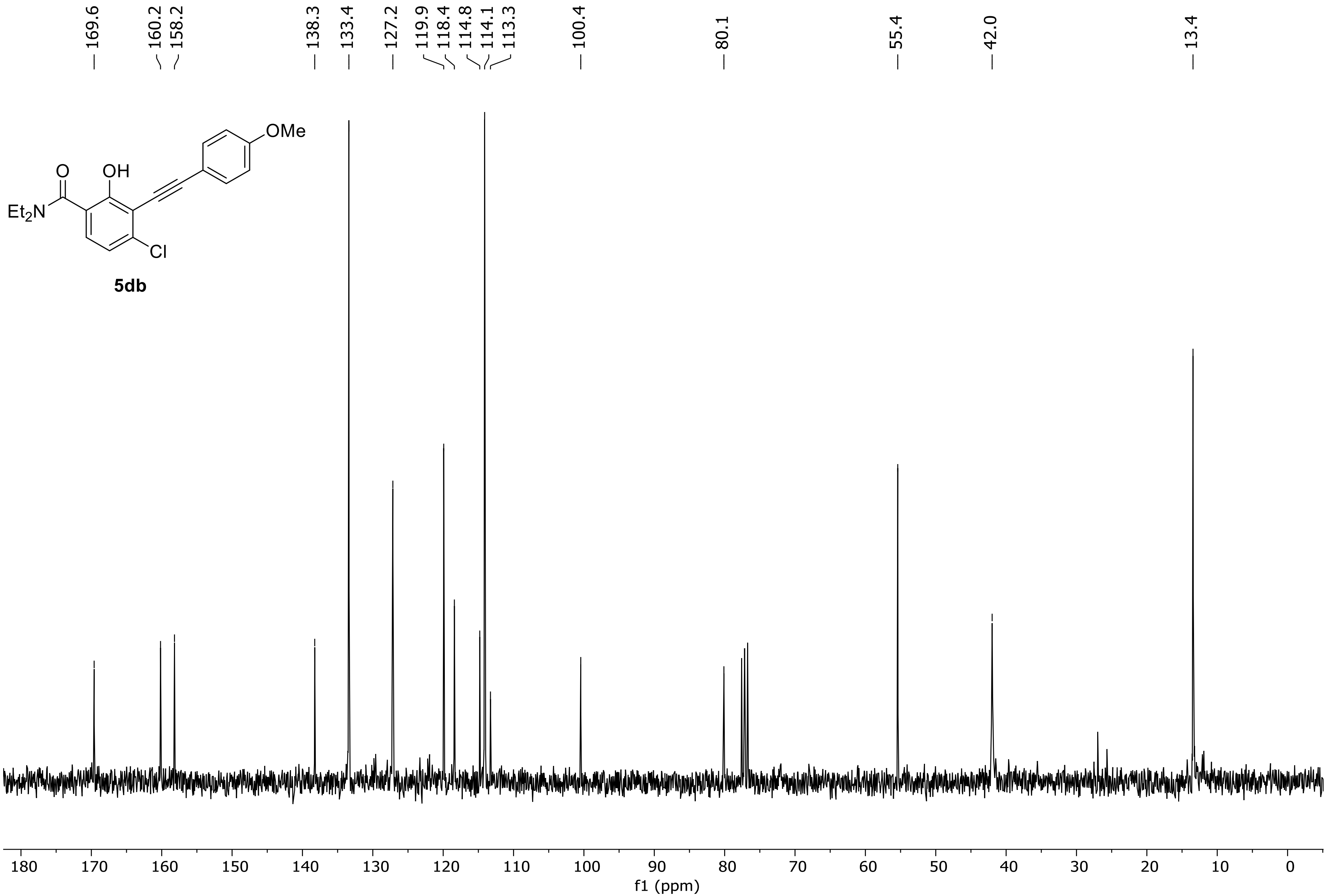

$^{1}H$ NMR (300 MHz, $CDCl_3$)

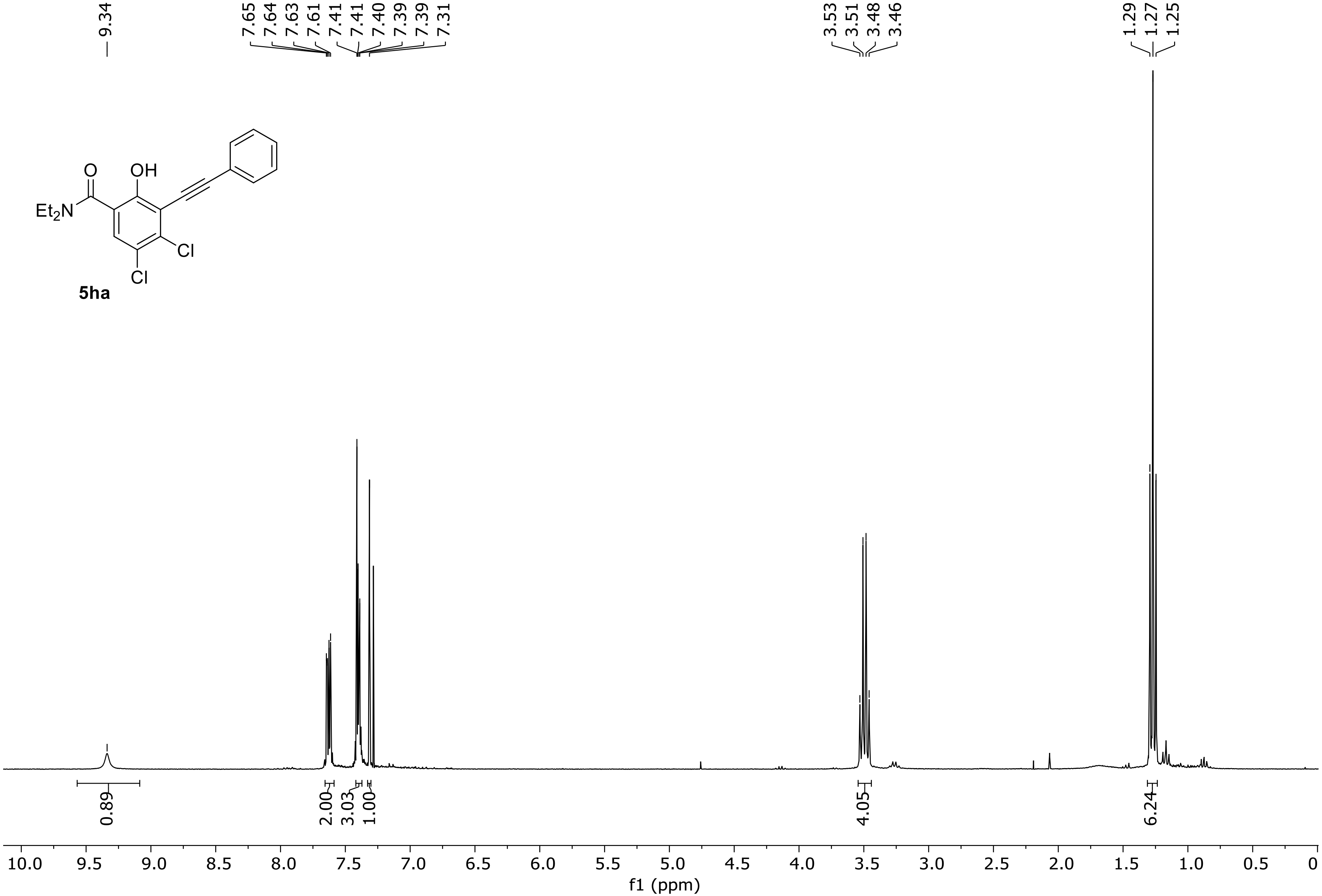

$^{13}C$ NMR (75.4 MHz, $CDCl_3$)

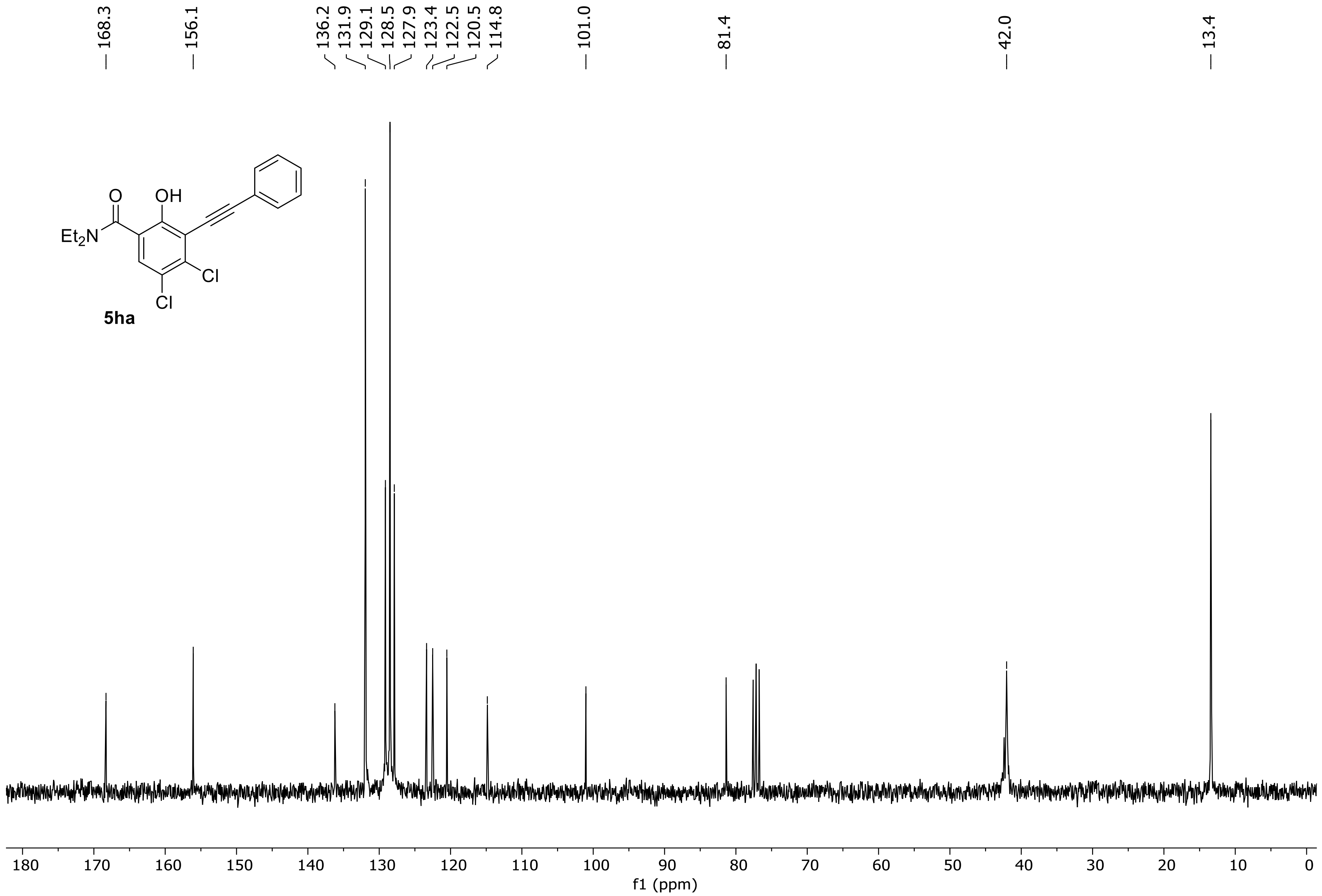

$^{1}$H NMR (300 MHz, $CDCl_3$)

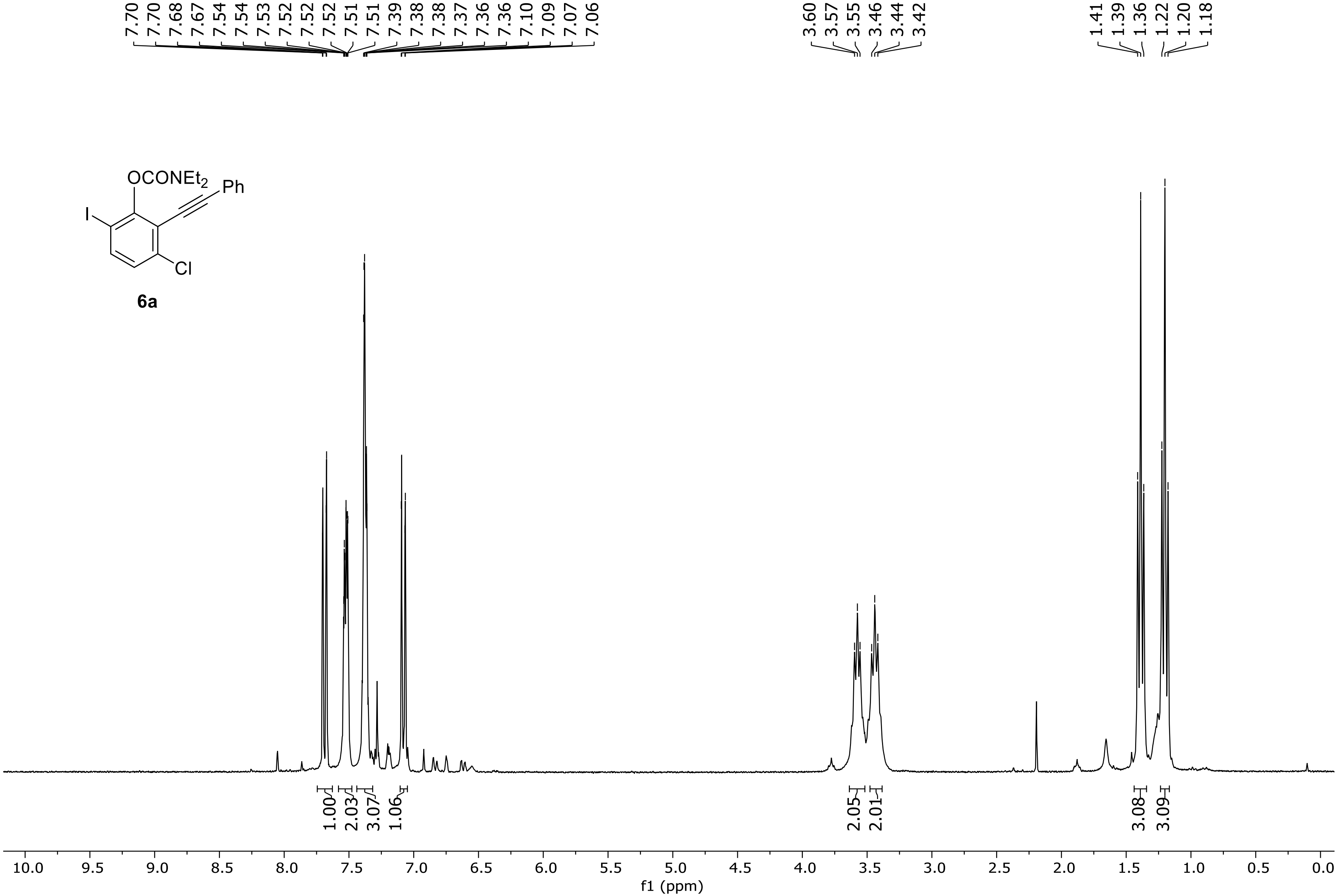

$^{13}C$ NMR (75.4 MHz, $CDCl_3$)

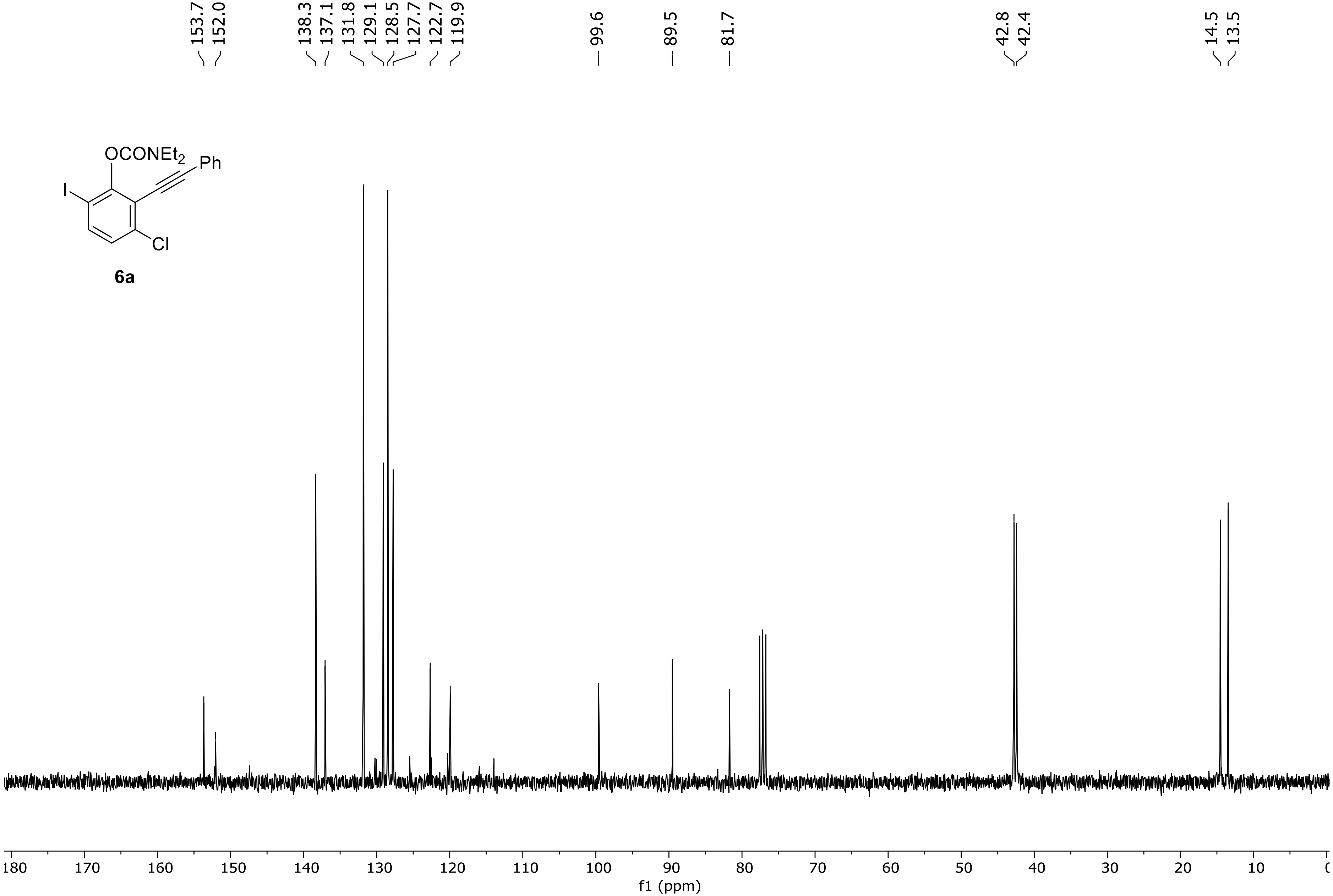

$^1H$ NMR (300 MHz, $CDCl_3$)

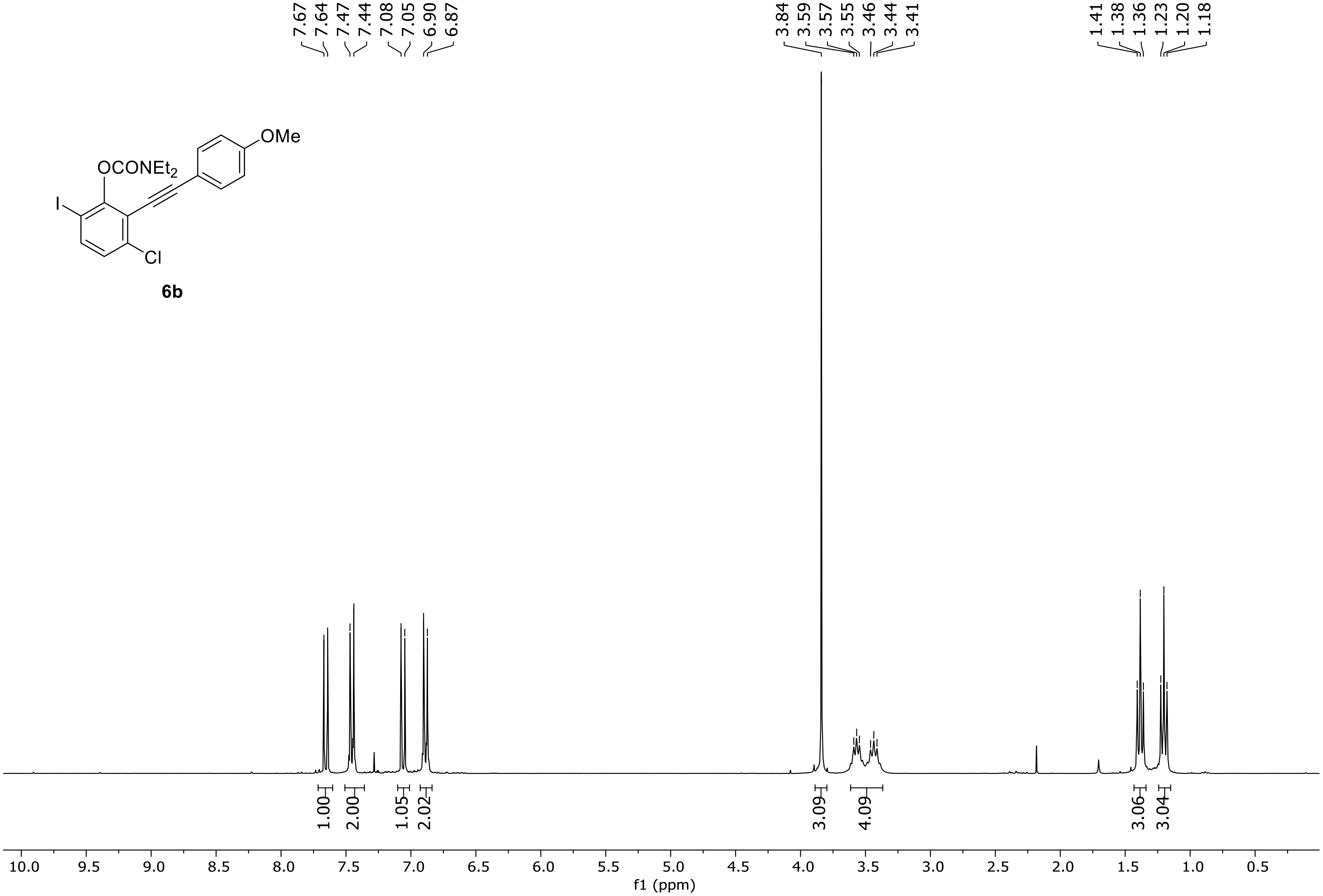


**6b**

$^{13}C$ NMR (75.4 MHz, $CDCl_3$)

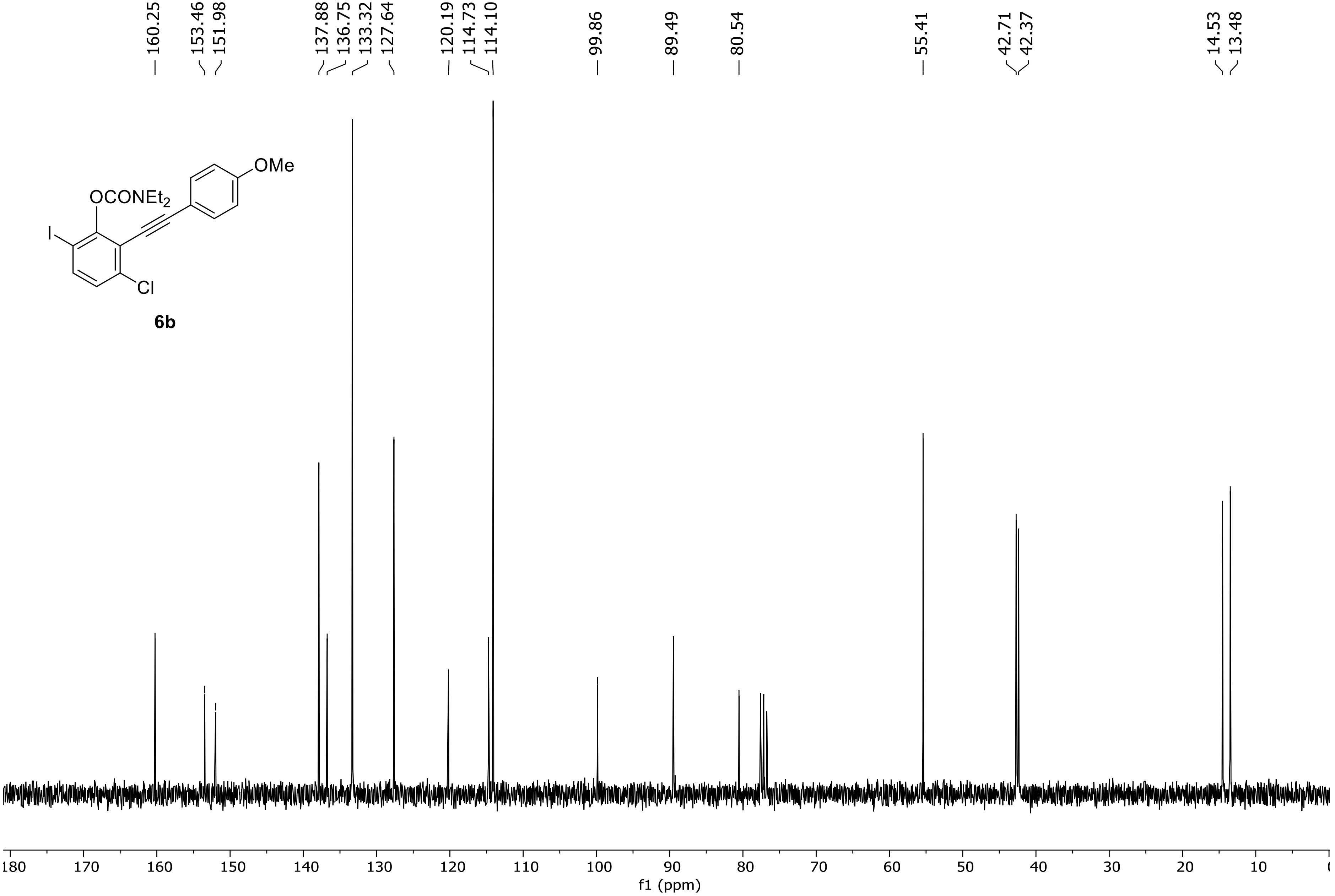

$^{1}H$ NMR (300 MHz, $CDCl_3$)

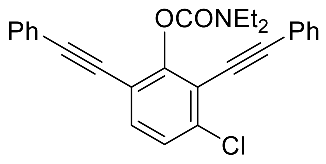


**7aa**

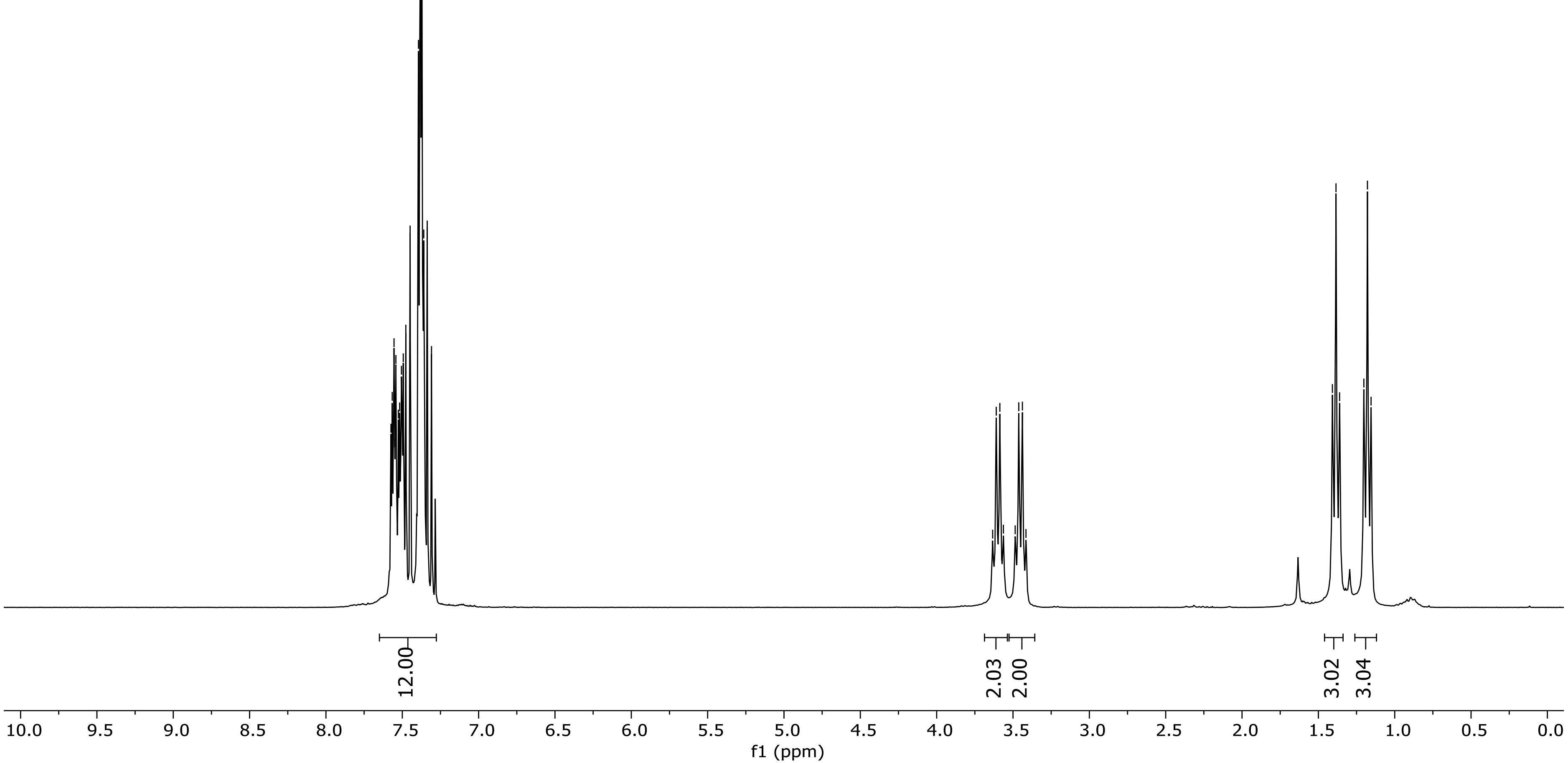

$^{13}C$ NMR (75.4 MHz, $CDCl_3$)

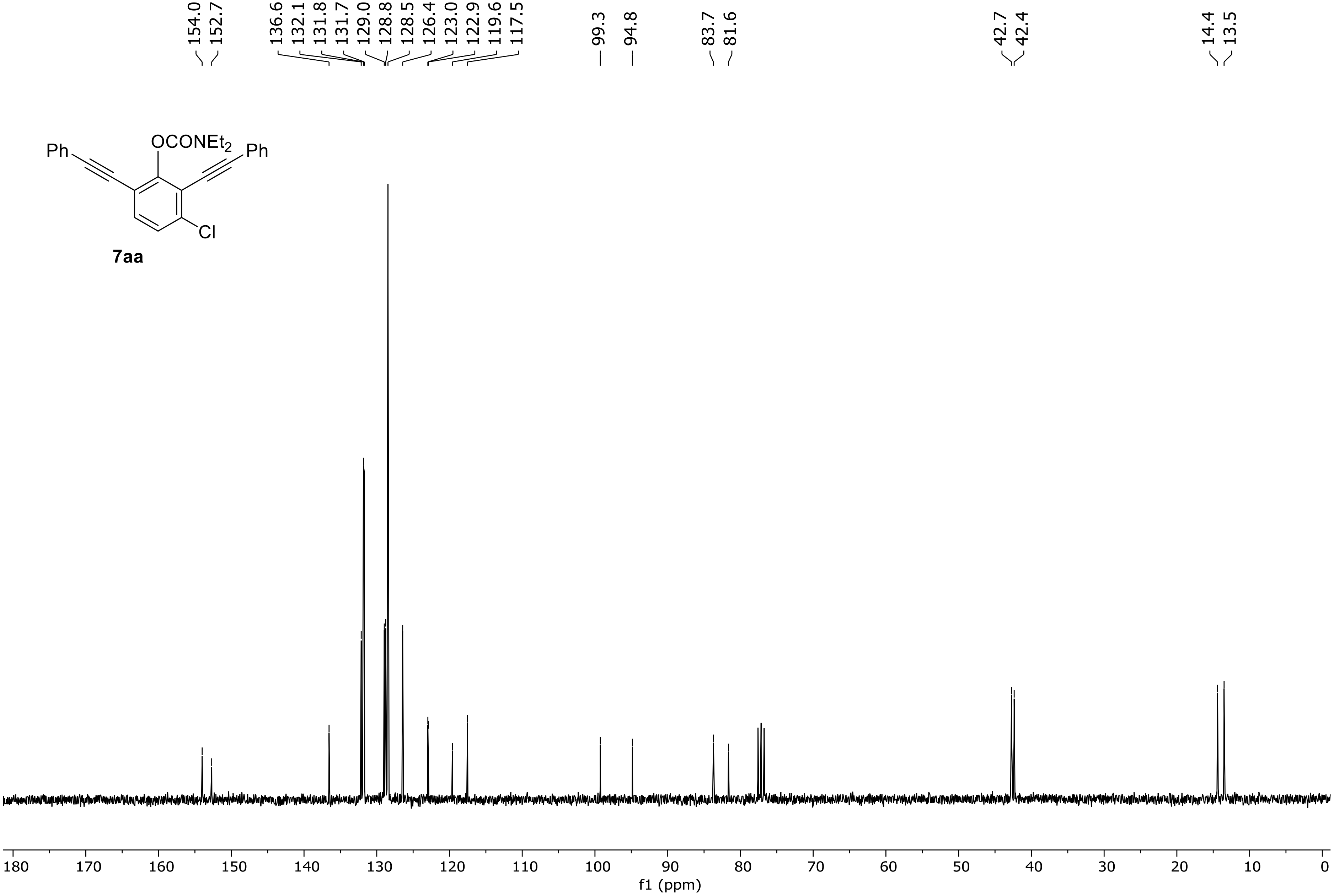

$^{1}$H NMR (300 MHz, $CDCl_3$)

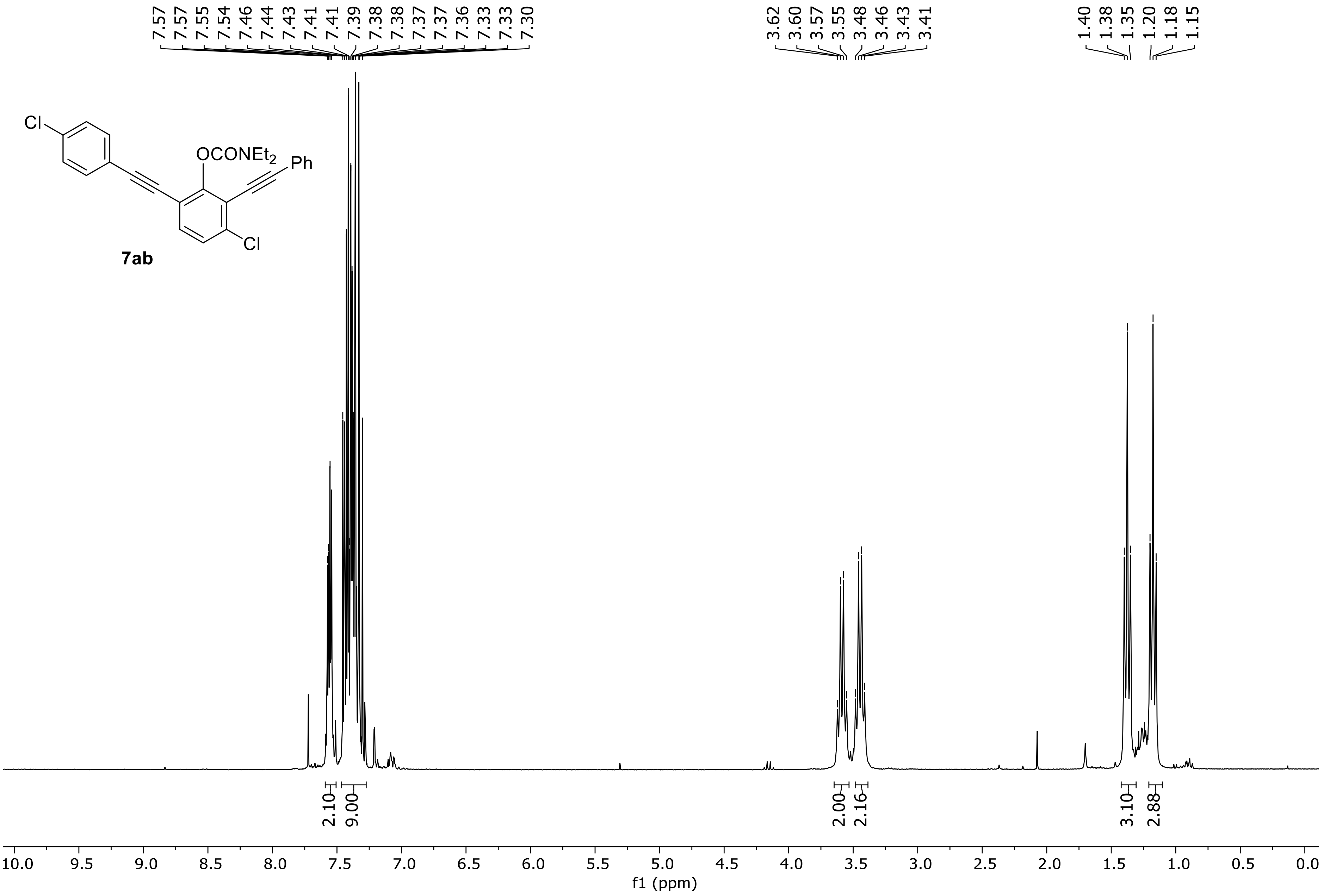

$^{13}C$ NMR (75.4 MHz, $CDCl_3$)

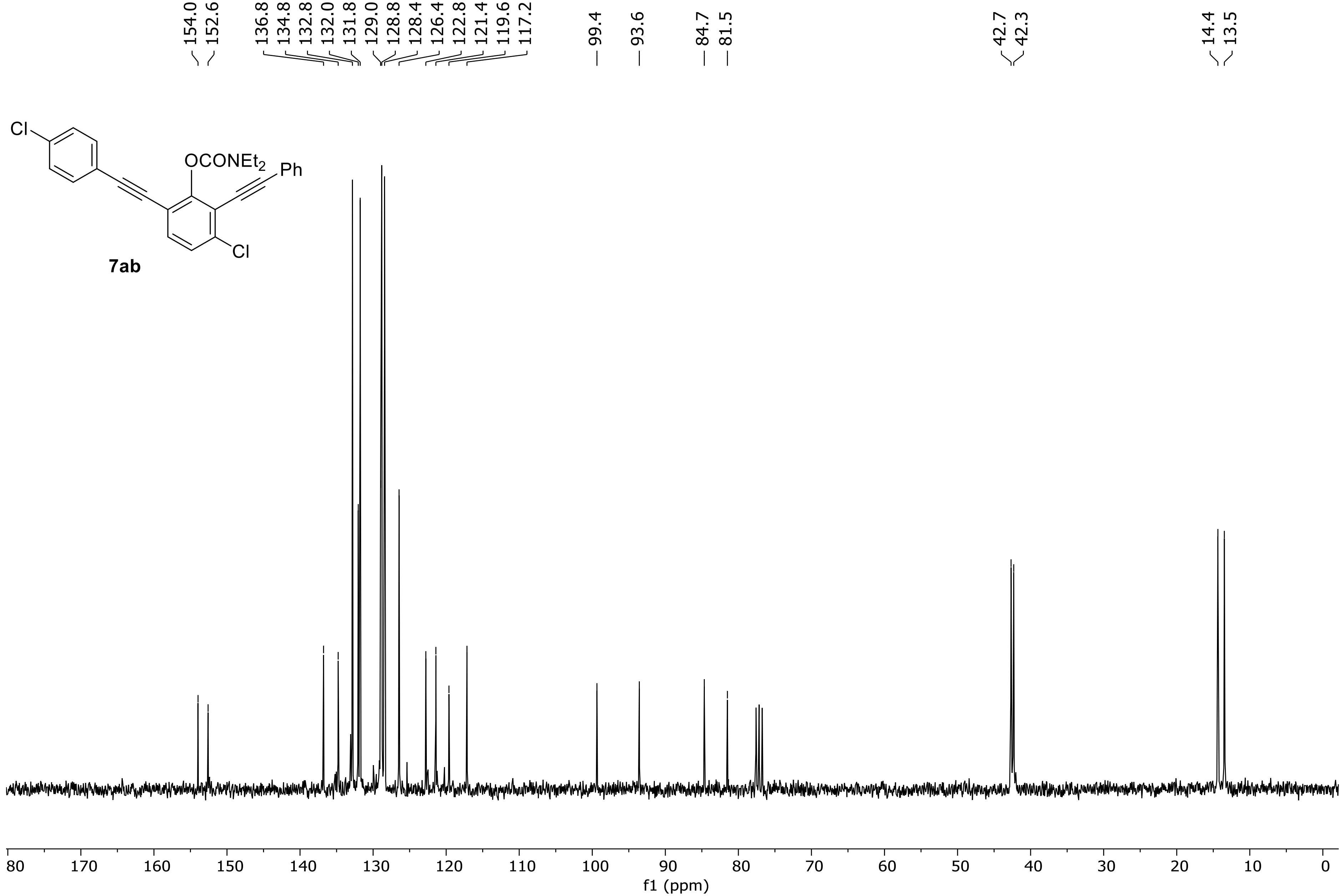

$^{1}H$ NMR (300 MHz, $CDCl_3$)

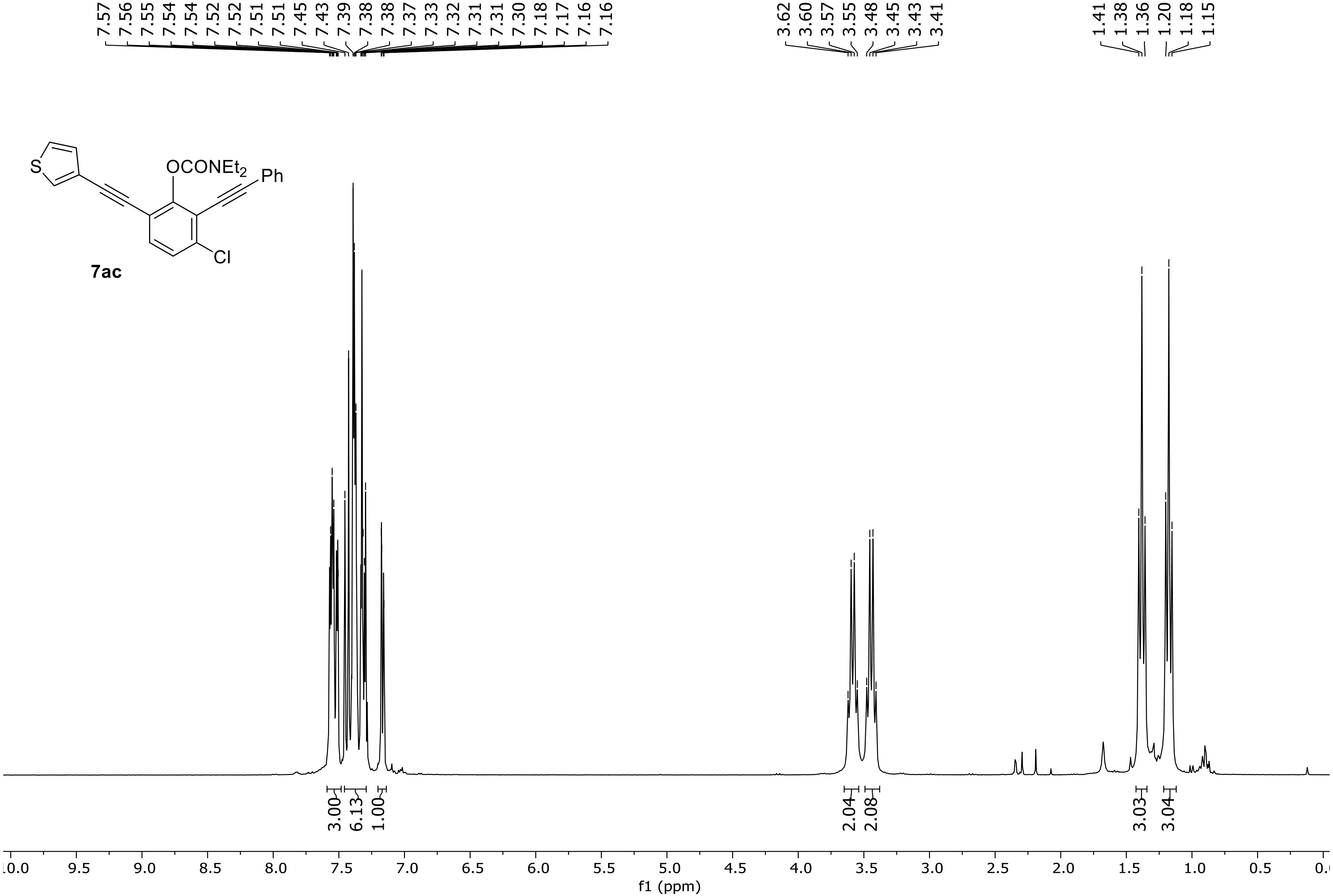


**7ac**

$^{13}C$ NMR (75.4 MHz, $CDCl_3$)

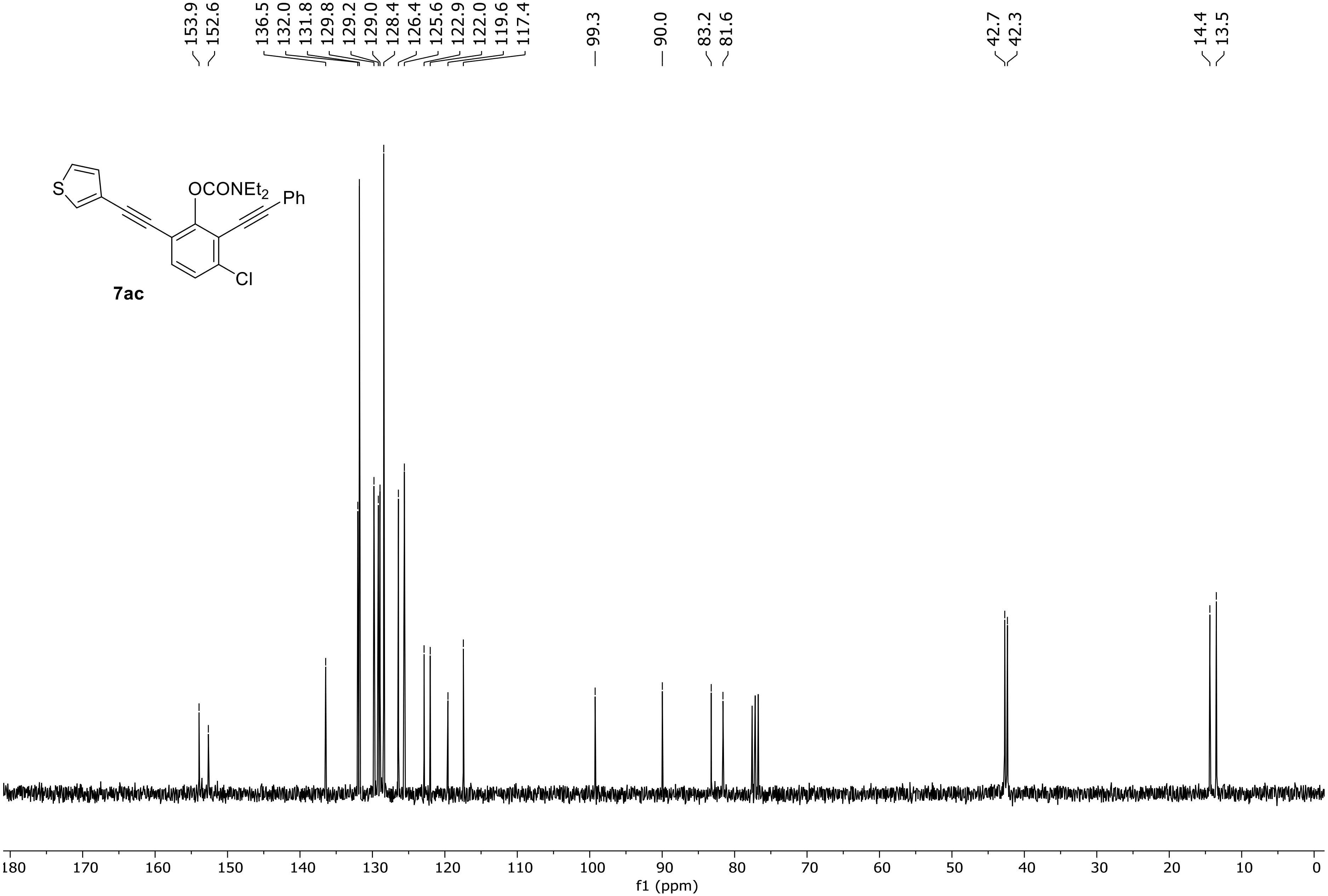

$^{1}$H NMR (300 MHz, $CDCl_3$)

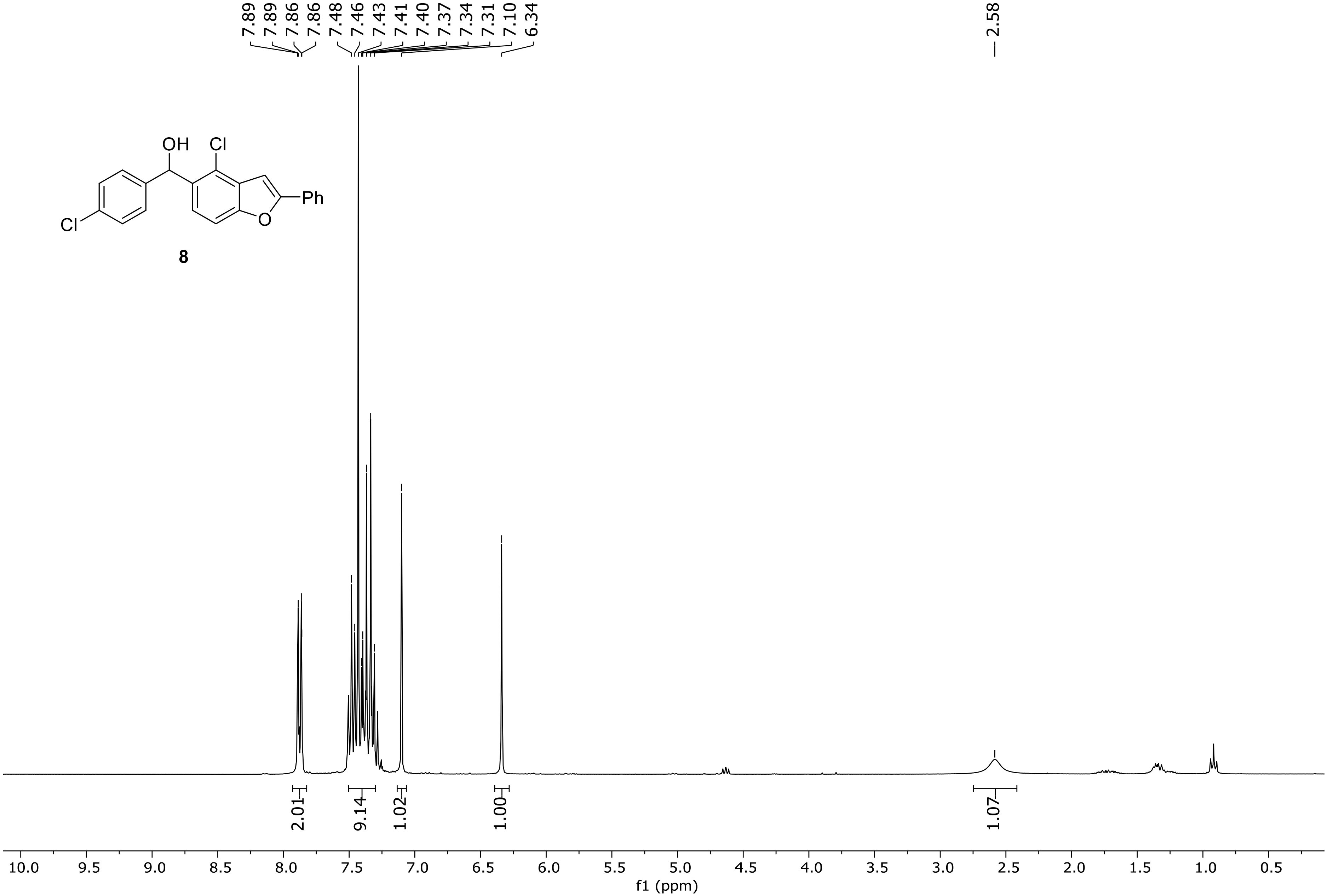

8

$^{13}C$ NMR (75.4 MHz, $CDCl_3$)

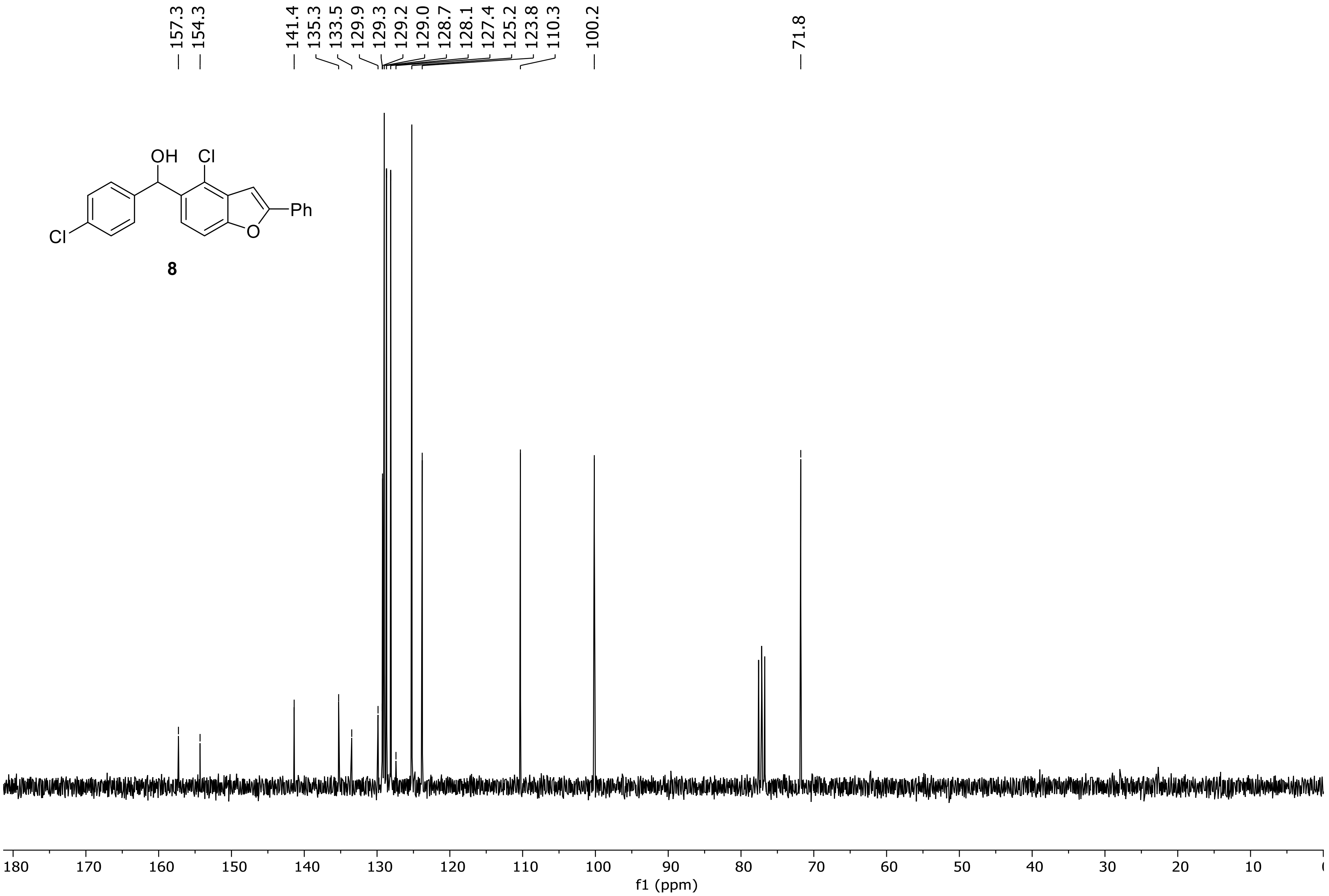

$^{1}H$ NMR (300 MHz, $CDCl_3$)

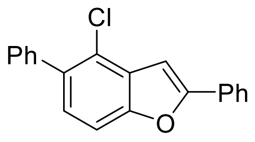


**9**

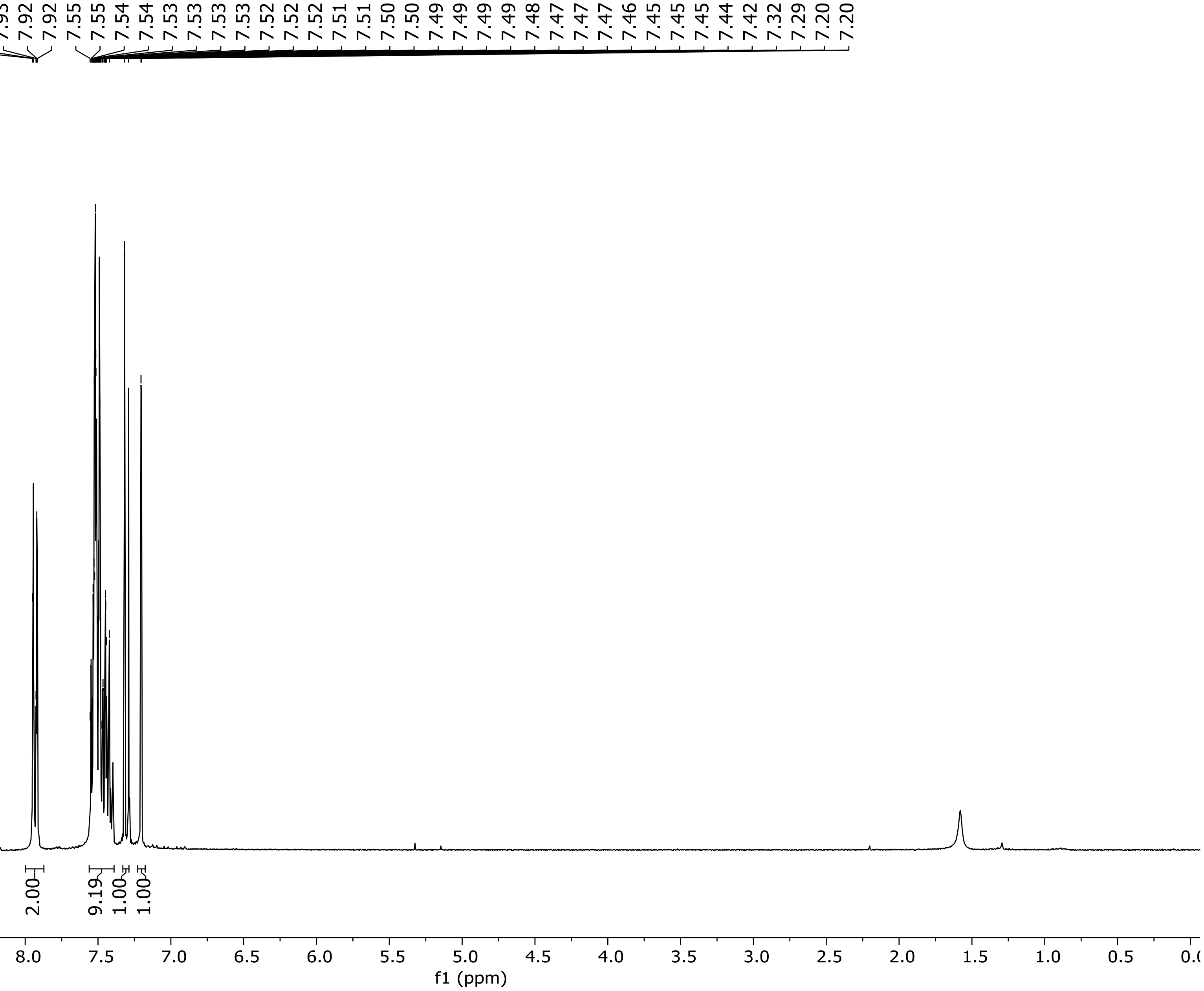

$^{13}C$ NMR (75.4 MHz, $CDCl_3$)

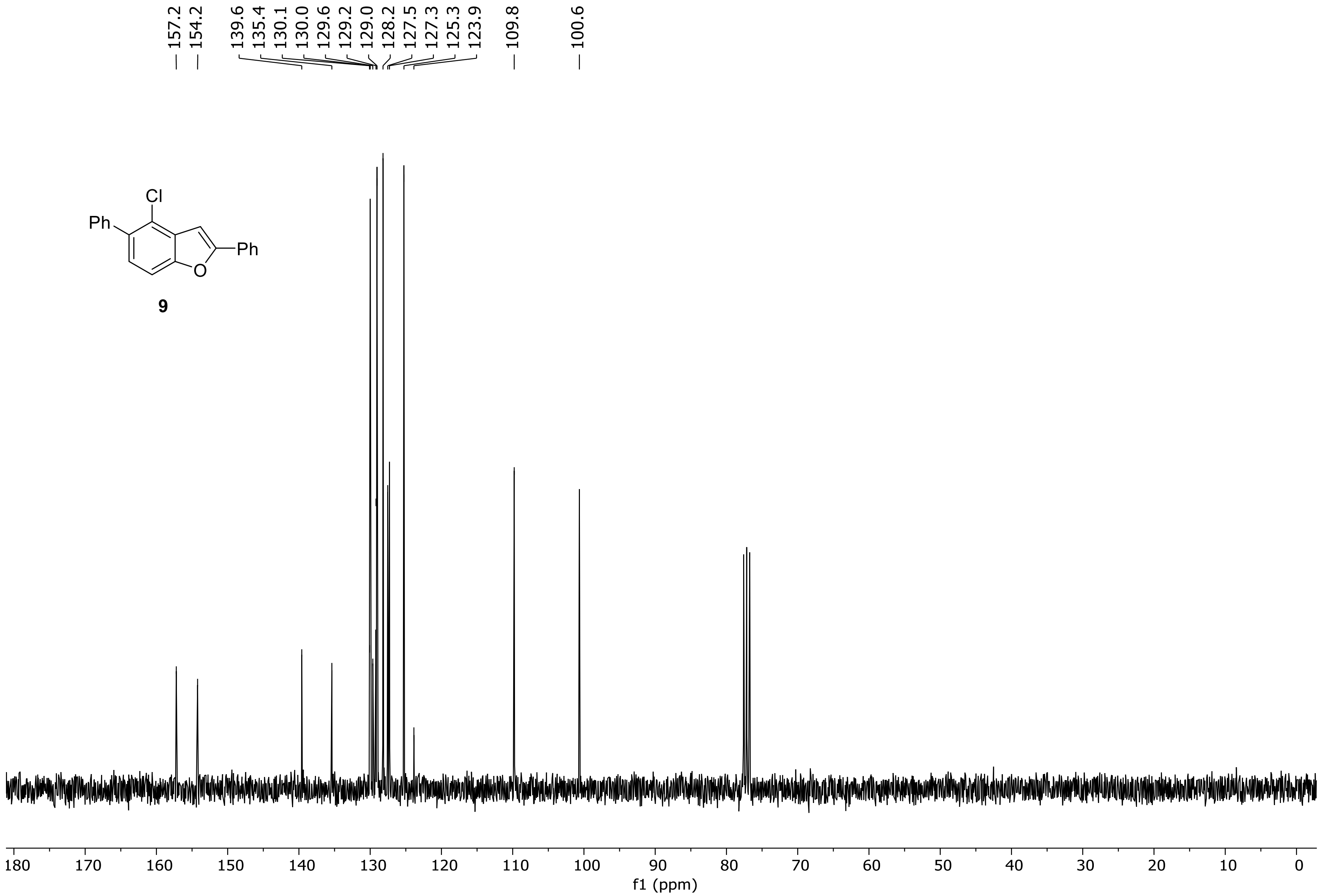

Supplement: Supplementary file 1 [file molecules-27-00525-s001.zip › molecules-1543280-supplementary.pdf]
